# Limonoids from *Guarea guidonia* and *Cedrela odorata*: Heat Shock Protein 90 (Hsp90) Modulator Properties of Chisomicine D

**DOI:** 10.1021/acs.jnatprod.0c01217

**Published:** 2021-03-04

**Authors:** Maria Laura Bellone, Cesar Muñoz Camero, Maria Giovanna Chini, Fabrizio Dal Piaz, Vanessa Hernandez, Giuseppe Bifulco, Nunziatina De Tommasi, Alessandra Braca

**Affiliations:** † Dipartimento di Farmacia, Università degli Studi di Salerno, 84084 Fisciano (SA), Italy; ‡ PhD Program in Drug Discovery and Development, Department of Pharmacy, Università degli Studi di Salerno, 84084 Fisciano (SA), Italy; § Dipartimento di Farmacia, Università di Pisa, 56126 Pisa, Italy; ∥ Dipartimento di Bioscienze e Territorio, Università degli Studi del Molise, 86090 Pesche (IS), Italy; ⊥ Dipartimento di Medicina, Chirurgia e Odontoiatria “Scuola Medica Salernitana”, Università degli Studi di Salerno, 84084 Fisciano (SA), Italy; # Departamento de Farmacognosia y Medicamentos Organicos, Universidad de los Andes, Mérida, 5101, Venezuela; □ CISUP, Centro per l’Integrazione della Strumentazione Scientifica, Università di Pisa, 56126 Pisa, Italy

## Abstract

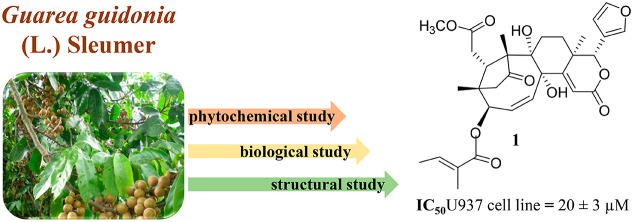

Nine new limonoids (**1**–**9**) were isolated from the stem bark of *Guarea guidonia* (**1**–**4**) and *Cedrela odorata* (**5**–**9**). Their structures were elucidated using 1D and 2D NMR and MS data and chemical methods as three A2,B,D-*seco*-type limonoids (**1**–**3**), a mexicanolide (**4**), three nomilin-type (**5**–**7**) limonoids, and two limonol derivatives (**8** and **9**). A DFT/NMR procedure was used to define the relative configurations of **1** and **3**. A surface plasmon resonance approach was used to screen the Hsp90 binding capability of the limonoids, and the A2,B,D-*seco*-type limonoid 8-hydro-(8S*,9S*)-dihydroxy-14,15-en-chisomicine A, named chisomicine D (**1**), demonstrated the highest affinity. By means of mass spectrometry data, biochemical and cellular assays, and molecular docking, **1** was found as a type of client-selective Hsp90 inhibitor binding to the C-terminus domain of the chaperone.

The Meliaceae, a member of the Sapindales order, is a large family of flowering plants.^1−3^ Its main metabolites are the tetranortriterpenoids, known as limonoids, consisting of compounds with variations of the triterpenoid core structure^4,5^ and which exhibited a wide range of biological activities,^6^ including anticancer and heat-shock protein 90 (Hsp90)-modulating activities.

In the past few years, our research group was devoted to the isolation and chemical characterization of limonoids from different Meliaceae species;^7,8^ in this context, *Guarea guidonia* (L.) Sleumer syn. *Guarea trichilioides* L. and *Cedrela odorata* L. were selected for a phytochemical study. *G. guidonia*, popularly known as “Canjarana”, is a Brazilian species, distributed in the tropical and subtropical forests of South America. It is an evergreen tree that can reach heights between 25 and 30 m, having a straight trunk of over 90 cm in diameter, and characterized as exhibiting white flowers with oblong petals.^9^ The plant seeds macerated with alcohol beverages are used in Brazil as a traditional remedy against rheumatic arthritis,^10^ while the stem bark is used as an abortive and antipyretic.^11^ The oil obtained from the plant bark is used against gonorrhea in South America. Different extracts of the plant are also used in the traditional folk medicine of Venezuela, as a vermicide and for the treatment of cancer.^12^*C. odorata* is a semideciduous tree native to tropical regions of America, also introduced as a cultivated species in Africa and many tropical countries of Asia and Oceania.^13,14^ It is considered a monoecious species that can reach heights of up to 40 m. The infusion of *C. odorata* stem bark is used in South American folk medicine for the treatment of fever, hemorrhage, inflammation, and digestive diseases, including diarrhea, vomiting, and indigestion, while the decoction of the bark in Africa is used as a remedy for malaria and fever.^14^

The molecular chaperone, Hsp90, is a member of a class of evolutionarily conserved molecular chaperones. This protein modulates the stability and activation of several client proteins and has a crucial role in the maintenance of protein homeostasis within cells. Hsp90 clients are involved in angiogenesis and glucose metabolism, growth factor independence, cell cycle progression, tissue invasion and metastases, avoidance of apoptosis, and acquired drug resistance. This chaperone facilitates tumor progression and resistance to therapies; therefore its inhibition could produce the degradation of these abnormal proteins resulting in tumor cell death.^15^

In this paper, the isolation and structural characterization by 1D and 2D NMR and MS data and chemical methods of nine new compounds comprising three A2,B,D-*seco*-type limonoids (**1**–**3**), a mexicanolide (**4**) derivative from *G. guidonia*, three nomilin-type limonoids (**5**–**7**), and two limonol derivatives (**8** and **9**) from *C. odorata* stem bark are reported. Nine known limonoids from *C. odorata*, i.e., four nomilins (**10**–**12** and 7α-acetyldihydronomilin), three mexicanolides (**13**, **14**, and swietenolide), a delevoyin (delevoyin D), and a limonol, 7α,11β-diacetoxylimonol, were also characterized. Compounds **1**–**14** were preliminarily screened by surface plasmon resonance (SPR) experiments followed by biochemical and cellular assays on the most active compound, chisomicine D (**1**), 8-hydro-(8S*,9S*)-dihydroxy-14,15-en-chisomicine A, to establish their Hsp90 modulator activity.

## Results and Discussion

The stem barks of *G. guidonia* and *C. odorata* were extracted with solvents of increasing polarity. The CHCl_3_ and the CHCl_3_–MeOH extracts, subjected to different chromatographies, yielded nine new (**1**–**9**) and nine known compounds.

**Figure 1:**
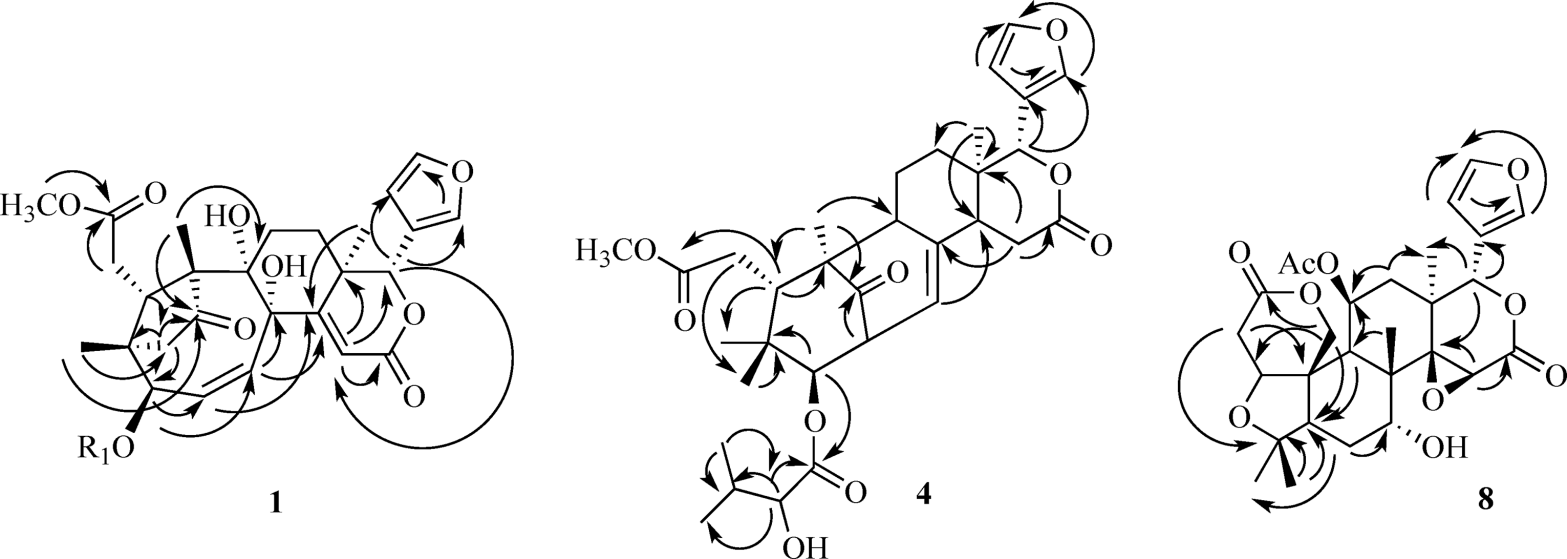
Key HMBC correlations of compounds **1**, **4**, and **8**.

Compound **1** was obtained as a white amorphous powder and showed a molecular formula of C_32_H_38_O_10_, as determined by the HR-ESIMS sodium adduct ion at *m*/*z* 605.2356 [M + Na]^+^. Its fragmentation pattern was characterized by peaks at *m*/*z* 505 [M + Na – 100]^+^ and 461 [M + Na – 100 −44]^+^, due to the subsequent loss of a tiglic acid and a CO_2_ molecule, respectively. The NMR data of **1** (Tables 1 and 2) displayed 32 carbon resonances assignable to four methyls (one methoxy), four methylenes, nine methines (including two oxygenated and six olefinic), five quaternary carbons, two oxygenated tertiary carbons, a carbonyl, a lactone, and an ester group, together with signals for a tigloyl substituent. There were 14 hydrogen deficiencies evident, of which nine were represented by a lactone and two ester carbonyls, a carbonyl group, and five double bonds; therefore, the molecule was pentacyclic. 1D-TOCSY and COSY experiments permitted establishment of the following spin systems: H-3–H-30 for the rearranged ring B, H-5–H_2_-6 and H_2_-11–H_2_-12 for ring C, and H-22–H-23 for ring E. Analyses of 1D and 2D NMR data revealed that **1** was a rearranged A2,B,D-*seco*-type limonoid with a structure similar to chisomicine A,^16^ with the difference being the presence of a Δ^14(15)^ double bond in **1** instead of the Δ^8(14)^ olefinic bond in chisomicine A, as well as the presence in **1** of two hydroxy groups, placed at C-8 and C-9, respectively. The Δ^14(15)^ double bond in **1** was established on the basis of the HMBC correlations (Figure 1) observed between δ 5.76/38.1 (H-15/C-13), 5.76/166.0 (H-15/C-16), 5.76/79.1 (H-15/C-17), and 6.03/119.0 (H-17/C-15) and between δ 6.02/165.8 (H-2/C-14), 1.36/165.8 (Me-18/C-14), and 5.96/165.8 (H-30/C-14), which displayed proton and carbon resonances consistent with the presence of methine and quaternary olefinic carbons at C-15 and C-14, respectively. In particular, the HMBC correlation between H-15–C-16 confirmed that the Δ^14(15)^ double bond was part of an α,β-unsatured δ-lactone D-ring. The 2D NMR spectra also assisted in the assignment of most of the substituents. The proton signal at δ 5.96 (d, *J* = 15.0 Hz, H-30), showing HMBC correlation with the carbon signal at δ_C_ 77.6 (C-8), located a hydroxy group at C-8. The hydroxy group at C-9 was indicated by the HMBC correlation between H-11b–C-9 and Me-19–C-9. The presence of a 4,29,1-bridge, indicating a quite rare cyclopentanone ring A1 as a structural feature of **1**, was confirmed by the HMBC correlations between H_2_-29–C-1, H_2_-29–C-3, H_2_-29–C-4, and H_2_-29–C-10 and between H-5–C-29, Me-28–C-29, Me-19–C-1, and Me-28–C-1, while the presence of a Δ^2(30)^ double bond was deduced by the HMBC correlations H-3–C-2, H-3–C-30, and H-30–C-2. Two HMBC cross-peaks observed between MeO-7–C-7 and H-6a–C-7 enabled a methoxycarbonyl group to be placed at C-6. The signals at δ_H_ 7.41 (q, *J* = 8.0 Hz, H-3′), 1.75 (s, H-5′), and 1.72 (d, *J* = 7.0 Hz, H-4′) and δ_C_ 168.2 (C-1′), 141.4 (C-3′), 129.1 (C-2′), 13.1 (C-4′), and 11.0 (C-5′) were assigned to an *O*-tigloyl substituent at C-3. ROESY correlations were observed between Me-19 and Me-28 and H-3 and H_2_-6. The relative configuration of **1** was determined by comparison of the chemical shifts and coupling constants of the protons of the stereogenic centers with those of chisomicine A and computational methods. The relative configurations of C-8 and C-9 were resolved by using quantum mechanical (QM) methods predicting the ^13^C and ^1^H NMR chemical shifts (DFT/NMR), a methodology developed and optimized by our group for the configurational assignment of natural compounds.^17,18^ This computational procedure consists of four fundamental steps: (a) conformational search and preliminary geometry optimization of all the significantly populated conformers of all possible diastereoisomers of the compound under examination (**1a**–**1d**, Figure S1, Supporting Information); (b) geometry optimization of all the species at the QM level; (c) QM calculations of ^13^C/^1^H NMR chemical shifts of all the structures at the QM level; (d) comparison between experimental and calculated Boltzmann-averaged experimental data (^13^C/^1^H NMR chemical shifts) for all possible diastereoisomers. During the comparative computational/experimental analysis, the mean absolute error, MAE (MAE = ∑(Δδ)/*n*), namely, the summation (∑) of the *n* computed absolute δ error values (Δδ), normalized to the number of Δδ errors considered (*n*)), was used as a statistical parameter, to find the best-fitting model. In the first step, by using Monte Carlo Molecular Mechanics (MCMM) and Molecular Dynamics (MD) simulations, an extensive conformational search at the empirical level was performed for each of the four stereoisomers (**1a** (8R*, 9R*), **1b** (8R*, 9S*), **1c** (8S*, 9R*), and **1d** (8S*, 9S*), Figure S1, Supporting Information), using the OPLS force field (MacroModel software).^19^ In step 2, the MM-derived conformers are geometry optimized by means of QM methods using the MPW1PW91 functional and 6-31G(d) basis set and using the integral equation formalism version of the polarizable continuum model (IEFPCM) for simulating MeOH^20^ as solvent (Gaussian 09 software package).^21^ In this way, refined geometries are generated and evaluated for the subsequent steps. For each QM-derived conformer, NMR-specific parameters, namely, ^13^C/^1^H chemical shifts, are predicted in the third step by GIAO (gauge including atomic orbital) calculations at the QM level using the same functional and the 6-31G(d,p) basis set (Gaussian 09 software package).^21^ The contribution of each conformer to the final Boltzmann ensemble is predicted according to the relative energy computed at the QM level. In this way, the final set of Boltzmann-weighted averages of the specific accounted NMR parameters is computed in the fourth step. The final sets of predicted and experimental data are then compared by using specific statistical parameters (e.g., MAE). As reported above, the Δδ and the MAE parameters were used to characterize unknown stereostructures. Considering the MAE value to compare calculated and experimental ^1^H and ^13^C NMR chemical shifts, **1d** displayed the lowest MAE values (^13^C MAE = 2.04 ppm, ^1^H MAE = 0.23 ppm) versus **1a** (^13^C MAE = 2.70 ppm, ^1^H MAE = 0.46 ppm), **1b** (^13^C MAE = 2.70 ppm, ^1^H MAE = 0.47 ppm), and **1c** (^13^C MAE = 3.29 ppm, ^1^H MAE = 0.49 ppm), suggesting the relative configuration (S*, S*) at C-8 and C-9, and as a consequence, the diastereoisomer **1d** was proposed as the correct structure for **1**. Consequently, the structure of chisomicine D (**1**) was assigned as 8-hydro-(8S*,9S*)-dihydroxy-14,15-en-chisomicine A.

**Table 1:** ^1^H NMR Data of Compounds **1**–**4**^a^

position	**1**	**2**	**3**	**4**
2	6.02^b^	6.05 d (15.0)	6.06 dd (12.0, 8.0)	3.46 dd (18.0, 6.8)
3	4.80 d (9.0)	4.83 d (9.0)	4.88^b^	4.93 d (10.0)
5	3.31^b^	3.31^b^	3.88 br d (13.0)	3.44 d (10.0)
6a	2.84 d (7.7)	2.86 d (7.8)	2.73 dd (16.5, 13.0)	2.53 br d (17.7)
6b			2.65 dd (16.5, 1.5)	2.46 m
9				3.30^b^
11a	1.65 dt (15.0, 3.0)	1.62 dt (15.0, 4.0)	1.62 dt (15.0, 4.1)	2.13^b^
11b	1.20^b^	1.16^b^	1.13^b^	1.77 m
12a	2.02 m	2.03 m	2.27 m	1.63 m
12b	1.92 dt (16.0, 3.7)	1.93 dd (13.5, 7.6)	1.67^b^	1.47 ddd (17.0, 13.0, 6.0)
15a	5.76 s	5.81 s	3.01 br s	3.07 dd (18.0, 7.2)
15b				2.83 br d (18.0)
17	6.03^b^	6.07 s	5.54 s	5.73 s
18	1.36 s	1.34 s	1.17 s	1.11 s
19	1.36 s	1.36 s	1.23 s	1.19 s
21	7.60 br s	7.54 br s	7.67 br s	7.88 br s
22	6.52 br s	6.52 br s	6.57 br s	6.56 br s
23	7.56 br s	7.53 br s	7.56 br s	7.54 br s
28	1.14 s	1.18 s	1.18 s	0.83 s
29	2.18 s	2.22 s	2.19 s	0.85 s
30	5.96 d (15.0)	5.97 d (15.0)	6.20 d (12.0)	5.39 br d (7.8)
MeO-7	3.77 s	3.80 s	3.78 s	3.75 s
2′				4.16 d (3.2)
3a′	7.41 q (8.0)	6.54 br s	7.46 q (8.0)	2.12^b^
3b′		5.68 br s		
4′	1.72 d (7.0)	1.90 s	1.73 d (8.3)	0.85 d (7.5)
5′	1.75 s		1.75 s	0.89 d (7.5)

a Spectra were recorded in methanol-*d*_4_ at 600 MHz. *J* values are in parentheses and reported in Hz; chemical shifts are given in ppm; assignments were confirmed by 1D-TOCSY, COSY, HSQC, and HMBC experiments.

b Overlapped signal.

**Table 2:** ^13^C and ^13^C DEPT NMR Data of Compounds **1**–**4**^a^

	**1**	**2**	**3**	**4**
position	δ_C_, type	δ_C_, type	δ_C_, type	δ_C_, type
1	226.9, C	225.6, C	227.3, C	218.0, C
2	128.0, CH	130.0, CH	139.6, CH	51.0, CH
3	76.8, CH	78.1, CH	80.3, CH	78.1, CH
4	44.0, C	43.6, C	45.5, C	39.1, C
5	40.0, CH	41.1, CH	43.6, CH	41.2, CH
6	32.9, CH_2_	34.3, CH_2_	34.4, CH_2_	32.0, CH_2_
7	175.9, C	174.3, C	176.4, C	175.0, C
8	77.6, C	76.3, C	134.4, C	140.4, C
9	87.3, C	86.6, C	83.2, C	56.3, CH
10	55.5, C	54.2, C	56.4, C	50.0, C
11	28.1, CH_2_	29.6, CH_2_	29.7, CH_2_	20.0, CH_2_
12	26.3, CH_2_	26.2, CH_2_	29.7, CH_2_	33.8, CH_2_
13	38.1, C	38.7, C	39.4, C	41.0, C
14	165.8, C	165.8, C	136.4, C	44.4, CH
15	119.0, CH	119.8, CH	34.3, CH_2_	29.2, CH_2_
16	166.0, C	165.3, C	172.1, C	172.0, C
17	79.1, CH	79.4, CH	81.6, CH	77.1, CH
18	20.0, CH_3_	21.0, CH_3_	16.6, CH_3_	20.5, CH_3_
19	26.0, CH_3_	21.1, CH_3_	19.3, CH_3_	14.5, CH_3_
20	121.0, C	120.3, C	121.0, C	121.4, C
21	140.8, CH	142.6, CH	143.8, CH	141.9, CH
22	109.0, CH	110.0, CH	110.4, CH	110.0, CH
23	142.5, CH	142.2, CH	145.0, CH	141.9, CH
28	22.6, CH_3_	21.4, CH_3_	22.8, CH_3_	21.0, CH_3_
29	45.1, CH_2_	47.0, CH_2_	47.2, CH_2_	18.0, CH_3_
30	145.3, CH	145.7, CH	135.0, CH	121.7, CH
MeO-7	51.2, CH_3_	51.7, CH_3_	52.4, CH_3_	50.8, CH_3_
1′	168.2, C	166.5, C	168.8, C	175.0, C
2′	129.1, C	137.0, C	129.0, C	74.2, CH
3′	141.4, CH	128.4, CH_2_	140.5, CH	31.1, CH
4′	13.1, CH_3_	17.8, CH_3_	14.5, CH_3_	15.0, CH_3_
5′	11.0, CH_3_		12.4, CH_3_	18.0, CH_3_

a Spectra were recorded in methanol-*d*_4_ at 150 MHz. Chemical shifts are given in ppm; assignments were confirmed by HSQC and HMBC experiments.

Compound **2**, obtained as a white amorphous powder, showed a molecular formula of C_31_H_36_O_10_, as established by the HR-ESIMS spectrum (*m*/*z* 591.2202 [M + Na]^+^). The NMR data of **2** (Tables 1 and 2) indicated its structure to be closely related to that of **1**, with the only difference being the presence of an *O*-methacryloyl moiety at C-3 in **2** replacing the *O*-tigloyl substituent in **1**. The presence of this different carboxylic side chain in **2** [(δ_H_ 6.54 (br s, H-3′a), 5.68 (br s, H-3′b), and 1.90 (s, Me-4′); δ_C_ 166.5 (C-1′), 137.0 (C-2′), 128.4 (C-3′), and 17.8 (C-4′)] was confirmed by HMBC correlations between H-3′–C-1′ and H-3′–C-4′ and between Me-4′–C-1′, Me-4′–C-3′, and H-3–C-1′. A β-orientation of the tigloyl substituent was deduced from the ROE correlations observed between H-3–H_2_-29 and H-3–H_2_-6. Therefore, the structure of chisomicine E (**2**) was established as shown.

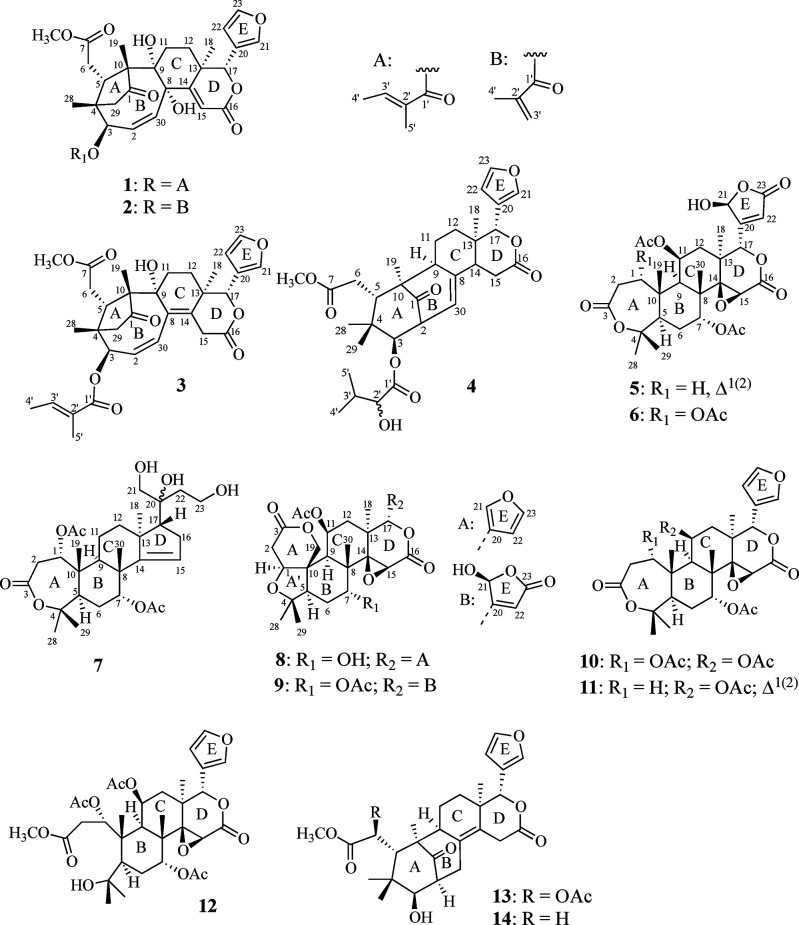



Compound **3** showed a sodiated molecular ion at *m*/*z* 589.2412 [M + Na]^+^ corresponding to the molecular formula C_32_H_38_O_9_. Other fragmentation peaks were observed at 489.1882 [M + Na – 100]^+^ and 445.1983 [M + Na – 100 – 44]^+^. The NMR spectroscopic data (Tables 1 and 2) suggested **3** was a rearranged A2,B,D-*seco*-type limonoid structurally similar to **1**, but with different substitution patterns at rings C and D. Analyses of the 2D NMR spectra of **3** indicated its structure to be closely related to that of chisomicine A, with the only difference being the presence of a hydroxy group at C-9 in **3**, replacing the methine proton at C-9 in chisomicine A. The position of this hydroxy group was deduced by the HMBC cross-peaks observed between δ 1.62 (H-11a) and 83.2 (C-9) and δ 2.27 (H-12a) and 83.2 (C-9), which suggested C-9 was an oxygenated tertiary carbon. The relative configuration at C-9 was elucidated by using the aforementioned QM methods. The two possible diastereoisomers (**3a** (9R*) and **3b** (9S*), Figure S1, Supporting Information) were subjected to the computational protocol. After optimization of the geometries, the conformers were inspected in order to avoid further possible redundant conformers. The NMR chemical shift data were computed for all the possible diastereoisomers featuring a specific relative configuration at C-9 (**3a** and **3b**) at the MPW1PW91/6-31G(d,p) functional/basis set in MeOH (IEFPCM).^20^ For each of these, the weighted averages of the predicted ^13^C and ^1^H NMR chemical shifts were computed at the density functional level of theory, accounting for the energies of the sampled conformers on the final Boltzmann distribution, using TMS as a reference compound. For each atom of the investigated molecules, experimental and calculated ^13^C and ^1^H NMR chemical shifts were compared, and afterward, the MAEs for two of the possible diastereoisomers were computed. The results highlighted the different pattern of accordance between ^13^C/^1^H MAEs for each possible diastereoisomer (**3a** and **3b**) and the different experimental data of **3**, indicating that the most probable diastereoisomer was **3a** (9R*), showing the best-related ranking (^13^C MAE = 1.29 ppm, ^1^H MAE = 0.24 ppm) versus **3b** (^13^C MAE = 2.16 ppm, ^1^H MAE = 0.32 ppm). Thus, the chisomicine F (**3**) structure was assigned as (9R*)-hydroxychisomicine A.

This is only the second example of a naturally occurring rearranged A2,B,D-*seco*-type limonoid similar to chisomicine A.^16^ Thus, the cyclopentanone A1-ring in compounds **1**, **2**, and **3** represents a quite rare structural characteristic of these compounds. Najmuldeen et al. suggested that this type of tetranortriterpenoid could be the result of the A2-ring demolition in a phragmalin skeleton.^16^

Compound **4̧**, a white amorphous powder, had a molecular formula of C_32_H_42_O_9_ as deduced from the HR-ESIMS molecular ion [M + Na]^+^ at *m*/*z* 593.2714, indicating 12 hydrogen deficiencies. The 1D and 2D NMR spectroscopic data (Tables 1 and 2) of **4** showed that this molecule was pentacyclic, displaying 32 carbon resonances assignable to five methyls (one methoxy), four methylenes, 10 methines (including four olefinic and two oxygenated), a lactone, an ester, a carbonyl functions, and five quaternary carbons, along with signals for a 2′-hydroxyisovaleryl substituent. Analysis of the 2D NMR data suggested that **4** was a mexicanolide-type limonoid^22^ with the same structure as khasenegasin I,^23^ with the only difference being the presence of a 2′-hydroxyisovaleroyloxy substituent at C-3 in **4**. The presence of this side chain was characterized by the ^1^H NMR signals at δ 4.16 (d, *J* = 3.2 Hz, H-2′), 2.12 (overlapped signal, H-3), 0.89 (d, *J* = 7.5 Hz, H_3_-5′), and 0.85 (d, *J* = 7.5 Hz, H_3_-4′), which correlated in the HSQC spectrum with δ 74.2 (C-2′), 31.1 (C-3′), 18.0 (C-5′), and 15.0 (C-4′), and confirmed by the HMBC correlations (Figure 1) between H-2′–C-1′, H-2′–C-3′, H-2′–Me-4′, H-2′–Me-5′, between Me-5′–C-2′, Me-5′–C-3′, and between H-3–C-1′, establishing the location of the 2′-hydroxyisovaleroyloxy group at C-3. The configuration of all stereocenters of **4** was determined to be the same as in khasenegasin I and similar mexicanolides^23,24^ by analysis of ^1^H NMR and 1D-ROESY data (correlations between H-3–Me-19 and H-5–Me-29). Thus, the structure 3-(2′-hydroxyisovaleroyl)khasenegasin I was established for **4**. From a literature survey, the presence of side chains similar to those exhibited by compounds **1**–**4**, such as tigloyloxy and 2′-hydroxyisovaleroyloxy groups, seems to be a usual characteristic of limonoids isolated from plants of the Melioideae subfamily, including species belonging to *Guarea*.^5^

Compound **5**, obtained as a white amorphous powder, showed a molecular formula of C_30_H_36_O_12_, as determined by the HR-ESIMS ion at *m*/*z* 611.2108 [M + Na]^+^. The NMR data (Tables 3 and 4) displayed 30 carbon resonances assignable to five methyls, two methylenes, 10 methines (including five oxygenated and three olefinic), four quaternary carbons, two oxygenated tertiary carbons, and three lactone groups, together with signals for two acetyl groups. There were 13 hydrogen deficiencies evident, of which seven were represented by five ester carbonyls and two double bonds; therefore, the molecule was hexacyclic. 1D-TOCSY and COSY data revealed the spin systems H-1–H-2, H-5–H-7, and H-9–H_2_-12. Analysis of the 2D NMR spectra, especially the HMBC data, confirmed that **5** was a rearranged nomilin/obacunol-type limonoid with a structure similar to 7-deoxo-7α-acetoxykihadanin A,^25^ with the only difference being the presence of an additional acetoxy group at C-11. The position of this acetoxy substituent was suggested by the HMBC cross-peak observed between δ 1.32/68.5 (Me-18/C-11) and the COSY correlations between H-9–H-11 and H-11–H_2_-12, which exhibited proton and carbon resonances for C-11 consistent with the presence of an acetoxy group. The ROESY correlations between H-5–Me-28, H-9–Me-28, H-5–H-11, and H-11–H-15 showed that these protons were cofacial; in the same way, the correlation between H-7–Me-30 indicated that these protons were also cofacial. The relative configuration of **5** was also confirmed by comparing the chemical shifts and coupling constants of protons attached to stereogenic centers with those of 7-deoxo-7α-acetoxykihadanin A and similar compounds.^14,25^ Consequently, the structure of **5** was defined as 7-deoxo-7α,11β-diacetoxykihadanin A.

**Table 3:** ^1^H NMR Data of Compounds **5**–**9**^a^

position	**5**	**6**	**7**	**8**	**9**
1	6.69 d (12.6)	4.89^b^	4.86^b^	4.28 d (2.0)	4.38 d (3.0)
2a	5.97 d (12.6)	3.50 br d (16.0)	3.47 br d (15.8)	2.81 dd (17.0, 3.3)	2.85 dd (17.0, 3.7)
2b		3.05 dd (16.0, 7.5)	3.05 dd (7.4, 15.8)	2.49 br d (17.5)	2.53 br d (17.0)
5	2.57^b^	2.45^b^	2.52 m	2.70 br d (12.5)	2.38 dd (15.0, 2.5)
6a	2.17 m	2.13^b^	2.09^b^	1.68 m	1.87 br dd (14.0, 3.3)
6b	2.07^b^	2.06^b^	1.98^b^	2.03 m	2.13^b^
7	4.70^b^	4.64 br d (2.5)	5.17 br s	3.56 br s	4.70 br s
9	2.55^b^	2.92 d (3.5)	2.63 m	2.77 d (3.3)	2.80 br d (3.7)
11a	5.78 m	5.28 br dd (9.0, 4.3)	1.62^b^	5.43 dd (9.0, 4.0)	5.53 m
11b			1.49 m		
12a	2.60^b^	2.46^b^	2.03^b^	2.28 dd (14.3, 10.0)	1.62 br d (14.0)
12b	1.61^b^	1.65 dd (15.0, 5.5)	1.61^b^	1.59 dd (15.6, 4.4)	
15	3.73 s	3.70 s	5.33 br s	3.88 s	3.70 s
16a			2.50 m		
16b			2.11^b^		
17	5.49 br s	5.52 br s	1.97^b^	5.09 s	5.52 br s
18	1.32 s	1.40 s	1.27 s	1.33 s	1.34 s
19a	1.57 s	1.51 s	1.25 s	5.09 d (13.0)	5.06 d (13.5)
19b				4.72 d (13.0)	4.77 d (13.5)
21	6.15 br s	–c	3.63 br d (3.0)	7.54^b^	–c
22a	6.29 br s	–c	1.93^b^	6.46 s	–c
22b			1.77 m		
23			3.72 m	7.54^b^	
28	1.37 s	1.39 s	1.40 s	1.28 s	1.30 s
29	1.50 s	1.58 s	1.58 s	1.12 s	1.13 s
30	1.40 s	1.30 s	1.24 s	1.08 s	1.19 s
AcO-1		2.12 s	2.03 s		2.10 s
AcO-7	2.08 s	2.11 s	2.09 s		
AcO-11	2.01 s	2.11 s		2.11 s	2.10 s

a Spectra were recorded in methanol-*d*_4_ at 600 MHz. *J* values are in parentheses and reported in Hz; chemical shifts are given in ppm; assignments were confirmed by 1D-TOCSY, COSY, HSQC, and HMBC experiments.

b Overlapped signal.

c Signal cannot be observed clearly from 1D and 2D NMR. Weak signals are due presumably to an instable hemiacetal function and tautomerism of the butenolide ring in solution.

**Table 4:** ^13^C and ^13^C DEPT NMR Data of Compounds **5**–**9**^a^

	**5**	**6**	**7**	**8**	**9**
position	δ_C_, type	δ_C_, type	δ_C_, type	δ_C_, type	δ_C_, type
1	154.5, CH	72.4, CH	72.4, CH	79.7, CH	79.4, CH
2	120.4, CH	35.3, CH_2_	35.6, CH_2_	35.2, CH_2_	35.0, CH_2_
3	167.7, C	171.6, C	171.0, C	172.1, C	172.0, C
4	85.0, C	86.6, C	86.2, C	81.6, C	81.0, C
5	45.9, CH	45.6, CH	45.2, CH	53.2, CH	55.0, CH
6	28.4, CH_2_	26.5, CH_2_	27.0, CH_2_	28.1, CH_2_	25.0, CH_2_
7	75.7, CH	75.3, CH	75.6, CH	71.6, CH_2_	75.4, CH
8	41.0, C	42.8, C	42.3, C	43.8, C	43.5, C
9	50.0, CH	40.5, CH	36.3, CH	45.0, CH	45.9, CH
10	44.0, C	46.4, C	44.0, C	45.2, C	46.0, C
11	68.5, CH	67.5, CH	17.3, CH_2_	70.0, CH	69.1, CH
12	37.7, CH_2_	37.0, CH_2_	35.4, CH_2_	37.6, CH_2_	37.4, CH_2_
13	38.7, C	40.7, C	47.0, C	37.8, C	37.9, C
14	68.1, C	69.0, C	158.7, C	68.6, C	68.2, C
15	53.0, CH	54.2, CH	119.5, CH	55.0, CH	52.0, CH
16	166.0, C	166.8, C	30.7, CH_2_	168.8, C	166.0, C
17	79.5, CH	77.0, CH	59.6, CH	79.2, CH	78.5, CH
18	19.0, CH_3_	19.0, CH_3_	21.0, CH_3_	17.7, CH_3_	18.0, CH_3_
19	19.0, CH_3_	15.9, CH_3_	15.0, CH_3_	68.7, CH_2_	68.3, CH_2_
20	–b	–b	69.3, C	121.2, C	–b
21	99.8, CH	–b	66.3, CH_2_	143.5, CH	–b
22	123.7, CH	–b	40.5, CH_2_	110.4, CH	–b
23	168.5, C	–b	58.6, CH_2_	143.0, CH	–b
28	31.9, CH_3_	34.0, CH_3_	34.4, CH_3_	30.0, CH_3_	30.0, CH_3_
29	25.1, CH_3_	22.5, CH_3_	23.2, CH_3_	21.0, CH_3_	20.0, CH_3_
30	20.6, CH_3_	20.0, CH_3_	27.5, CH_3_	20.0, CH_3_	20.5, CH_3_
COCH_3_-1		21.0, CH_3_	20.8, CH_3_		
COCH_3_-1		171.0, C	171.0, C		
COCH_3_-7	21.0, CH_3_	20.6, CH_3_	20.9, CH_3_		20.1, CH_3_
COCH_3_-7	169.8, C	170.0, C	169.8, C		172.0, C
COCH_3_-11	21.0, CH_3_	20.6, CH_3_		22.0, CH3	20.1, CH_3_
COCH_3_-11	169.7, C	171.0, C		170.0, C	172.0, C

a Spectra were recorded in methanol-*d*_4_ at 150 MHz. Chemical shifts are given in ppm; assignments were confirmed by HSQC and HMBC experiments.

b Signal cannot be observed clearly from 1D and 2D NMR. Weak signals are due presumably to an instable hemiacetal function and tautomerism of the butenolide ring in solution.

Compound **6**, obtained as a white amorphous powder, showed a molecular formula of C_32_H_40_O_14_, as established by the HR-ESIMS spectrum (*m*/*z* 671.2309 [M + Na]^+^). Its fragmentation pattern was characterized by ions at *m*/*z* 611.2089 [M + Na – 60]^+^ and 567.2194 [M + Na – 60 – 44]^+^, due to the sequential loss of an HOAc and a CO_2_ molecule, respectively. The NMR data of **6** (Tables 3–5) indicated its structure to be closely related to that of **5**, with the difference being the absence in **6** of the Δ^1(2)^ double bond in **5** and the presence in **6** of an acetoxy substituent at C-1. 1D-TOCSY and COSY correlations between H-1/H_2_-2 evidenced the presence of an oxygenated methine at C-1 and a geminal coupled methylene at C-2, thus indicating the absence of the unsaturation between these carbons. Therefore, the signals at δ 4.89 (overlapped signal, H-1), 3.50 (br d, *J* = 16.0 Hz, H-2a), and 3.05 (dd, *J* = 16.0, 7.5 Hz, H-2b), which correlated in the HSQC spectrum with δ 72.4 (C-1) and 35.3 (C-2), were assigned to the seven-membered lactone A-ring and confirmed by the HMBC correlations between H-1–C-3 and H-1–C-10 and between H_2_-2–C-3 and H_2_-2–C-10. The configuration of the acetoxy substituent at C-1 and the hydroxy substituent at C-21 was established by comparison with similar compounds.^7,14,26,27^ Therefore, the structure of compound **6** was assigned as 1,2-dihydro-7-deoxo-1α,7α,11β-triacetoxykihadanin A.

**Table 5:** ^1^H, ^13^C, and ^13^C DEPT NMR Data of Compounds **6** and **9**^a^

	**6**		**9**	
position	δ_H_	δ_C_, type	δ_H_	δ_C_, type
1	4.93 br d (2.5)	71.2, CH	4.17 br d (4.5)	80.2, CH
2a	3.59 br d (16.0)	34.6, CH_2_	3.01 dd (17.0, 3.6)	35.5, CH_2_
2b	3.10 dd (16.0, 7.5)		2.40 br d (17.0)	
3		170.9, C		171.0, C
4		84.7, C		82.0, C
5	2.41^b^	44.8, CH	2.25 br t (8.7)	55.8, CH
6a	2.13^b^	26.3, CH_2_	1.94 m	25.3, CH_2_
6b	1.95 ddd (15.0, 7.5, 2.5)			
7	4.56 br d (2.5)	74.6, CH	4.65 br s	74.7, CH
8		41.8, C		45.0, C
9	2.87 d (2.0)	39.9, CH	2.67 d (5.0)	46.9, CH
10		45.4, C		46.0, C
11	5.25 m	68.5, CH	5.38 m	69.4, CH
12a	2.45^b^	38.2, CH_2_	2.55 dd (14.4, 10.0)	39.0, CH_2_
12b	1.62^b^		1.70^b^	
13		41.8, C		40.0, C
14		68.5, C		69.1, C
15	3.70 s	54.1, CH	3.61 s	56.8, CH
16		169.4, C		168.2, C
17	5.51 s	77.0, CH	5.52 br s	78.8, CH
18	1.41 s	18.3, CH_3_	1.36 s	19.4, CH_3_
19a	1.53 s	17.2, CH_3_	5.12 d (14.0)	67.9, CH_2_
19b			4.53 d (14.0)	
20		165.0, C		164.0, C
21	6.14 br s	97.4, CH	6.12 br s	97.8, CH
22	6.32 br s	124.3, CH	6.35 br s	124.6, CH
23		168.4, C		169.3, C
28	1.40 s	34.2, CH_3_	1.39 s	29.6, CH_3_
29	1.60 s	23.6, CH_3_	1.13 s	20.5, CH_3_
30	1.30 s	21.1, CH_3_		21.4, CH_3_
COCH_3_-1	2.15 s	21.6, CH_3_		
COCH_3_-1		169.4, C		
COCH_3_-7	2.15 s	20.2, CH_3_	2.20 s	21.6, CH_3_
COCH_3_-7		169.4, C		169.5, C
COCH_3_-11	2.15 s	21.1, CH_3_	2.15 s	21.4, CH_3_
COCH_3_-11		169.4, C		170.5, C

a Spectra were recorded in CDCl_3_ at 600 MHz (^1^H) and 150 MHz (^13^C). *J* values are in parentheses and reported in Hz; chemical shifts are given in ppm; assignments were confirmed by 1D-TOCSY, COSY, HSQC, and HMBC experiments.

b Overlapped signal.

Compound **7** was obtained as a white amorphous powder. Its molecular formula, C_30_H_46_O_9_, was established from the sodiated molecular ion in the HR-ESIMS data at *m*/*z* 573.3041 [M + Na]^+^, indicating that **7** had 12 indices of hydrogen deficiency. Another fragmentation peak was observed at *m*/*z* 513.2820 [M + Na – 60]^+^. The NMR spectroscopic data (Tables 3 and 4) of **7** indicated that four of the eight indices of hydrogen deficiency arose from two esters, a lactone, and a double bond; therefore, the molecule was tetracyclic. 1D and 2D NMR experiments revealed that **7** had five methyl singlets, eight methylenes (two oxygenated), six methines (one olefinic and two oxygenated), four quaternary carbons, two oxygenated tertiary carbons, and a lactone, together with signals of two acetoxy groups. The NMR data suggested that **7** shared a common structure with cedrelosin D,^7^ with the only difference being the presence of a relatively rare 20,21,23-butanetriol moiety at C-17 in **7** instead of the cyclic side chain of cedrelosin D. This moiety was supported by HSQC cross-peaks between δ 3.63 (H-21) and 66.3 (C-21); δ 1.93 (H-22a), 1.77 (H-22b), and 40.5 (C-22); and δ 3.72 (m, H_2_-23) and 58.6 (C-23) and by the HMBC cross-peaks between H-21–C-17, H-21–C-20, and H-21–C-22. The HMBC correlation of δ 4.86/171.0 (H-1–1-COCH_3_) demonstrated that an acetoxy group was attached to C-1, while the HMBC cross-peak at δ 1.98/75.6 (H-6b/C-7) indicated an oxygenated methine C-7, which must be *O*-acetylated. The relative configuration of **7** was established based on 1D-ROESY data and comparison with the literature.^7,14^ ROESY interactions between δ 5.17/1.58 (H-7/Me-29) indicated the β-orientation of H-7 and the consequential α-orientation of the acetoxy group. H-9 (δ 2.63) correlated with Me-18 (δ 1.27) and Me-28 (δ 1.40), suggesting that Me-18 and Me-28 were cofacial. The chemical shift and coupling constant of the remaining stereogenic center showed that **7** had the same absolute configuration as cedrelosin D and similar compounds.^7,26,28^ Based on the above results, the structure of cedrelosin F (**7**) was elucidated as shown.

Compound **8** was obtained as a white and amorphous powder and had a molecular formula of C_28_H_34_O_10_ as deduced from the molecular sodium adduct [Ma + Na]^+^ at *m*/*z* 553.2038 observed in the HR-ESIMS, indicating that **8** had 12 hydrogen deficiencies. Other fragmentation peaks were observed at *m*/*z* 493.1825 [M + Na – 60]^+^ and 449.1931 [M + Na – 60 – 44]^+^. The NMR spectroscopic data (Tables 3 and 4) showed proton signals assignable to an oxygenated methine at C-1 [δ_H_ 4.28 (d, *J* = 2.0 Hz, H-1)] and an oxygenated geminal coupled methylene at C-19 [δ_H_ 5.09 (d, *J* = 13.0 Hz, H-19a), 4.72 (d, *J* = 13.0 Hz, H-19b)], which correlated with the carbon resonances at δ 79.7 (C-1) and 68.7 (C-19) in the HSQC spectrum, respectively. These signals, together with those displayed by the carbons C-3 (172.1 ppm), C-4 (81.6 ppm), and C-10 (45.2 ppm) in the 2D NMR experiments, were found to be characteristics of a lactone A-ring, involving C-19, similar to that exhibited by limonol derivatives.^29,30^ This assumption was also supported by the HMBC correlations (Figure 1) observed between H-2b–C-4, H-2b–C-10, H_2_-19–C-1, H_2_-19–C-3, and H_2_-19–C-5. A furan E-ring was evidenced by the ^1^H NMR signals at δ_H_ 7.54 (s, H-21 and H-23) and 6.46 (s, H-22) and confirmed by the 2D NMR spectroscopic data. An epoxidized δ-lactone D-ring like that in limonol was also recognized, confirming that **8** shared a common structure with limonol,^7,31^ with the only difference being the presence of an additional acetoxy group. The location of this substituent in **8** was deduced by the HMBC correlations between H-9–C-11 and H-12a–C-11 and by COSY correlations between H-9–H-11, H-11/H-12a, and H-11/H-12b, which suggested an oxygenated methine at C-11, carrying an acetoxy group. The relative configuration of **8** was defined by ROESY experiments: Me-28 showed correlations with H-5 and H-9; Me-30 with H_2_-19; and Me-18 with H-9. The configuration of all the stereocenters of **8** was confirmed to be the same as in other similar limonol derivatives.^7,31^ Thus, the structure of 11β-acetoxylimonol (**8**) was assigned as shown.

Compound **9** showed an HR-ESIMS sodium adduct ion at *m*/*z* 627.2045 [M + Na]^+^, corresponding to the molecular formula C_30_H_36_O_13_. Another fragmentation peak was observed at *m*/*z* 567.1841 [M + Na – 60]^+^. The NMR spectroscopic data (Tables 3–5) suggested **9** was a limonol derivative with rings A, B, C, and D structurally similar to those of **8**. Analyses of the 2D NMR data of **9** indicated its structure to be closely related to that of cedrelosin B,^7^ with the only difference being the presence of an additional *O*-acetyl substituent at C-11 in **9**. The position of this acetoxy group was readily deduced by the HMBC correlation between H-11 (δ_H_ 5.53) and the carbonyl carbon of the acetoxy group [δ_C_ 172.0 (7-COCH_3_)]. Thus, the structure of **9**, named 11β-acetoxycedrelosin B, was defined as shown.

Known compounds were identified as 7α,11β-diacetoxydihydronomilin (**10**),^32^ 11β-acetoxybacunyl acetate (**11**),^14^ odoralide (**12**),^14^ 6-*O*-acetylswietenolide (**13**),^33^ proceranolide (**14**),^33^ delevoyin D,^7^ 7α,11β-limonoldiacetate,^7^ 7α-acetyldihydronomilin,^7,34^ and swietenolide,^33^ using NMR and MS data and comparison with those reported in the literature. Delevoyin D, 7α,11β-limonoldiacetate, 7α-acetyldihydronomilin, and swietenolide were reported by us from *C. odorata* leaves.^7^

Several limonoids have been described for their ability to interact with the molecular chaperone Hsp90, affecting its activities.^7,35,36^ Therefore, the affinity of compounds **1**–**14** toward Hsp90α, by an SPR approach was assayed, using the well-known Hsp90 inhibitor radicicol as a positive control.^8^ The SPR assay involves the measurement of thermodynamic and kinetic parameters of limonoids/Hsp90α complex formation. Compounds **1**, **8**, **11**, and **13** effectively interacted with the protein (Table 6), as inferred by values of the measured *K*_D_s, falling in the 15–30 nM range. Compound **9** showed a low affinity for Hsp90, whereas all the other assayed molecules did not bind to the immobilized chaperone.

**Table 6:** Thermodynamic Constants Measured by Surface Plasmon Resonance for the Interaction between Compounds **1**–**14** and Immobilized Hsp90^a^

compound	*K*_D_ (nM)^a^
**1**	18.2 ± 1.9
**2**	no binding
**3**	no binding
**4**	no binding
**5**	no binding
**6**	no binding
**7**	no binding
**8**	24.8 ± 2.3
**9**	182.8 ± 18.9
**10**	no binding
**11**	25.3 ± 1.8
**12**	no binding
**13**	25.5 ± 3.4
**14**	no binding
radicicol	1.8 ± 0.4

a Results were given as mean ± standard deviation (*n* = 3).

The potential antiproliferative or cytotoxic activity of isolates on human HeLa (epithelial carcinoma) and U937 (human myeloid leukemia) cell lines was therefore studied. The cells were incubated for 48 h with an increasing concentration of limonoids (10–100 μM), and cell viability was determined by the MTT proliferation assay.^37^ Compound **1** showed a growth inhibition toward the U937 cell line (IC_50_ value of 20 ± 3 μM), while it was slightly active on HeLa cells (IC_50_ > 50 μm) and did not show any cytotoxic activity on PBMC (nontumor human peripheral blood mononuclear cell line). All other compounds were inactive on both the tested cancer cell lines.

**Figure 2:**
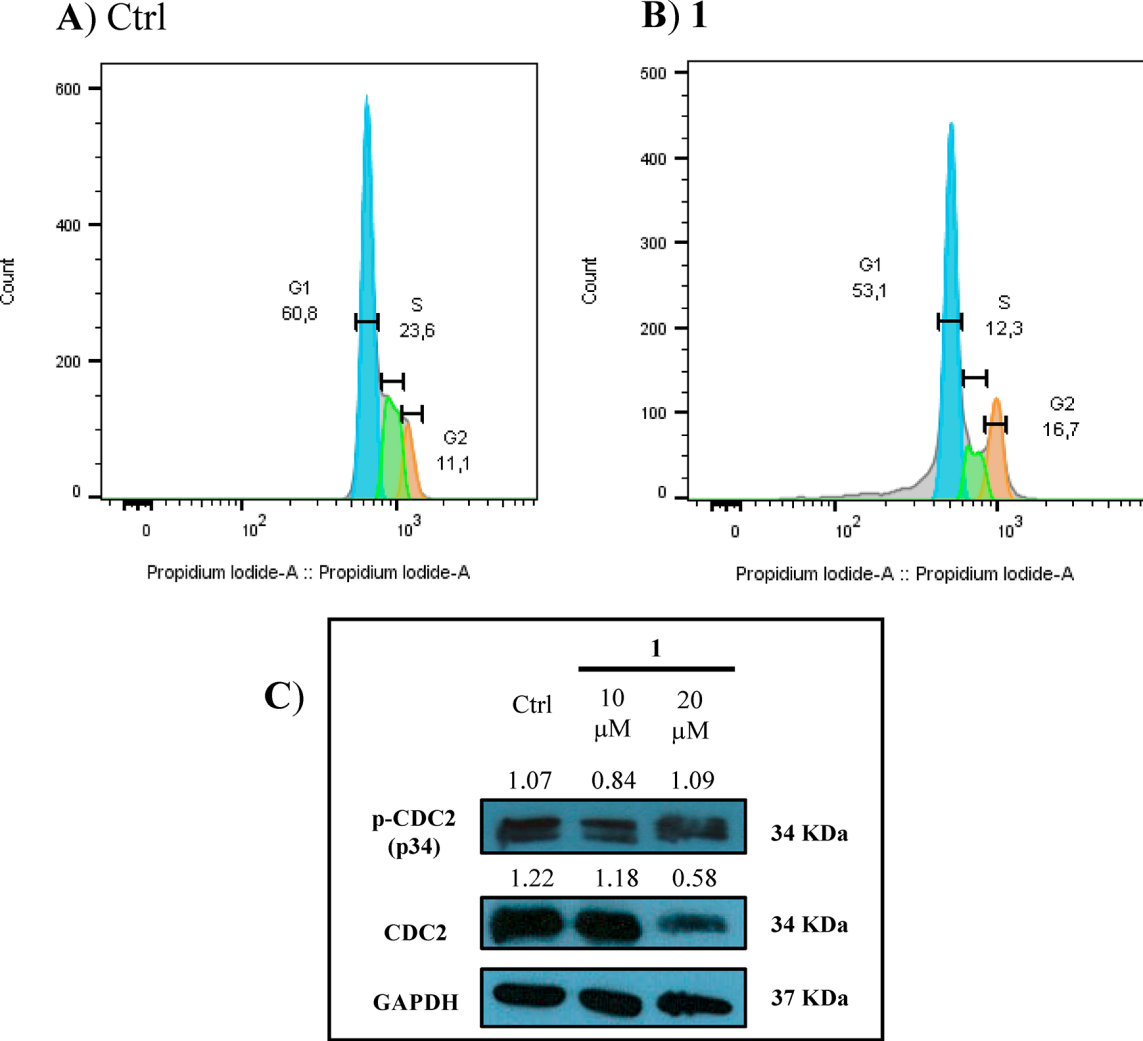
Flow cytometric evaluation of DNA content using propidium iodide. (A) U937 cells treated with vehicle (DMSO) for 48 h. (B) U937 cells treated with 20 μM **1**. (C) Western blot analysis of p-CDC2 (p34) and CDC2 proteins in cells treated with vehicle (DMSO) and **1** (10 and 20 μM). Normalized results of densitometric analysis are reported. The blot is representative of two different experiments providing similar results.

**Figure 3:**
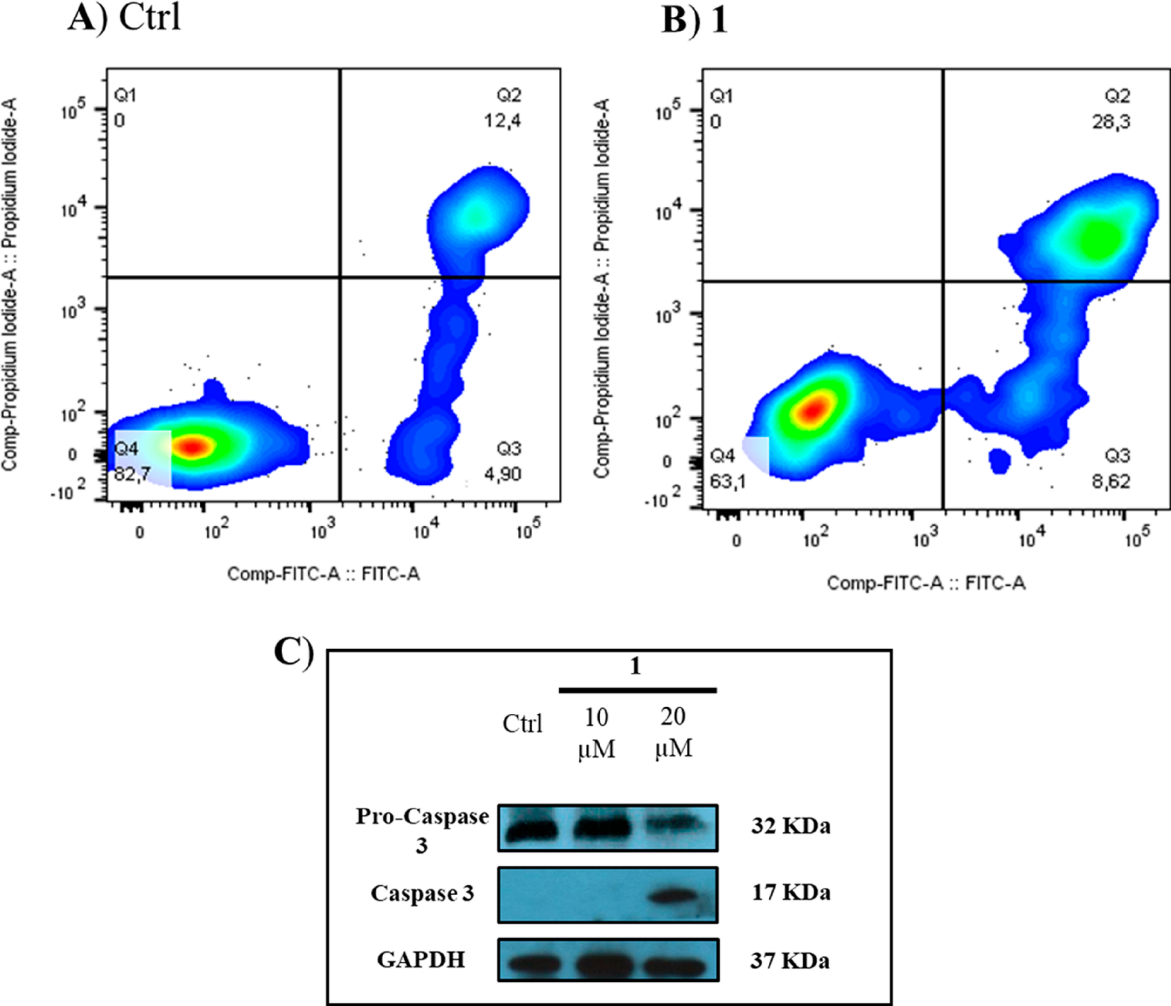
Flow cytometry experiment using the annexin V-FITC/PI protocol. (A) U937 cells treated with vehicle (DMSO) for 48 h. (B) U937 cells treated with 20 μM **1**. (C) Western blot analysis of pro-caspase 3 and caspase 3 in cells treated with vehicle (DMSO) and **1** (10 and 20 μM).

These results prompted us to investigate the mechanism of action of **1** by evaluating its effect on the cell cycle and apoptosis. A significant decrease of the S phase and a slight peak of the G2 phase were detected in U937 cells incubated with 20 μM compound **1** for 48 h; it suggests a G2 phase cell cycle arrest associated with an increase of the p-CDC2/CDC2 ratio induced by cell incubation with compound **1** (Figure 2).^38^ A similar effect was also observed when U937 cells were treated with the positive control radicicol (Figure S47, Supporting Information). Based on this result, apoptosis was evaluated by performing annexin V/FITC analysis, revealing an increase of about 15% of late apoptosis in treated cells, compared to the negative control. Herein, U937 exposure to 20 μM **1** also induced the cleavage of pro-caspase 3 to generate the activity, thus confirming those conditions to stimulate apoptosis (Figure 3).

**Figure 4:**
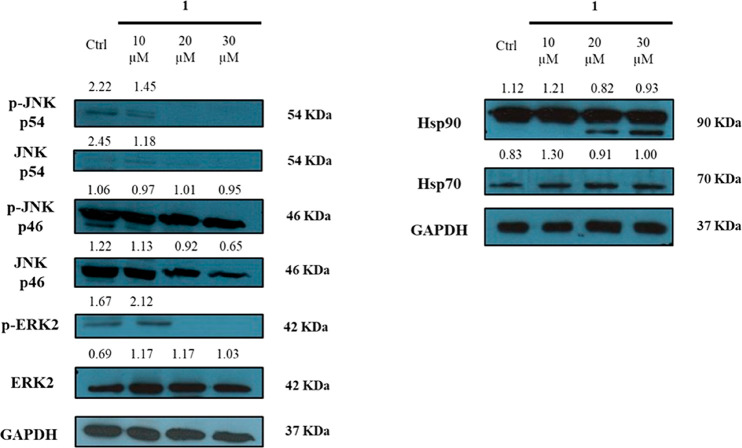
Effects of **1** on Hsp90α, Hsp70, and different Hsp90 client protein levels in U937 cells after treatment with **1** for 48 h. GAPDH was used as loading control. Normalized results of densitometric analysis are reported. The blots are representative of two different experiments providing similar results.

The effect of compound **1** on Hsp90α bioactivity was next investigated. First, the possible inhibition of Hsp90 ATPase activity was assayed; however, incubation of Hsp90α with 10 or 20 μM of compound **1** did not affect its enzymatic efficiency (data not shown). Subsequently, compound **1** was tested for its effect on some Hsp90α client protein levels (Figure 4). U937 cell treatment with **1** (10, 20, and 30 μM) induced a strong depletion of p-ERK2, whereas no significant variation of ERK concentration was observed. The level of both isoforms of JNK was significantly reduced by cell exposure to **1**, regardless of phosphorylation.^39^ Treatment with 30 μM **1** also affected Hsp70 cellular levels, whereas the Hsp90 amount was down-regulated in a dose-dependent manner. Interestingly, following a 48 h incubation with 20 and 30 μM of compound **1**, an Hsp90 cleaved form, with an apparent molecular weight of 70 kDa, was observed. This evidence suggested an Hsp90 cleavage induced by **1**. A similar effect was described for other bioactive compounds, producing Hsp90 truncated species through enzyme-catalyzed or nonenzymatic mechanisms.^40^ In order to further characterize this Hsp90 fragment, an HRMS-based approach was performed, leading to the determination of the protein region depleted following the U937 cell treatment with **1**. The results (Table S1, Supporting Information) demonstrated that the 70 kDa fragment of the chaperone was generated by the elimination of the C-terminus of the protein, as a consequence of proteolytic events occurring in the region between amino acids 565 and 604 of Hsp90. These results confirmed that **1** affected Hsp90 activity, although the mechanism of action appeared to be different from that described for several inhibitors binding at the N-terminal domain of the protein.^40^

**Figure 5:**
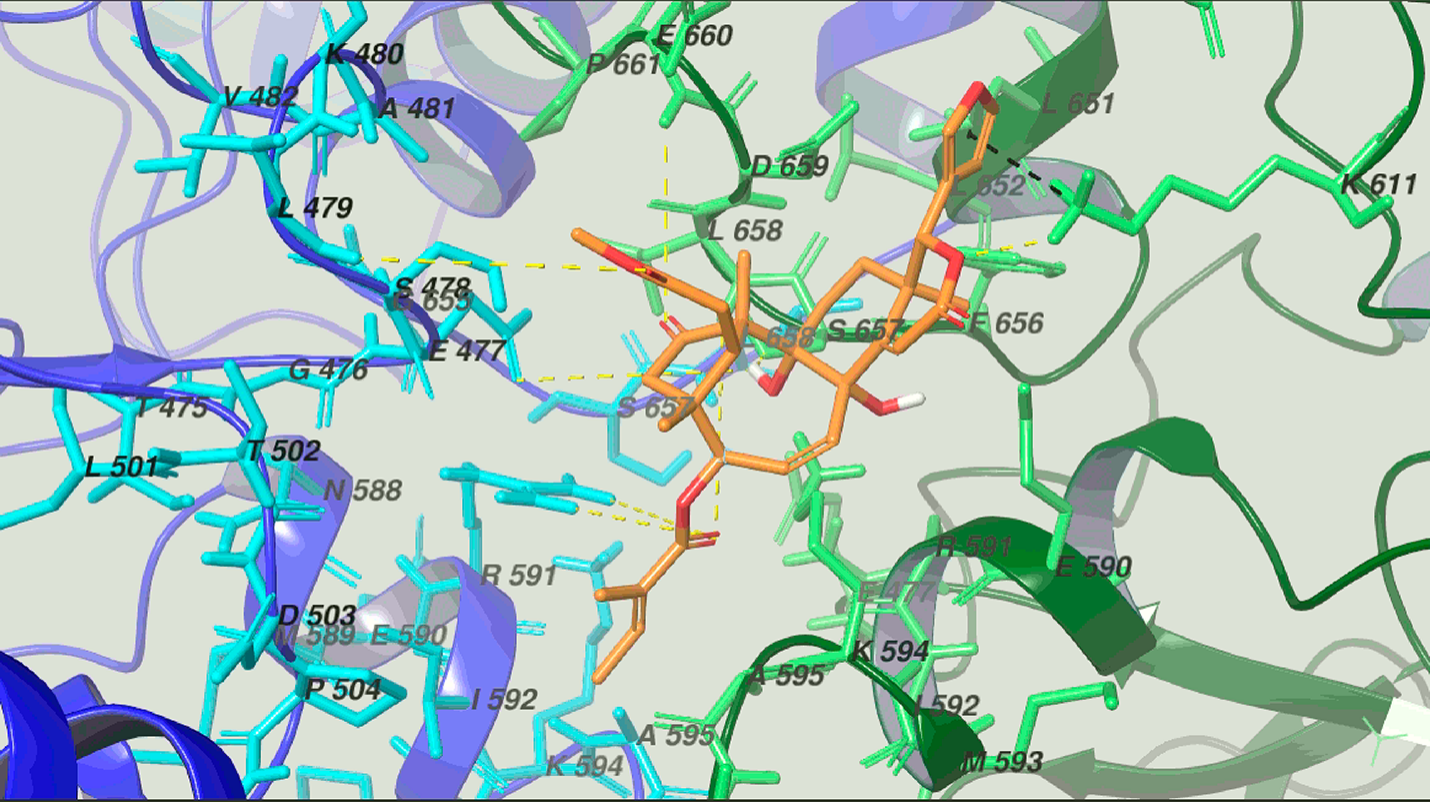
3D model of **1** (orange sticks) with Hsp90α (chain A and chain B are depicted as green and blue ribbons and sticks, respectively). The hydrogen bonds and π–cation interactions are reported in yellow and black lines.

The molecular docking approach was used to rationalize the biological activity of **1** toward the Hsp90 chaperone (Figure 5). For this purpose, the close active structure of the ATP-bound active state of Hsp82, yeast Hsp90 α homologue (PDB code: 2CG9),^41,42^ was used as a biological target. In accordance with the biological data reported above, the region of Hsp90 middle and C-terminus domains was used as the pharmacological site of interest. Moreover, considering the high plasticity of the chaperone during its mechanism of action, a flexible docking protocol (Induced Fit docking protocol of the Schrödinger Suite)^19^ was used for *in silico* study accounting for both receptor and ligand flexibility. Analyzing the computational results, the biological activity of the limonoid compound was attributed to the hydrophobic and polar interaction between **1** and Hsp90 chaperone. In particular, the furan ring at C-17 was involved in π–cation interaction with Lys611_chainA_, which also interacts with the oxygen atom of the lactone ring; the HO-9 acts as a H-bond donor with the side chains of Ser657_chainA_ and Glu477_chainB_. Furthermore, the CO groups at C-1, C-7, and C-1′ established hydrogen bonds with the side chains of Glu660_chainA_, Leu479_chainB_, and Arg591_chainB_, respectively. Moreover, the limonoid skeleton interacts with Glu590, Lys594, Ala595, Gln596, Ala597, Gln561, Tyr647, Leu651, Phe656, and Asp659 of chain A and with Ala481, Ser478, and Pro504 of chain B. Therefore, the computational analysis of the interaction pattern of the Hsp90α/**1** complex suggests a C-terminal inhibition mode, as corroborated by the influence of regulation of client proteins. Based on this result, it could be proposed that the cleavage of Hsp90 depends on some structural changes induced by the interaction between the protein and compound **1**, involving the C-terminal region of the protein. Such changes could expose specific residues to the action of intracellular proteases or destabilize the native structure of the protein, leading to its noncatalyzed cleavage.

## Experimental Section

### General Experimental Procedures

Optical rotations were measured on an Atago AP-300 digital polarimeter with a 1 dm microcell and a sodium lamp (589 nm). NMR data were recorded on a Bruker DRX-600 spectrometer at 300 K (Bruker BioSpinGmBH, Rheinstetten, Germany) equipped with a Bruker 5 mm TCI Cryoprobe at 300 K. All 2D NMR spectra were acquired in methanol-*d*_4_ or CDCl_3_, and standard pulse sequences and phase cycling were used for TOCSY, COSY, ROESY, HSQC, and HMBC spectra. HR-ESIMS data were obtained in the positive ion mode on a Q Exactive Plus mass spectrometer and an Orbitrap-based FT-MS system, equipped by an ESI source (Thermo Fischer Scientific Inc., Bremem, Germany). ESIMS data were acquired on an LCQ Advantage ThermoFinnigan spectrometer (ThermoFinnigan, USA), equipped with Xcalibur software. Column chromatography was performed over silica gel (70–220 mesh, Merck) or an Isolera Biotage flash purification system (flash silica gel 60 SNAP cartridge). RPHPLC separations were carried out using a Shimadzu LC-8A series pumping system equipped with a Shimadzu RID-10A refractive index detector and Shimadzu injector (Shimadzu Corporation, Japan) on a C_18_ μ-Bondapak column (30 cm × 7.8 mm, 10 μm, Waters-Milford) and a mobile phase consisting of a MeOH–H_2_O mixture at a flow rate of 2.0 mL/min. TLC separations were conducted using silica gel 60 F_254_ (0.20 mm thickness) plates (Merck, Germany) and Ce(SO_4_)_2_–H_2_SO_4_ as spray reagent (Sigma-Aldrich, Italy).

### Plant Material

The stem bark of *G. guidonia* was collected in December 2008, in the Estación Experimental Caparo (7°28′13″ N; 71°03′16″ O), in the southwest of Barinas State, Venezuela. The plant was identified by Prof. Dr. Pablo Meléndez, and a voucher specimen (N. 625) was deposited in the Herbarium MERF of the Universidad de Los Andes, Mérida, Venezuela. The stem bark of *C. odorata* was collected in April 2010 in Mérida, Venezuela, and identified by Ing. Juan Carmona. A voucher specimen (N. 06) has been deposited at the Herbarium MERF of the University de Los Andes, Mérida, Venezuela.

### Extraction and Isolation

The dried stem bark of *G. guidonia* (400 g) was powdered and extracted with solvents of increasing polarity, including *n*-hexane, CHCl_3_, and MeOH, by exhaustive maceration (2 L) to give 2.2, 15.0, and 4.6 g of the respective dried residues. Part of the CHCl_3_ extract (3.5 g) was subjected to CC (5 × 150 cm, collection volume 25 mL) over silica gel, eluting with CHCl_3_, followed by increasing concentrations of MeOH in CHCl_3_ (between 1% and 100%), collecting six major fractions (A–G). Fraction B (640.3 mg) was purified by RPHPLC with MeOH–H_2_O (3:2) as eluent to give compound **3** (1.9 mg, *t*_R_ 42 min). Fractions C (280 mg), E (105 mg), and F (506 mg) were subjected to RP-HPLC with MeOH–H_2_O (13:7) as eluent to give compounds **2** (3.0 mg, *t*_R_ 17 min), **1** (2.6 mg, *t*_R_ 20 min), and **3** (2.5 mg, *t*_R_ 22 min) from fraction C; compounds **4** (2.2 mg, *t*_R_ 16 min), **2** (2.2 mg, *t*_R_ 18 min), **1** (3.1 mg, *t*_R_ 20 min), and **3** (2.7 mg, *t*_R_ 22 min) from fraction E; and compound **4** (1.7 mg, *t*_R_ 16 min) from fraction F.

The dried and powdered stem bark of *C. odorata* (300 g) were extracted for 72 h with solvents of increasing polarity—*n*-hexane, CHCl_3_, CHCl_3_–MeOH (9:1), and MeOH—by exhaustive maceration (2 L) to give 3.2, 5.0, 4.5, and 28.5 g of the respective residues. Part of the CHCl_3_ extract (4.5 g) was subjected to flash silica gel column chromatography by a Biotage instrument (SNAP 340 g column, flow rate 90 mL/min, collection volume 30 mL), eluting with CHCl_3_ followed by increasing concentrations of MeOH in CHCl_3_ (between 1% and 30%), collecting 12 major fractions (A–L). Fractions D (347.9 mg) and H (104.8 mg) were subjected to RP-HPLC with MeOH–H_2_O (1:1) as eluent to give delevoyin D (2.2 mg, *t*_R_ 3 min), 7α,11β-limonoldiacetate (1.9 mg, *t*_R_ 4 min), compound **10** (2.1 mg, *t*_R_ 5 min), 7α-acetyldihydronomilin (2.3 mg, *t*_R_ 6 min), and compounds **11** (1.8 mg, *t*_R_ 7 min), **12** (2.3 mg, *t*_R_ 20 min), **13** (1.7 mg, *t*_R_ 35 min), and **14** (2.7 mg, *t*_R_ 45 min) from fraction D and **5** (1.0 mg, *t*_R_ 15 min) from fraction H. Fraction E (60.0 mg) was subjected to RP-HPLC with MeOH–H_2_O (3:2) as eluent to give compounds **10** (1.4 mg, *t*_R_ 4 min) and **11** (2.9 mg, *t*_R_ 5 min). Fractions F (197.0 mg) and I (207.1 mg) were subjected to RPHPLC with MeOH–H_2_O (9:11) as eluent to give swietenolide (2.6 mg, *t*_R_ 38 min) and compound **10** (2.8 mg, *t*_R_ 43 min) from fraction F and **6** (2.8 mg, *t*_R_ 13 min) from fraction I. Fraction K (229.0 mg) was subjected to RPHPLC with MeOH–H_2_O (2:3) as eluent to give compound **9** (2.8 mg, *t*_R_ 16 min). Part of the CHCl_3_–MeOH extract (4.4 g) was submitted to flash silica gel CC by a Biotage instrument (SNAP 340 g column, flow rate 90 mL/min, collection volume 30 mL), eluting with an *n*-hexane–CHCl_3_ (8:2) mixture followed by increasing concentrations of CHCl_3_ (from 80% to 100%), continuing with CHCl_3_ followed by increasing concentrations of MeOH in CHCl_3_ (between 1% and 100%), collecting 13 major fractions (A_1_–M_1_). Fraction B_1_ (382.0 mg) was subjected to RPHPLC with MeOH–H_2_O (7:13) as eluent to give 7α,11β-limonoldiacetate (1.9 mg, *t*_R_ 26 min) and delevoyin D (2.2 mg, *t*_R_ 54 min). Fraction C_1_ (149.7 mg) was subjected to RPHPLC with MeOH–H_2_O (3:2) as eluent to give compounds **12** (1.6 mg, *t*_R_ 17 min), **13** (2.7 mg, *t*_R_ 18 min), and **14** (1.8 mg, *t*_R_ 25 min). Fractions D_1_ (221.7 mg), E_1_ (57.4), and F_1_ (130.6) were subjected to RPHPLC with MeOH–H_2_O (1:1) as eluent to give swietenolide (1.0 mg, *t*_R_ 29 min) from fraction D_1_, compound **12** (1.2 mg, *t*_R_ 45 min) from fraction E_1_, and compound **8** (2.1 mg, *t*_R_ 16 min) and swietenolide (5.6 mg, *t*_R_ 20 min) from fraction F_1_. Fraction I_1_ (162.0 mg) was subjected to RPHPLC with MeOH–H_2_O (2:3) as eluent to give compound **7** (1.1 mg, *t*_R_ 39 min).

#### Compound **1**:

white amorphous powder; [α]_D_ −93 (*c* 0.1, MeOH); ^1^H and ^13^C NMR, see Tables 1 and 2; ESIMS *m*/*z* 605 [M + Na]^+^, 505 [M + Na – 100]^+^, 461 [M + Na – 100 – 44]^+^; HR-ESIMS *m*/*z* 605.2356 [M + Na]^+^ (calcd for C_32_H_38_O_10_Na, 605.2363), 583.2536 [M + H]^+^.

#### Compound **2**:

white amorphous powder; [α]_D_ −74 (*c* 0.1, MeOH); ^1^H and ^13^C NMR see Tables 1 and 2; HR-ESIMS *m*/*z* 591.2202 [M + Na]^+^ (calcd for C_31_H_36_O_10_Na, 591.2206), 505.1826 [M + Na – 86]^+^.

#### Compound **3**:

white amorphous powder; [α]_D_ −126 (c 0.1, MeOH); ^1^H and ^13^C NMR, see Tables 1 and 2; HR-ESIMS *m*/*z* 589.2412 [M + Na]^+^ (calcd for C_32_H_38_O_9_Na 589.2414), 489.1882 [M + Na – 100]^+^, 445.1983 [M + Na – 100 – 44]^+^.

#### Compound **4**:

white amorphous powder; [α]_D_ −178 (*c* 0.1, MeOH); ^1^H and ^13^C NMR, see Tables 1 and 2; HR-ESIMS *m*/*z* 593.2714 [M + Na]^+^ (calcd for C_32_H_42_O_9_Na 593.2727), 475.2071 [M + Na – 118]^+^.

#### Compound **5**:

white amorphous powder; [α]_D_ +2 (*c* 0.1, MeOH); ^1^H and ^13^C NMR, see Tables 3 and 4; HR-ESIMS *m*/*z* 611.2108 [M + Na]^+^ (calcd for C_30_H_36_O_12_Na, 611.2104), 551.1882 [M + Na – 60]^+^.

#### Compound **6**:

white amorphous powder; [α]_D_ −27 (*c* 0.1, MeOH); ^1^H and ^13^C NMR, see Tables 3–5; HR-ESIMS *m*/*z* 671.2309 [M + Na]^+^ (calcd for C_32_H_40_O_14_Na, 671.2316), 611.2089 [M + Na – 60]^+^, 567.2194 [M + Na – 60 – 44]^+^.

#### Compound **7**:

white amorphous powder; [α]_D_ −33 (*c* 0.1, MeOH); ^1^H and ^13^C NMR, see Tables 3 and 4; HR-ESIMS *m*/*z* 573.3041 [M + Na]^+^ (calcd for C_30_H_46_O_9_Na 573.3040), 513.2820 [M + Na – 60]^+^, 469.2921 [M + Na – 60 – 44]^+^, 453.2606 [M + Na – 120]^+^.

#### Compound **8**:

white amorphous powder; [α]_D_ −5 (*c* 0.1, MeOH); ^1^H and ^13^C NMR, see Tables 3 and 4; HR-ESIMS *m*/*z* 553.2038 [M + Na]^+^ (calcd for C_28_H_34_O_10_Na 553.2050), 493.1825 [M + Na – 60]^+^, 449.1931 [M + Na – 60 – 44]^+^.

#### Compound **9**:

white amorphous powder; [α]_D_ −1 (*c* 0.1, MeOH); ^1^H and ^13^C NMR, see Tables 3–5; HR-ESIMS *m*/*z* 627.2045 [M + Na]^+^ (calcd for C_30_H_36_O_13_Na 627.2054), 567.1841 [M + Na – 60]^+^.

### Reagents and Materials

The antibodies anti-Hsp70 (goat polyclonal sc-1060), anti-Cdc2 (mouse monoclonal, sc-8395), anti-phospho (Thr161)-Cdc2p34 (rabbit polyclonal, sc-101654), anti-Erk2 (mouse monoclonal sc-1647), anti-p-JNK (mouse monoclonal sc-6254), and JNK1 (rabbit polyclonal sc-571) were obtained from Santa Cruz Biotechnology (Delaware, CA, USA); anti-GAPDH (mouse monoclonal 437000) and anti-caspase3 (mouse monoclonal MA1-16843) were obtained from Thermo Fisher Scientific (Waltham, MA, USA); phospho-p44/42 MAPK (Erk1/2) (Thr202/Tyr204) (rabbit monoclonal #4376) was from Cell Signaling (Danvers, MA, USA); anti-Hsp90α (mouse monoclonal ADI-SPA-830) was from Enzo Life Science (Milan, Italy). Appropriate peroxidase-conjugated secondary antibodies were from Jackson Immuno Research (Baltimore, PA, USA).

### Surface Plasmon Resonance Analyses

SPR analyses were carried out by a Biacore 3000 optical biosensor (GE Healthcare, Milano, Italy), equipped with research-grade CM5 sensor chips (GE Healthcare), as reported elsewhere.^7^ Briefly, recombinant human Hsp90α (SPP-776, Stress-gen Bioreagents Corporation, Victoria, Canada) was dissolved at 100 μg/mL in NaOAc (50 mM, pH 5.0) and immobilized on a CM5 sensor chip surface using standard amine-coupling protocols and a flow rate of 5 μL/min, to obtain an optical density of 15 kRU. Compounds **1**–**14**, as well as radicicol used as a positive control, were dissolved in 100% DMSO to obtain 4 mM solutions and diluted 1:200 (v/v) in PBS (10 mM NaH_2_PO_4_, 150 mM NaCl, pH 7.4) to a final DMSO concentration of 0.1%. For each molecule, a five-point concentration series was set up (25, 50, 250 nM; 1 and 4 μM), and, for each sample, a complete binding study was carried out in triplicate. The experiments were performed at 25 °C, using a flow rate of 50 μL/min, with 60 s monitoring of association and 300 s monitoring of dissociation. Changes in mass, due to the binding response, were recorded as resonance units (RU). To obtain the dissociation constant (*K*_D_), these responses were fit to a 1:1 Langmuir binding model by nonlinear regression, using the BiaEvaluation sofware program (GE Healthcare). Simple interactions were suitably fitted to a single-site bimolecular interaction model (A + B = AB), yielding a single *K*_D_.

### Cell Culture and Treatment

HeLa (cervical carcinoma) and U937 (pro-monocytic, human myeloid leukemia) cell lines were purchased from the American Type Cell Culture (ATCC) (Rockville, MD, USA). HeLa cells were maintained in DMEM, and U937 were cultured in RPMI 1640, both supplemented with 10% FBS, 100 mg/L streptomycin, and penicillin 100 IU/mL at 37 °C in a humidified atmosphere of 5% CO_2_. To ensure logarithmic growth, cells were subcultured every 2 days. Stock solutions of compound in DMSO were stored in the dark at 4 °C. Appropriate dilutions were prepared in culture medium immediately prior to use. In all experiments, the final concentration of DMSO did not exceed 0.3% (v/v). PBMCs were isolated from buffy coats of healthy donors (kindly provided by the Blood Center of the Hospital of Battipaglia, Italy) by using standard Ficoll-Hypaque gradients. PBMCs were incubated with DMSO or compound **1** at 30 μM for 48 h.

### Cell Viability

HeLa and U937 cells were seeded in 96-well plates at a cell density of 7500 cells/well and 8 × 10^5^ cells/well, respectively. After 24 h, both cell lines were incubated for 48 h in the presence of compound at concentrations in the range 10–30 μM and radicicol (IC_50_ 250 nM) as a positive control. The number of viable cells was quantified by the MTT [3-(4,5-dimethylthiazol-2-yl)-2,5-diphenyl tetrazolium bromide] assay. Absorption at 550 nm for each well was assessed using Multiskan GO (Thermo Scientific). Half-maximal inhibitory concentration (IC_50_) values were calculated using GraphPad Prism 8. Experiments were performed in technical triplicates and repeated two times with similar results.

### Apoptosis and Cell Cycle

U937 cells were preliminarily synchronized by serum starvation and then seeded in six-well plates at a cell density of 8 × 10^5^ cell/well. After 24 h, they were incubated for 48 h in the presence of compound **1** at IC_50_ concentration (20 μm), with phenethyl isothiocyanate and DMSO as a positive and negative control, respectively. Apoptosis was evaluated by an annexin V–FITC/PI apoptosis detection kit (Dojindo EU GmbH, Munich, Germany), and the cell cycle was evaluated by propidium iodide (PI) staining of permeabilized cells, according to the available protocol, and flow cytometry (BD FACSCalibur flow cytometer, Becton Dickinson, San Jose, CA, USA). Data from 5000 events per sample were collected. The percentages of the elements in the hypodiploid region and in G1, S, and G2 phases of the cell cycle and apoptosis data were calculated using FlowJo (BD Biosciences). Experiments were performed in technical triplicates and repeated two times with similar results.

### Western Blot Analyses

Whole cell lysates (U937) for immunoblot analysis were lysed with RIPA buffer (20 mM Tris-HCl pH 7.5, 150 mM NaCl, 1 mM EDTA, 1% NP-40, 1% sodium deoxycholate, 2.5 mM sodium pyrophosphate) supplemented with protease and phosphatase inhibitors. Protein concentration was determined by a Bradford solution for protein determination (Applichem) using BSA as a standard. Proteins were fractionated on SDS-PAGE, transferred into nitrocellulose membranes, and immunoblotted with the appropriate primary antibody. Signals were visualized with the appropriate horseradish peroxidase-conjugated secondary antibody and enhanced chemiluminescence (Amersham Biosciences-GE Healthcare, NY, USA). Densitometric analyses were carried out using the ImageJ software. Experiments were performed in duplicate.

### Mass Spectrometry-Based Characterization of Different Hsp90α Forms

Proteins extracted from U937 cells following a 24 h treatment with 20 μM solution of compound **1** and from untreated cells (control) were fractionated on SDS-PAGE. The bands corresponding to the intact and truncated forms of Hsp90 (apparent molecular weight 90 and 70 kDa, respectively) were excised and subjected to a classical in-gel tryptic digestion. The resulting peptide mixtures were analyzed in positive ion full scan and dependent scan MS mode on a Q-Exactive Orbitrap mass spectrometer coupled with a nanoUltimate UHPLC system (Thermo Fisher Scientific, Waltham, MA, USA). Peptide separation was performed on a capillary EASY-Spray PepMap column (0.075 mm × 50 mm, 2 μm, Thermo Fisher Scientific) using aqueous 0.1% formic acid (A) and CH_3_CN containing 0.1% formic acid (B) as mobile phases and a linear gradient from 3% to 40% of B in 45 min and a 300 nL/min flow rate. Mass spectra were acquired over an *m*/*z* range from 400 to 1300. Dependent scan data were preliminarily analyzed to confirm the presence of Hsp90 in the digested gel-bands. To this aim, MS and MS/MS data underwent Mascot software (v2.5, Matrix Science, Boston, MA, USA) analysis using the nonredundant data bank UniprotKB/Swiss-Prot (release 2020_03). Parameter sets were as follows: trypsin cleavage; carbamidomethylation of cysteine as a fixed modification and methionine oxidation as a variable modification; a maximum of two missed cleavages; false discovery rate (FDR), calculated by searching the decoy database, 0.05. The data analysis of protein was carried out using the gene ontology tool in the UniProt Knowledgebase (UniProtKB; http://www.uniprot.org). Based on the achieved results, full mass chromatograms were analyzed using Xcalibur software by generating extracted ion spectra specific for the Hsp90 tryptic peptides completely covering the protein amino acidic sequence.

### Determination of the Relative Configurations of **1** and **3**

Chemical structures of all possible stereoisomers of **1** and **3**—**1a** (8R*, 9R*), **1b** (8R*, 9S*), **1c** (8S*, 9R*), and **1d** (8S*, 9S*) (see Figure S1) and **3a** (9R*) and **3b** (9S*) (Figure S1, Supporting Information)—were built using Maestro and optimized through the MacroModel software package,^19^ using the OPLS force field and the Polak-Ribier conjugate gradient algorithm (PRCG, maximum derivative less than 0.001 kcal/mol). Starting from the obtained 3D structures, exhaustive conformational searches were performed at the empirical molecular mechanics (MM) level following the subsequent scheme: (1) the Monte Carlo Multiple Minimum (MCMM) method (50 000 steps) of the MacroModel software package was used in order to allow a full exploration of the conformational space; (2) the Low Mode Conformational Search (LMCS) method (50 000 steps) as implemented in the MacroModel software package was used to integrate the conformational sampling. For each stereoisomer, all the conformers were minimized (PRCG, maximum deviation less than 0.001 kcal/mol) and compared. The selection of nonredundant conformers was performed using the “Redundant Conformer Elimination” module of Macromodel, choosing a 1.0 Å RMSD (root-mean-square deviation) minimum cutoff for saving structures and excluding the conformers differing by more than 13.0 kJ/mol (3.11 kcal/mol) from the most energetically favored conformation. Next, the obtained conformations of **1a**–**1d** and **3a** and **3b** were optimized at the QM level by using the MPW1PW91 functional and the 6-31G(d) basis set. Experimental solvent effects (MeOH) were reproduced using IEFPCM.^20^ On the obtained geometries, the MPW1PW91 functional, the 6-31G(d,p) basis set, and IEFPCM were used for calculating the ^1^H and ^13^C chemical shifts. Because such accuracy is seldom observed on sp^2^ carbon atoms, they have not been reported and considered in the configurational assignment in the present paper and in our preceding contributions.^43,44^ The final ^13^C and ^1^H NMR spectra for each of the investigated stereoisomers were built considering the influence of each conformer on the total Boltzmann distribution taking into account the relative energies. All ^13^C and ^1^H calculated chemical shifts were scaled to TMS. For the QM/NMR method, the experimental and calculated sets of data were compared atom by atom through the computation of the Δδ parameter, namely, Δδ = |δ_calc_ – δ_exp_|, where, for each accounted atom, δ_calc_ and δ_exp_ are the calculated and experimental values of chemical shifts, respectively. Based on this parameter, the difference between the experimental and the calculated values is estimated by computing an absolute difference (Δ) between the specific values, e.g., chemical shift data (δ), expressed in ppm. For each possible stereoisomer, obtained as reported above, for the final comparison, we used the statistical parameter MAE: MAE = ∑(Δδ)/*n*, namely, the summation (∑) of the *n* computed absolute δ error values (Δδ), normalized to the number of Δδ errors considered (*n*). The lowest MAE value indicates better accordance between the experimental and calculated data, suggesting the correct relative configuration.

### Molecular Docking Studies

A protein 3D model of the ATP-bound active state of Hsp82, a yeast Hsp90α homologue (PDB code: 2CG9), was prepared using the Schrödinger Protein Preparation Wizard workflow.^19^ Briefly, water molecules that were found 5 Å or more away from heteroatom groups were removed, and cap termini were included. Additionally, all hydrogen atoms were added, and bond orders were assigned. The resulting PDB files were converted to the MAE format. During the Induced Fit Workflow,^19^ the regions at the middle and C-terminal domain interface of Hsp90 (2CG9) were considered as the centroid for grid generation. Ring conformations of the compounds were sampled using an energy window of 2.5 kcal/mol; conformations featuring nonplanar conformations of amide bonds were penalized. The Induced Fit Workflow was performed using the default calculation protocol.

## Supporting Information Available

HR-ESIMS and NMR spectra of compounds **1**–**9**, Western blot analysis of p-CDC2 and CDC2 proteins, chemical and tridimensional structures of all the possible diastereoisomers for **1** and **3**, three-dimensional models and molecular docking of complexes between **8**, **9**, **11**, **13**, and Hsp90α, and Hsp90α peptides detected in the LC/MS analysis (PDF)

## Dedication

Dedicated to Dr. A. Douglas Kinghorn, The Ohio State University, for his pioneering work on bioactive natural products.
