# Supplementary material for: Limonoids from Guarea guidonia and Cedrela odorata: Heat Shock Protein 90 (Hsp90) Modulator Properties of Chisomicine D
Source: J Nat Prod. 2021 Mar 4;84(3):724–37. doi: 10.1021/acs.jnatprod.0c01217 (PMC8041370; doi:10.1021/acs.jnatprod.0c01217)
Supplement: Supplementary file 1 — np0c01217_si_001.pdf [file np0c01217_si_001.pdf]

Supporting Information

Limonoids from *Guarea guidonia* and *Cedrela odorata*. Heat Shock Protein 90 (Hsp90) Modulator Properties of Chisomicine D

Maria Laura Bellone,[‖] Cesar Muñoz Camero,[‖] Maria Giovanna Chini, Fabrizio Dal Piaz, Vanessa Hernandez, Giuseppe Bifulco, Nunziatina De Tommasi,[*] and Alessandra Braca

Table of Contents

Figure S1. ^{1}H NMR spectrum of compound **1** (CD_3OD, 600 MHz)

Figure S2. COSY spectrum of compound **1** (CD_3OD, 600 MHz)

Figure S3. HSQC spectrum of compound **1** (CD_3OD, 600 MHz)

Figure S4. HMBC spectrum of compound **1** (CD_3OD, 600 MHz)

Figure S5. HRESIMS of compound **1**

Figure S6. ^{1}H NMR spectrum of compound **2** (CD_3OD, 600 MHz)

Figure S7. COSY spectrum of compound **2** (CD_3OD, 600 MHz)

Figure S8. HSQC spectrum of compound **2** (CD_3OD, 600 MHz)

Figure S9. HMBC spectrum of compound **2** (CD_3OD, 600 MHz)

Figure S10. HRESIMS of compound **2**

Figure S11. ^{1}H NMR spectrum of compound **3** (CD_3OD, 600 MHz)

Figure S12. COSY spectrum of compound **3** (CD_3OD, 600 MHz)

Figure S13. HSQC spectrum of compound **3** (CD_3OD, 600 MHz)

Figure S14. HMBC spectrum of compound **3** (CD_3OD, 600 MHz)

Figure S15. HRESIMS of compound **3**

Figure S16. ^{1}H NMR spectrum of compound **4** (CD_3OD, 600 MHz)

Figure S17. COSY spectrum of compound **4** (CD_3OD, 600 MHz)

Figure S18. HSQC spectrum of compound **4** (CD_3OD, 600 MHz)

Figure S19. HMBC spectrum of compound **4** (CD_3OD, 600 MHz)

Figure S20. HRESIMS of compound **4**

Figure S21. ^{1}H NMR spectrum of compound **5** (CD_3OD, 600 MHz)

Figure S22. COSY spectrum of compound **5** (CD_3OD, 600 MHz)

Figure S23. HSQC spectrum of compound **5** (CD_3OD, 600 MHz)

Figure S24. HMBC spectrum of compound **5** (CD_3OD, 600 MHz)

Figure S25. HRESIMS of compound **5**

Figure S26. ^{1}H NMR spectrum of compound **6** (CD_3OD, 600 MHz)

Figure S27. COSY spectrum of compound **6** (CD_3OD, 600 MHz)

Figure S28. HSQC spectrum of compound **6** (CD_3OD, 600 MHz)

Figure S29. HMBC spectrum of compound **6** (CD_3OD, 600 MHz)

Figure S30. HRESIMS of compound **6**

Figure S31. ^{1}H NMR spectrum of compound **7** (CD_3OD, 600 MHz)

Figure S32. HSQC spectrum of compound **7** (CD_3OD, 600 MHz)

Figure S33. HMBC spectrum of compound **7** (CD_3OD, 600 MHz)

Figure S34. HRESIMS of compound **7**

Figure S35. ^{1}H NMR spectrum of compound **8** (CD_3OD, 600 MHz)

Figure S36. COSY spectrum of compound **8** (CD_3OD, 600 MHz)

Figure S37. HSQC spectrum of compound **8** (CD_3OD, 600 MHz)

Figure S38. HMBC spectrum of compound **8** (CD_3OD, 600 MHz)

Figure S39. HRESIMS of compound **8**

Figure S40. ^{1}H NMR spectrum of compound **9** (CD_3OD, 600 MHz)

Figure S41. COSY spectrum of compound **9** (CD_3OD, 600 MHz)

Figure S42. HSQC spectrum of compound **9** (CD_3OD, 600 MHz)

Figure S43. HMBC spectrum of compound **9** (CD_3OD, 600 MHz)

Figure S44. ^{1}H NMR spectrum of compound **9** ($CDCl_3$, 600 MHz)

Figure S45. HSQC spectrum of compound **9** ($CDCl_3$, 600 MHz)

Figure S46. HRESIMS of compound **9**

Figure S47. Western blot analysis of p-CDC2 and CDC2 proteins in cell treated with vehicle (DMSO) or radicicol. Normalized results of densitometric analysis are reported. The blots are representative of two different experiments providing similar results.

Figure S48. Chemical structures of all the possible diastereoisomers for **1** (**1a-1d**) and **3** (**3a** and **3b**).

Figure S49. Three dimensional structure of all the possible diastereoisomers for **1** (**1a**-**1d**).

Figure S50. Three-dimensional models of complexes between **8**, **9**, **11**, **13** and Hsp90α.

Molecular docking studies and computational details of **8**, **9**, **11** and **13**.

Table S1. Hsp90α peptides detected in the LC/MS analysis of tryptic digested bands at 90 kDa and 70 kDa of SDS-PAGE of the protein extracted from U937 cells treated with compound **1**.

References

Figure S1. ^{1}H NMR spectrum of compound **1** (CD_3OD, 600 MHz)

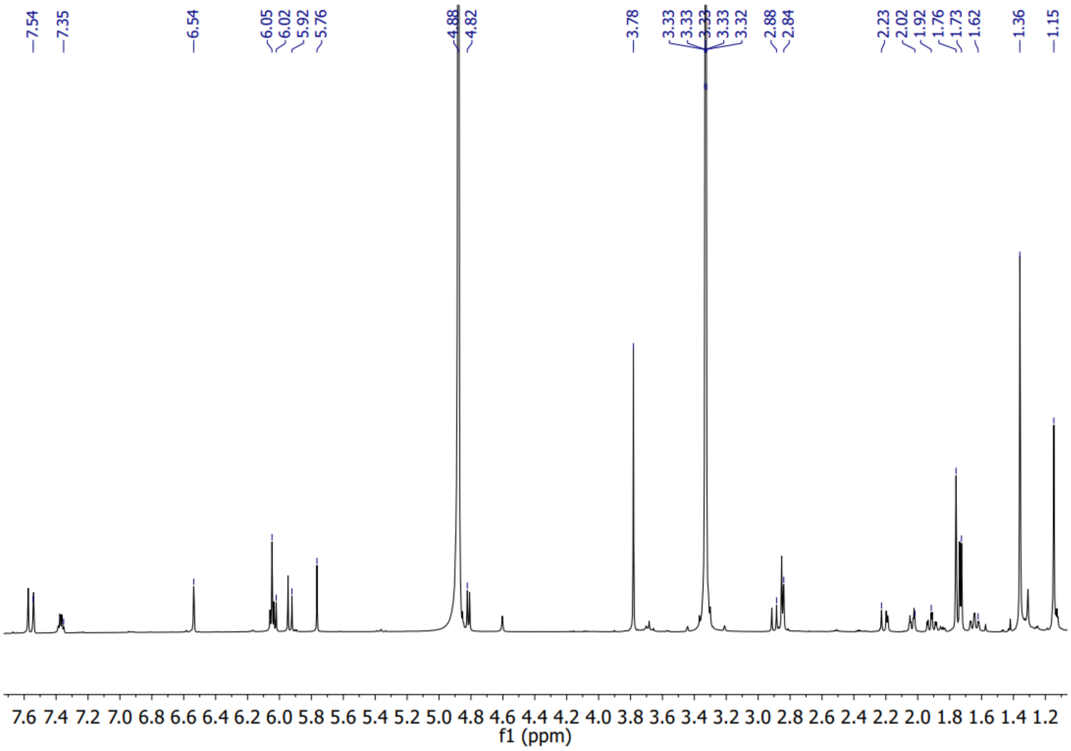

Figure S2. COSY spectrum of compound **1** (CD_3OD, 600 MHz)

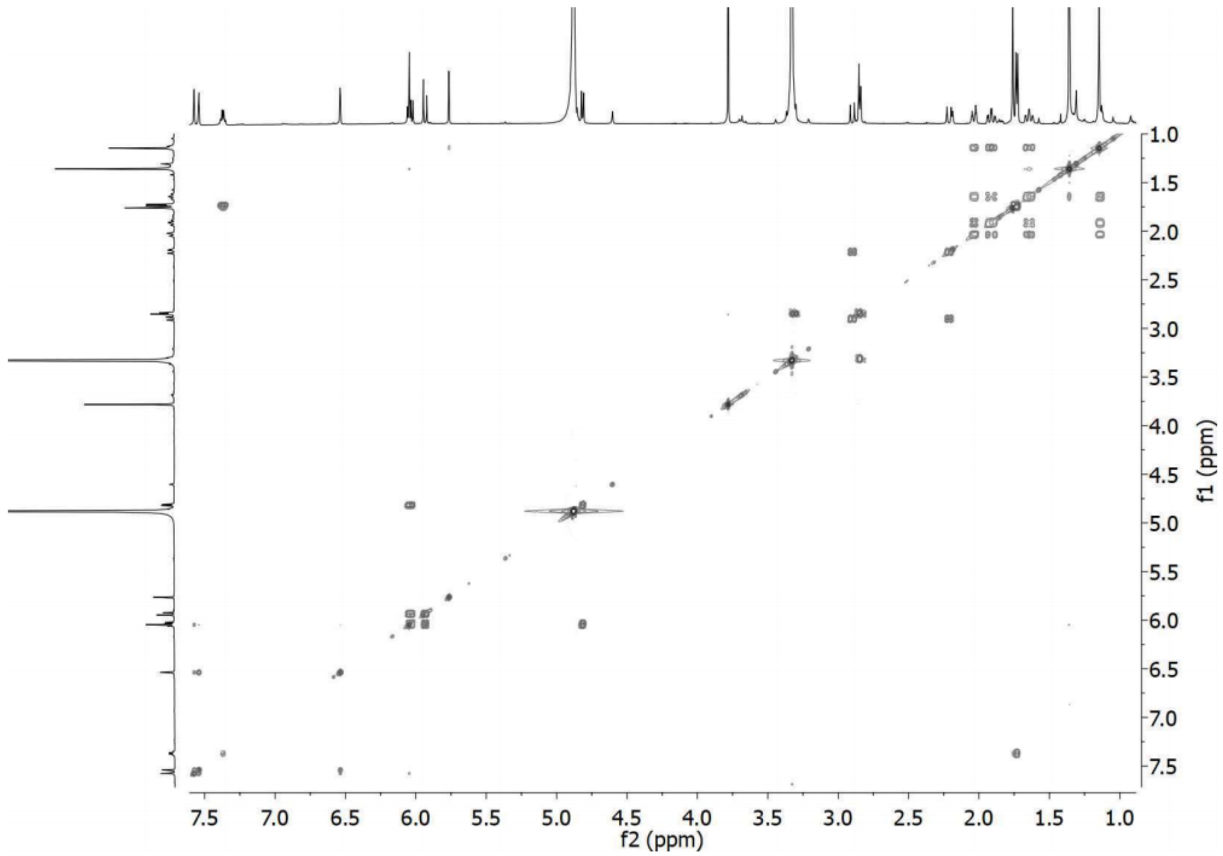

Figure S3. HSQC spectrum of compound **1** (CD_3OD, 600 MHz)

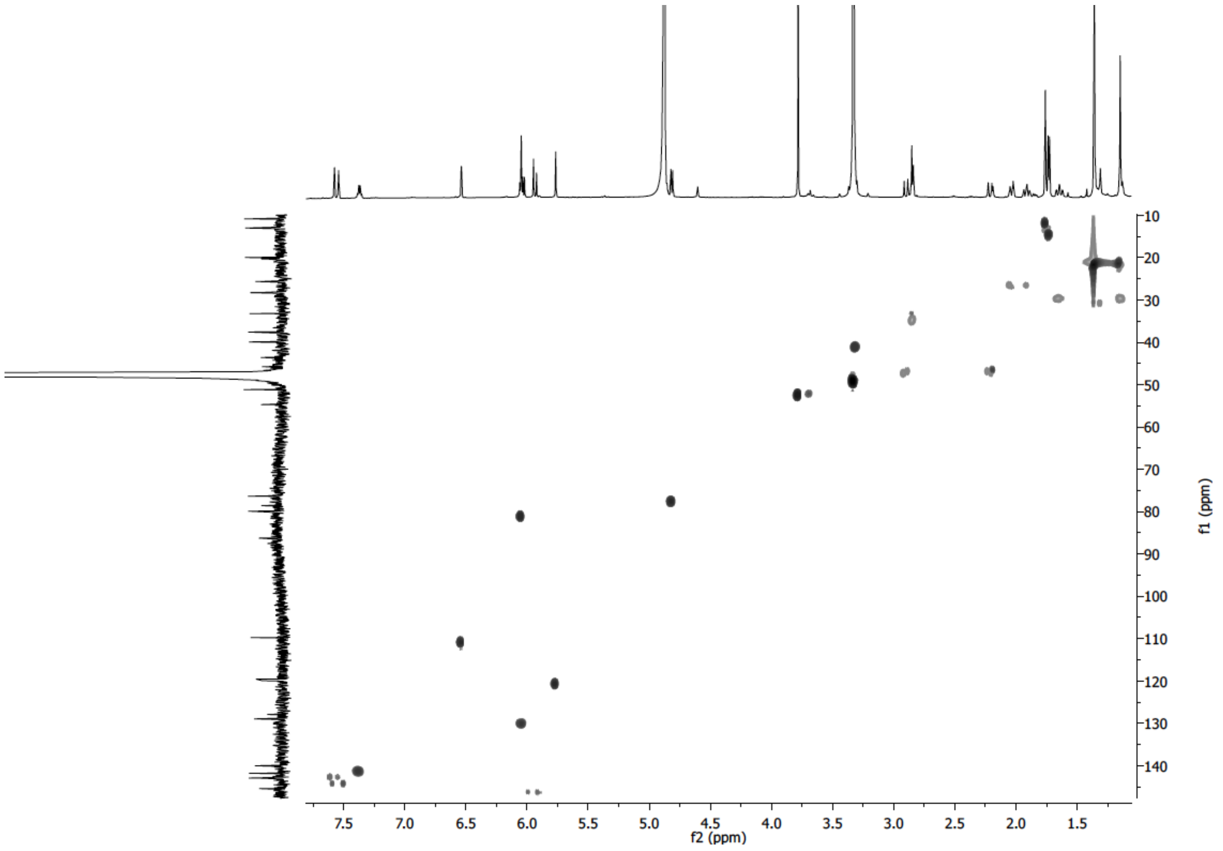

Figure S4. HMBC spectrum of compound **1** (CD_3OD, 600 MHz)

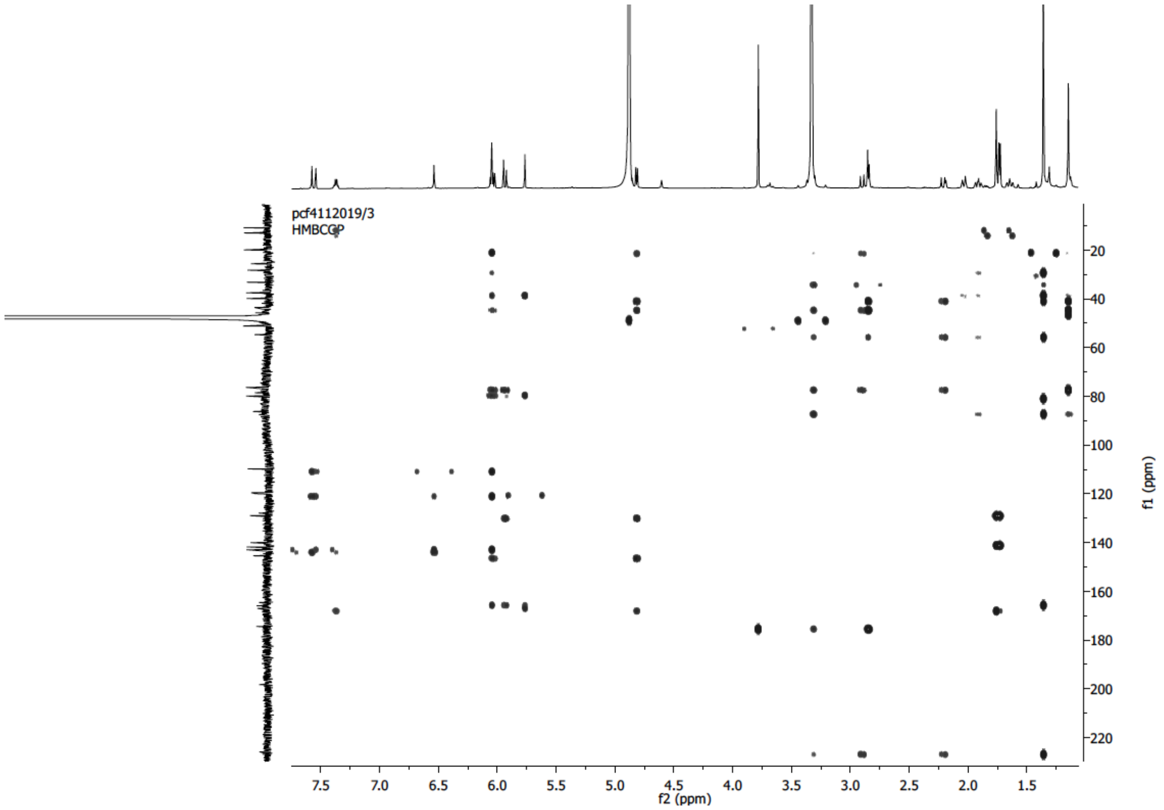

Figure S5. HRESIMS of compound **1**

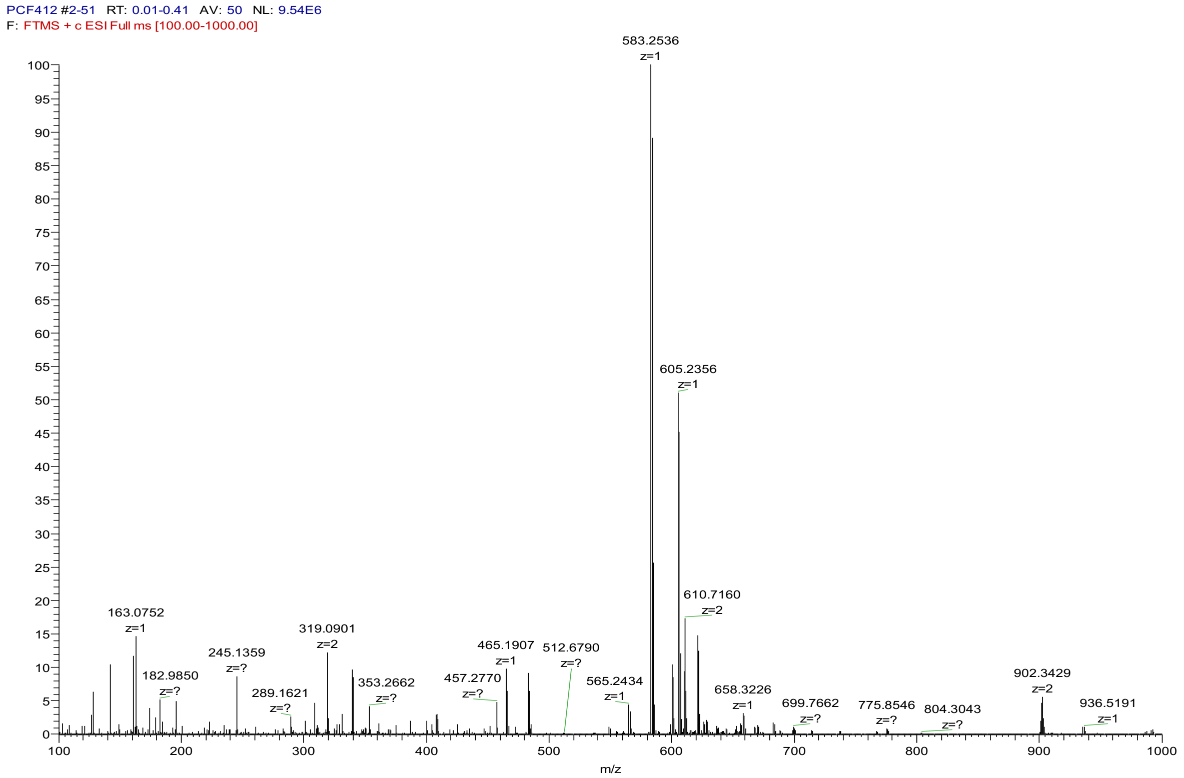

Figure S6. ^{1}H NMR spectrum of compound **2** (CD_3OD, 600 MHz)

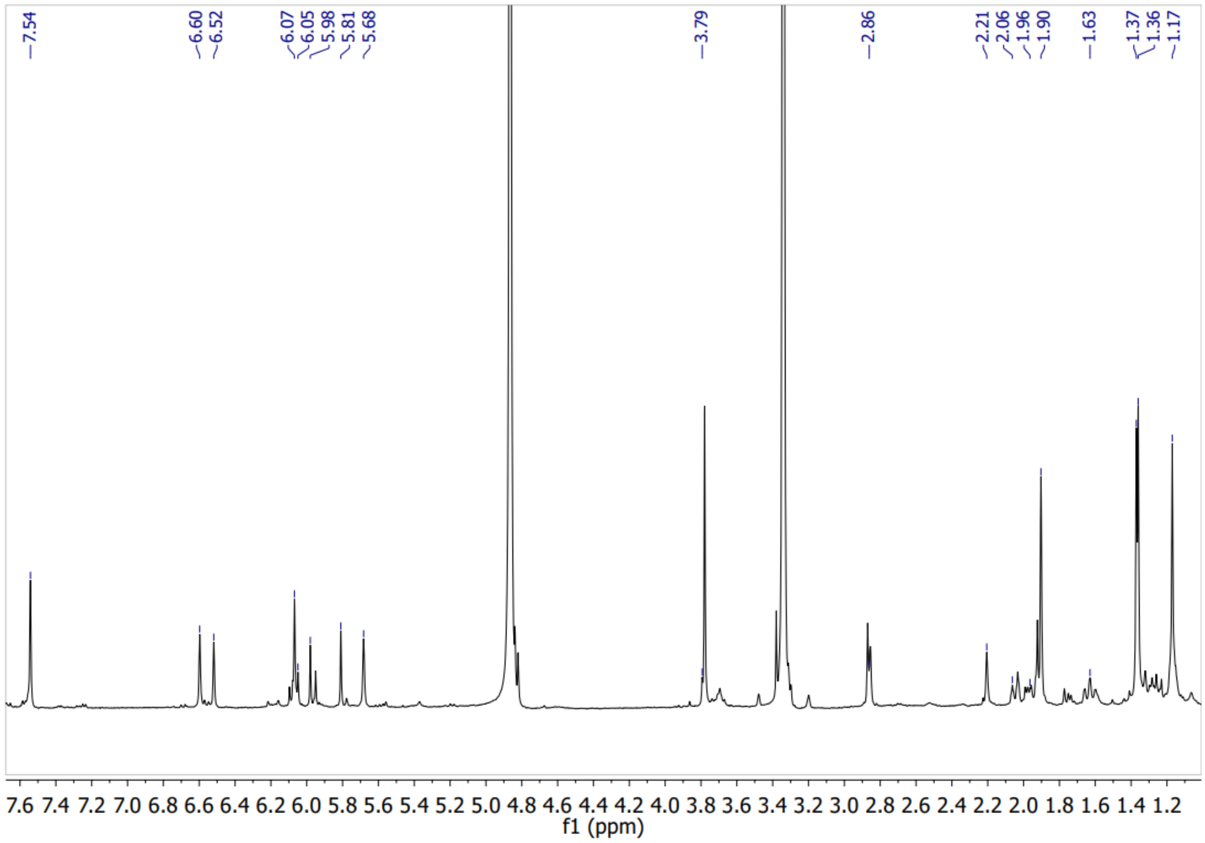

Figure S7. COSY spectrum of compound **2** (CD_3OD, 600 MHz)

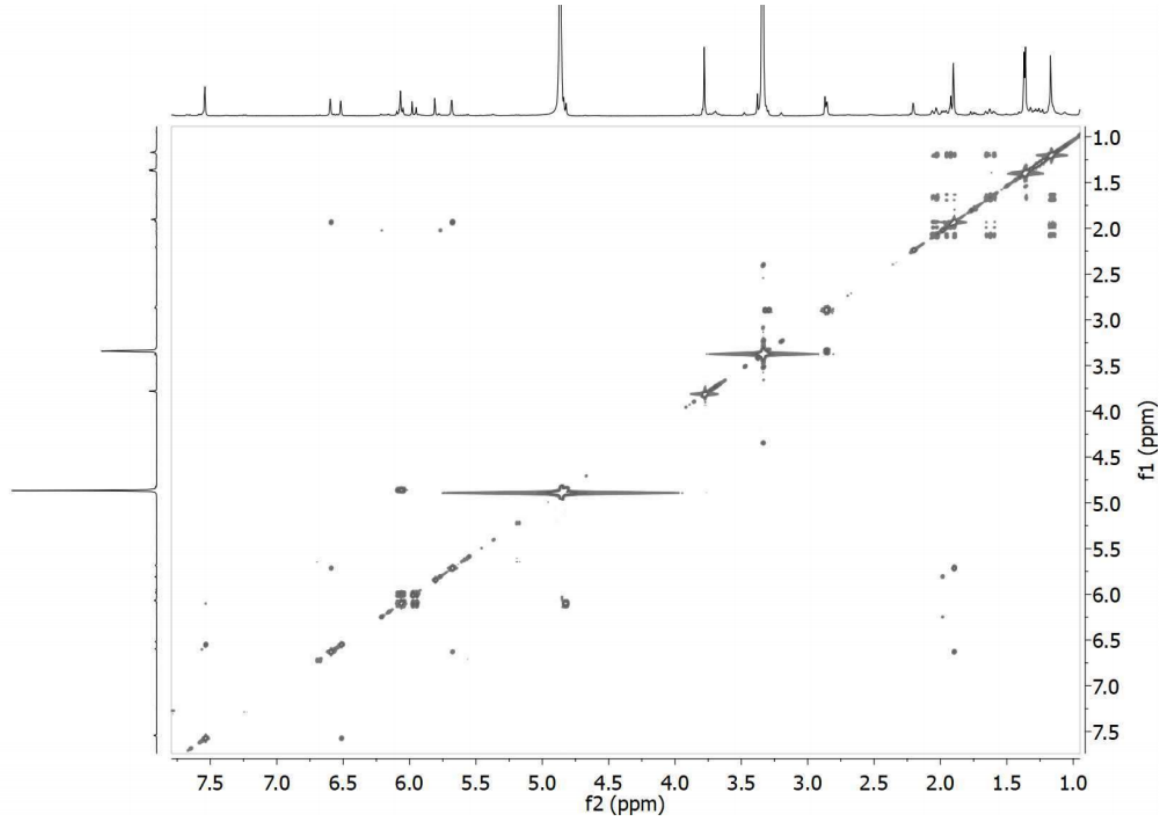

Figure S8. HSQC spectrum of compound **2** (CD_3OD, 600 MHz)

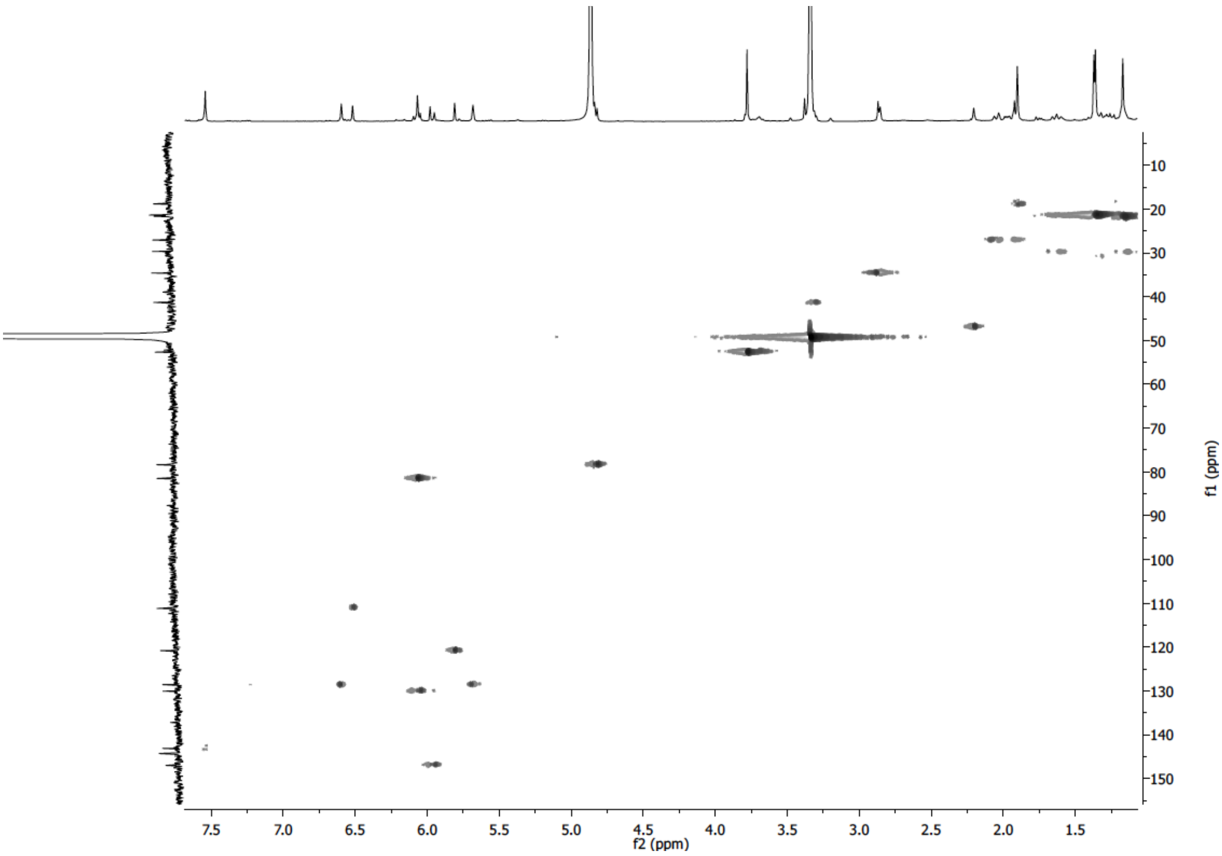

Figure S9. HMBC spectrum of compound **2** (CD_3OD, 600 MHz)

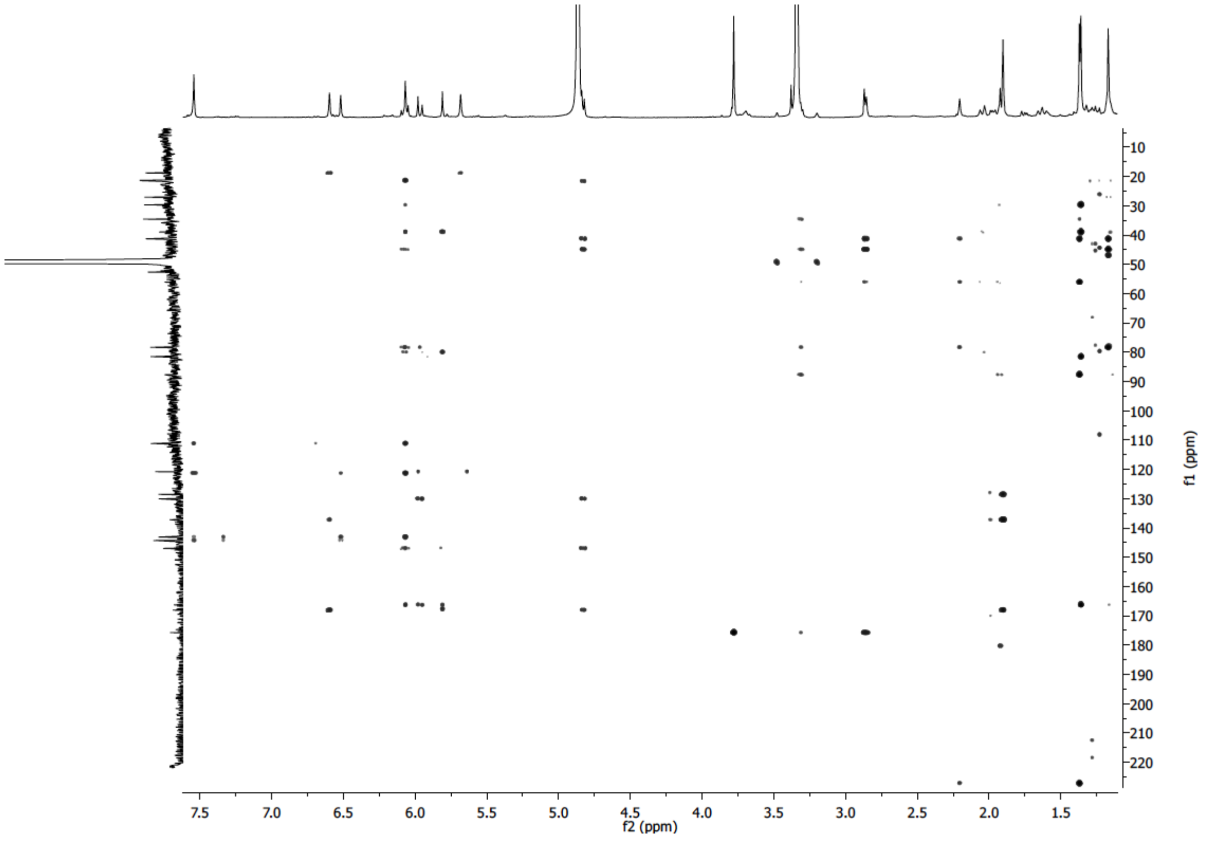

Figure S10. HRESIMS of compound **2**

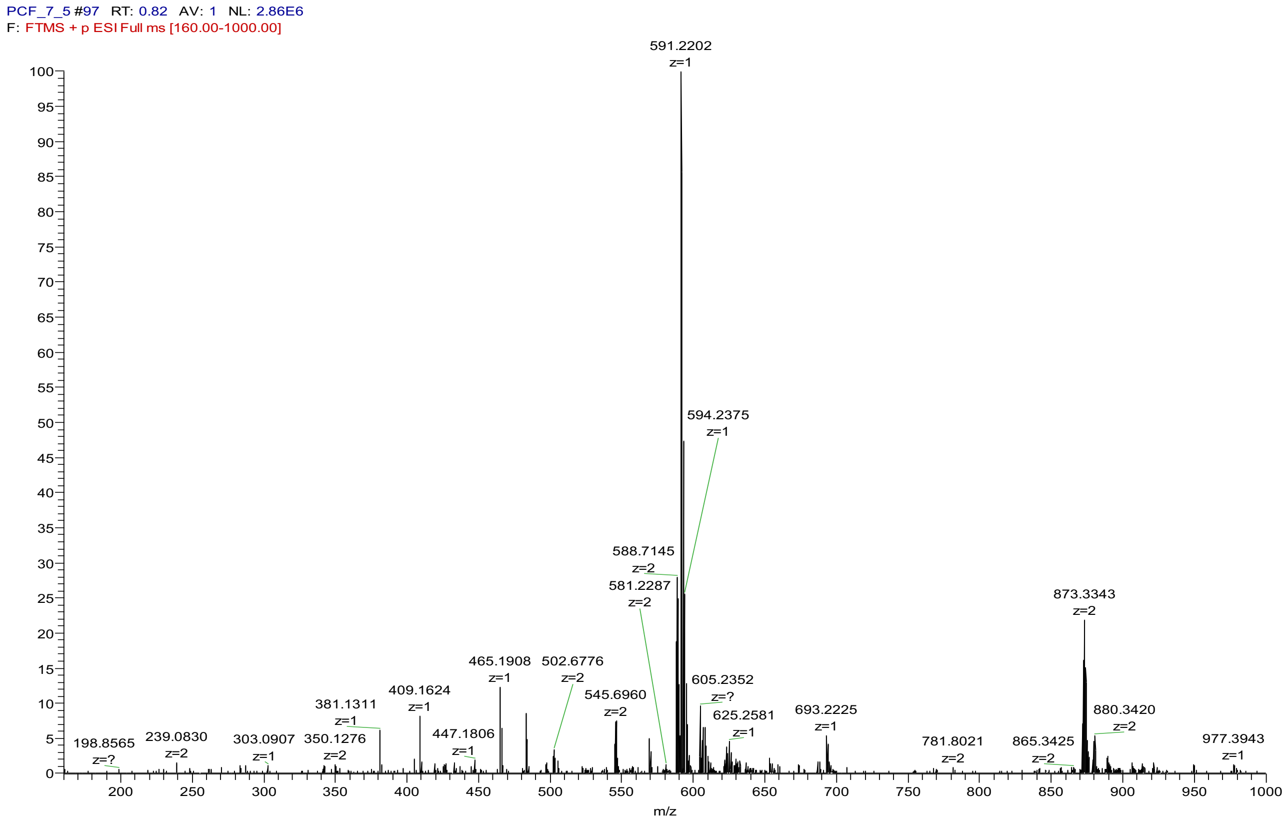

Figure S11. ^{1}H NMR spectrum of compound **3** (CD_3OD, 600 MHz)

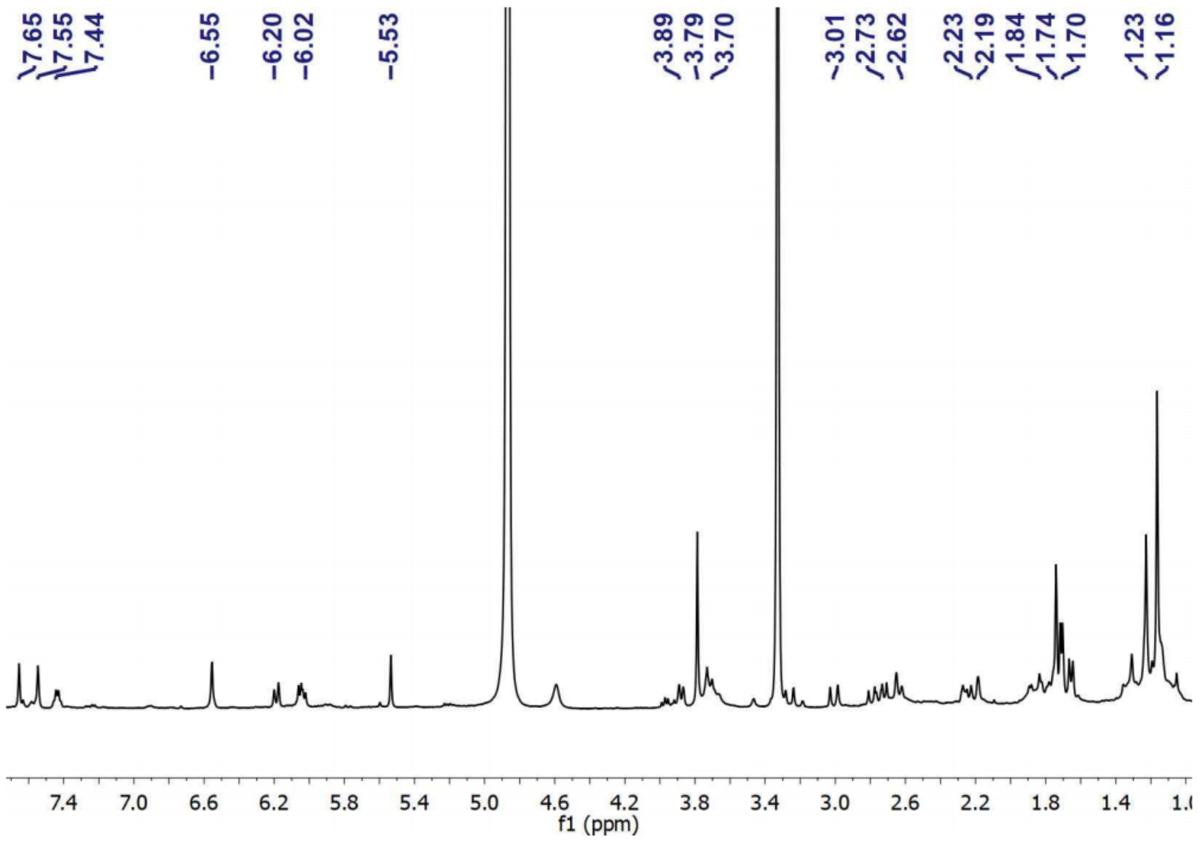

Figure S12. COSY spectrum of compound **3** (CD_3OD, 600 MHz)

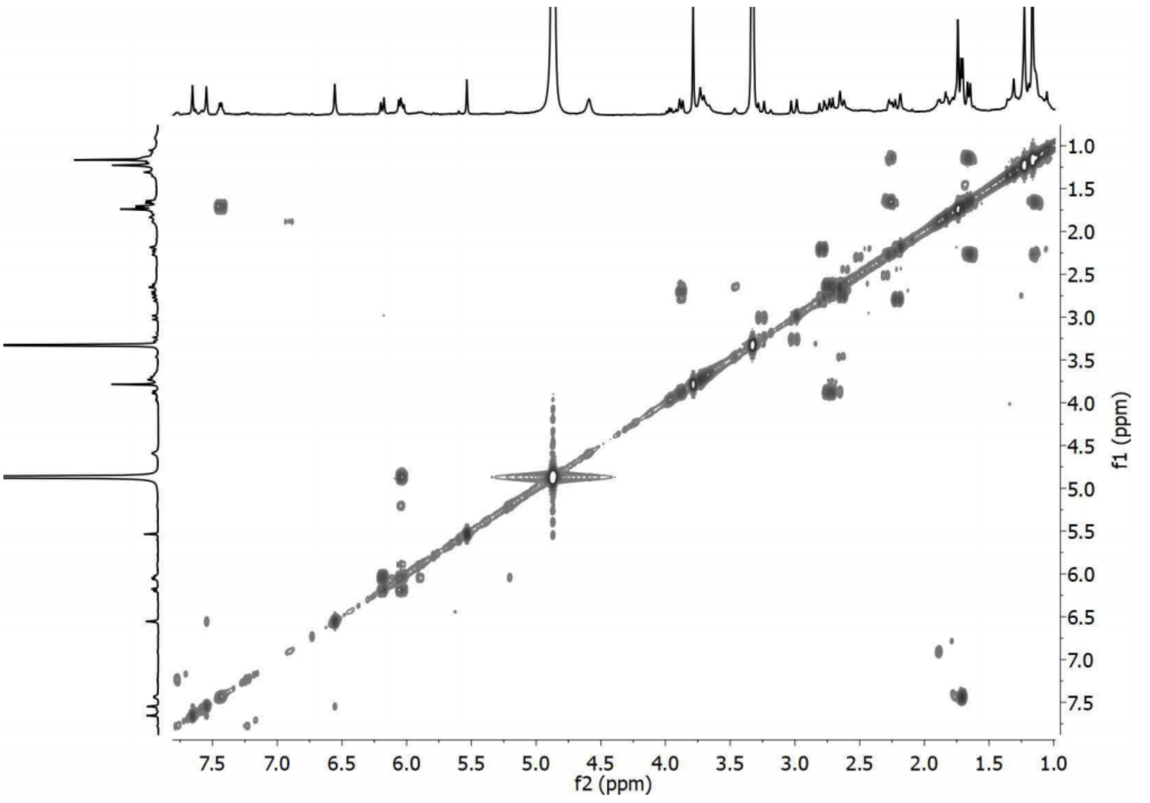

Figure S13. HSQC spectrum of compound **3** (CD_3OD, 600 MHz)

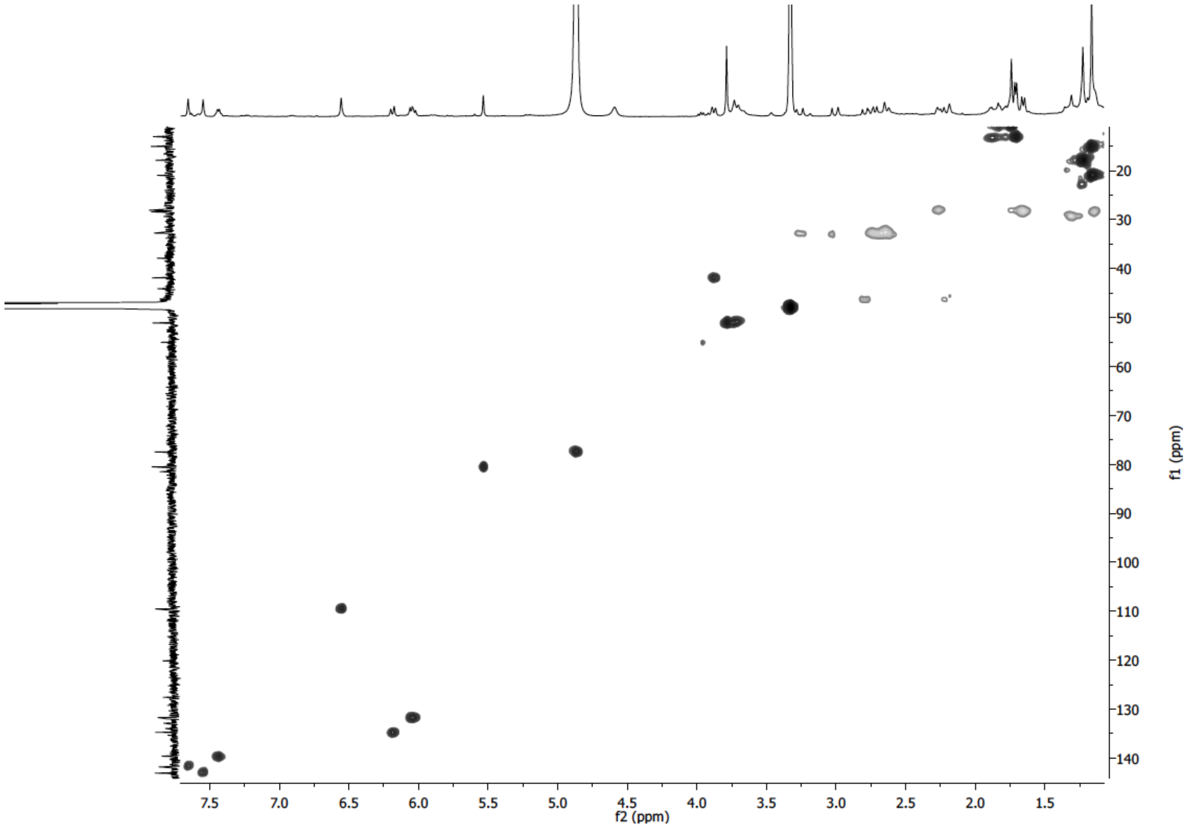

Figure S14. HMBC spectrum of compound **3** (CD_3OD, 600 MHz)

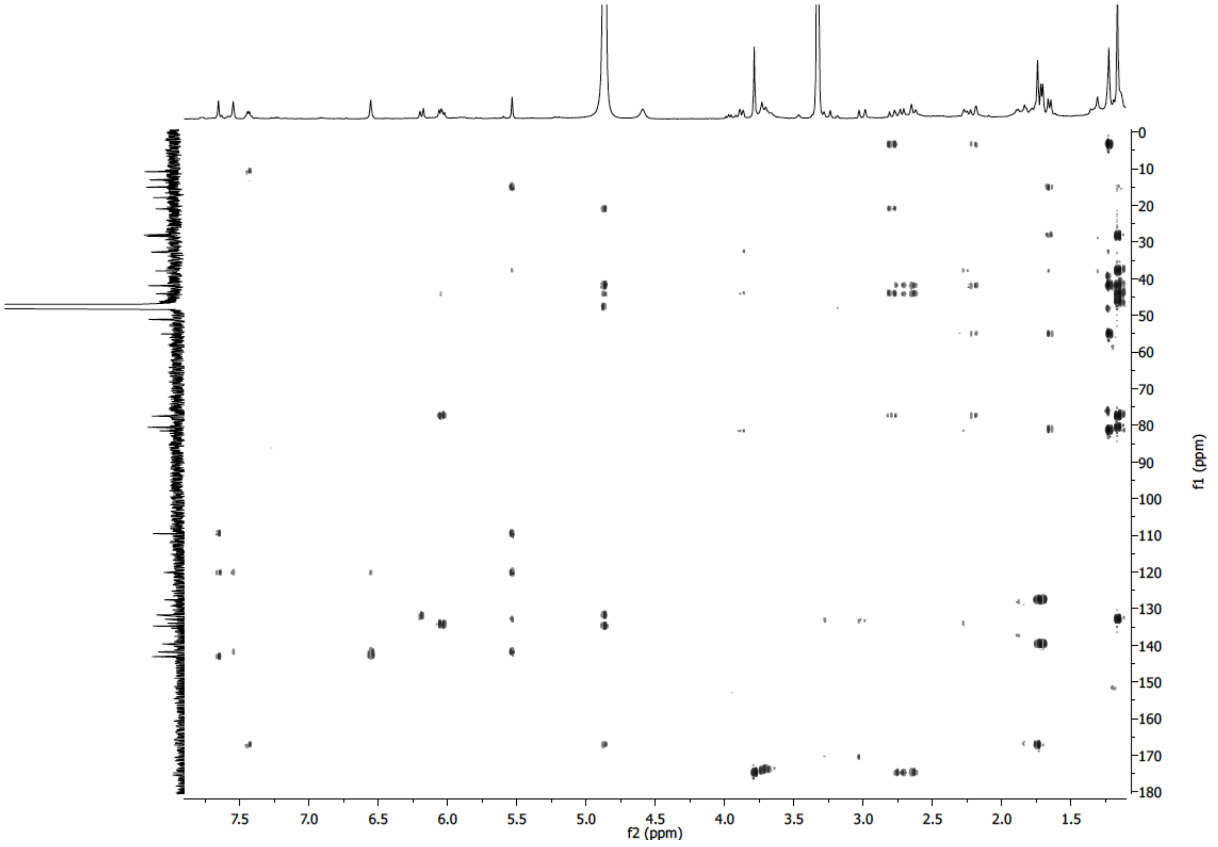

Figure S15. HRESIMS of compound **3**

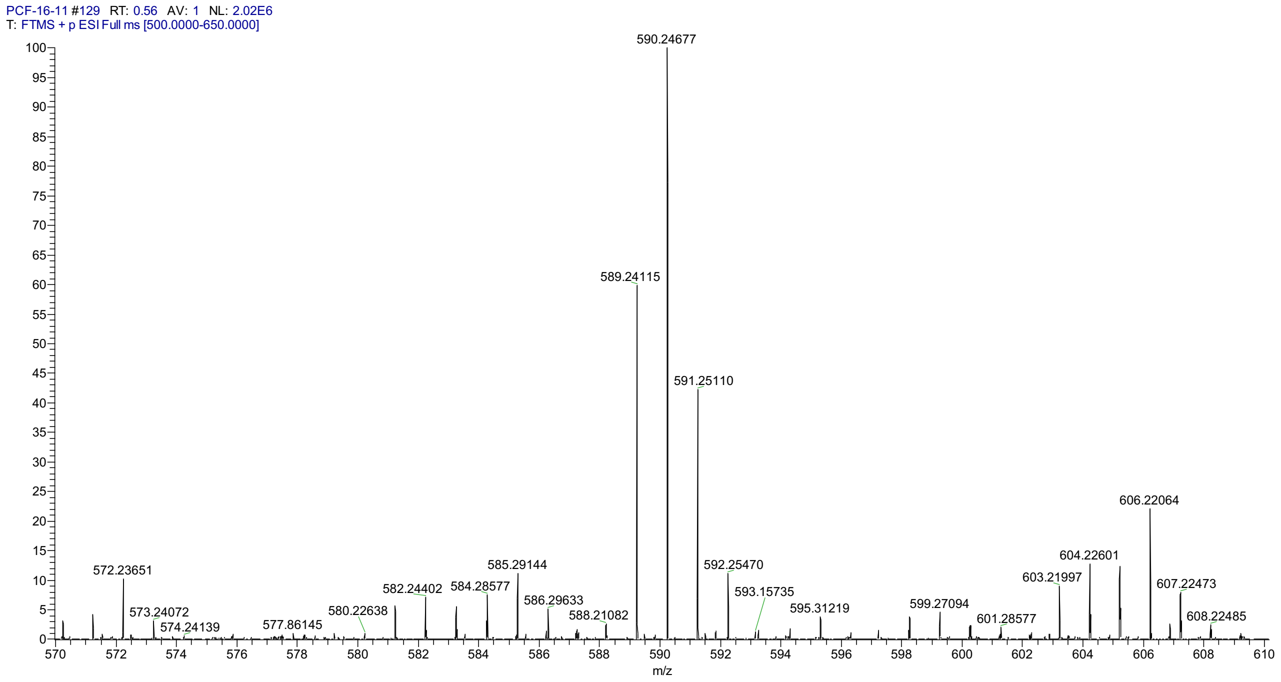

Figure S16. ^{1}H NMR spectrum of compound **4** (CD_3OD, 600 MHz)

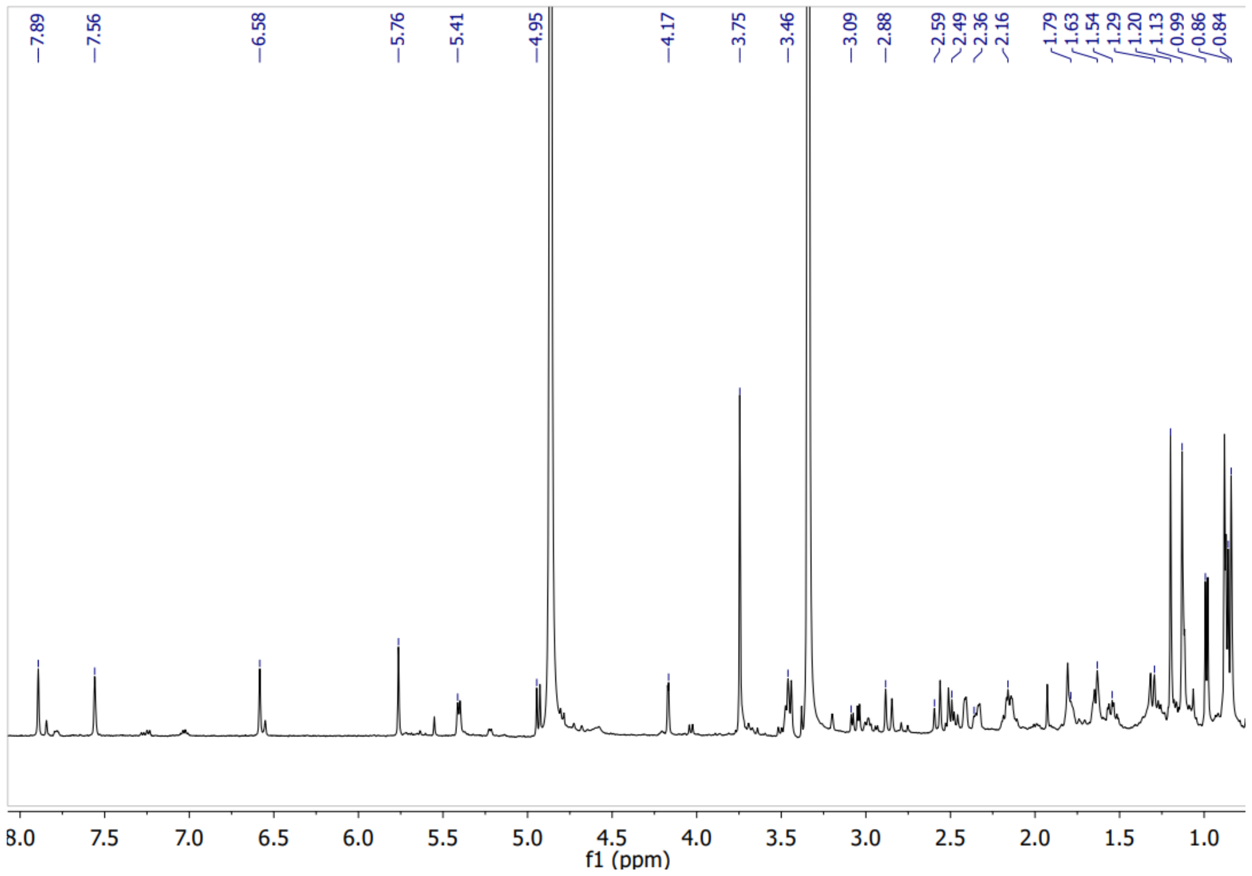

Figure S17. COSY spectrum of compound **4** (CD_3OD, 600 MHz)

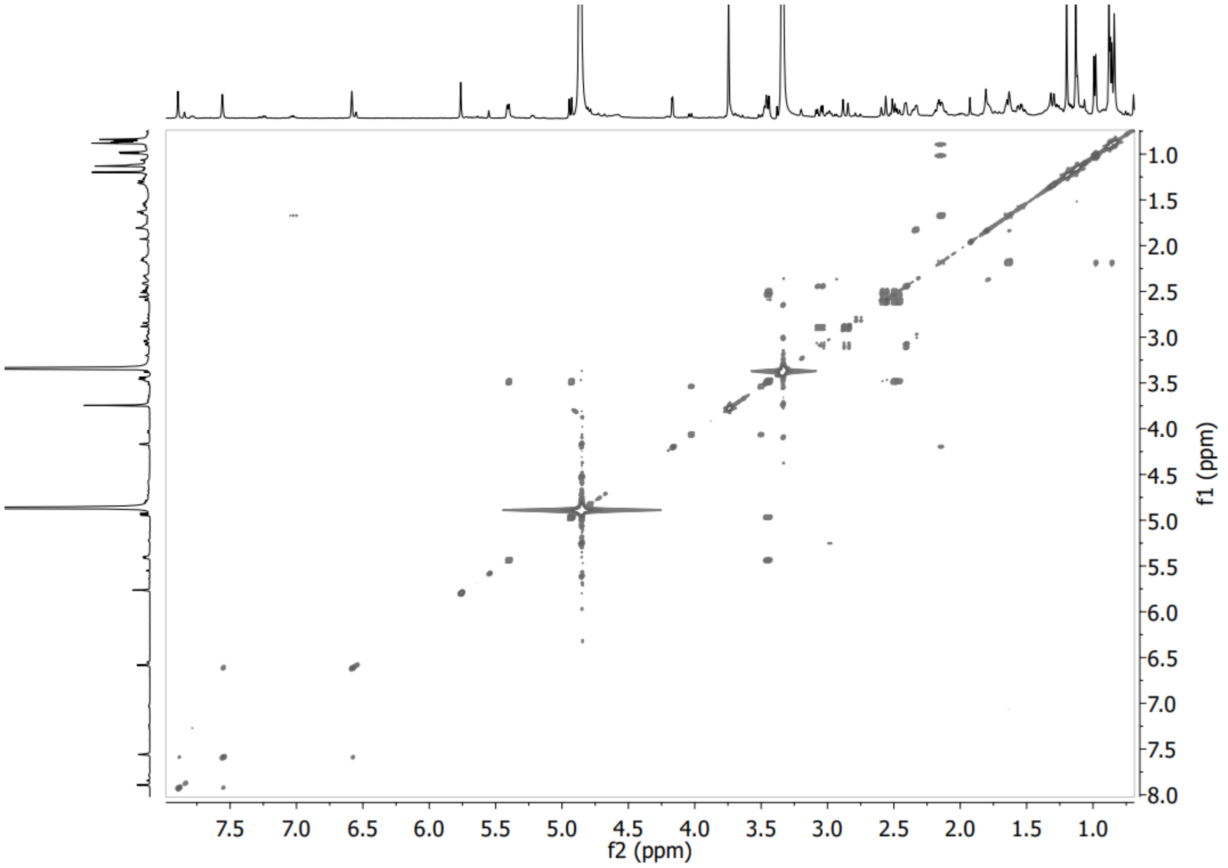

Figure S18. HSQC spectrum of compound **4** (CD_3OD, 600 MHz)

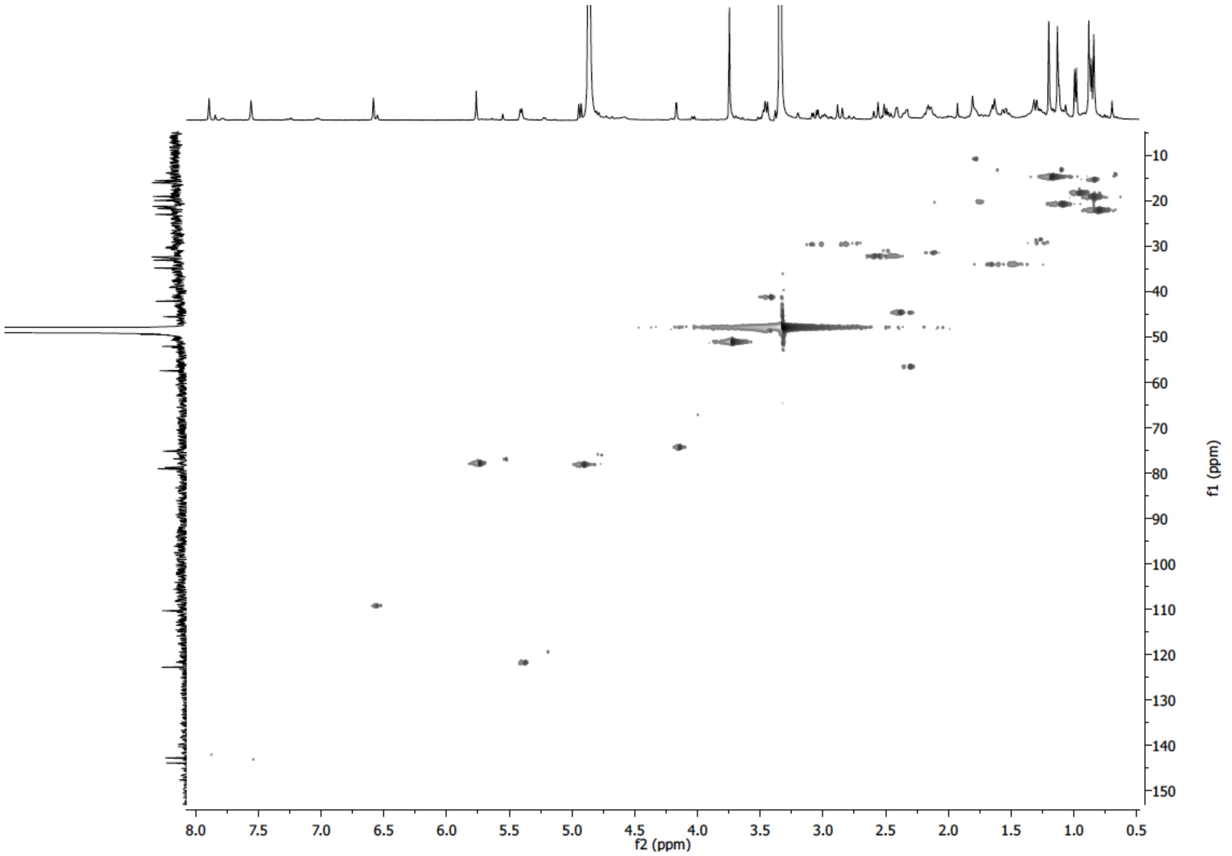

Figure S19. HMBC spectrum of compound **4** (CD_3OD, 600 MHz)

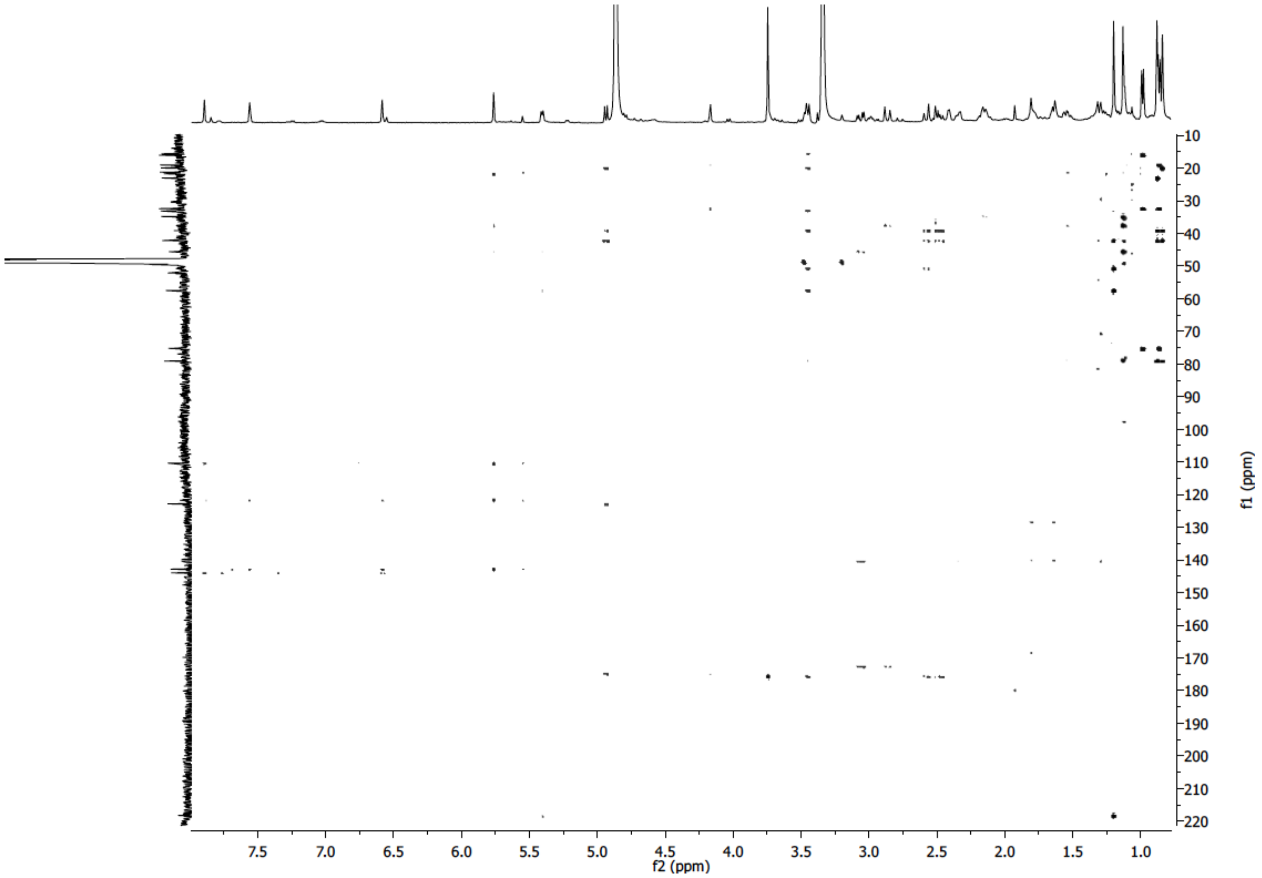

Figure S20. HRESIMS of compound **4**

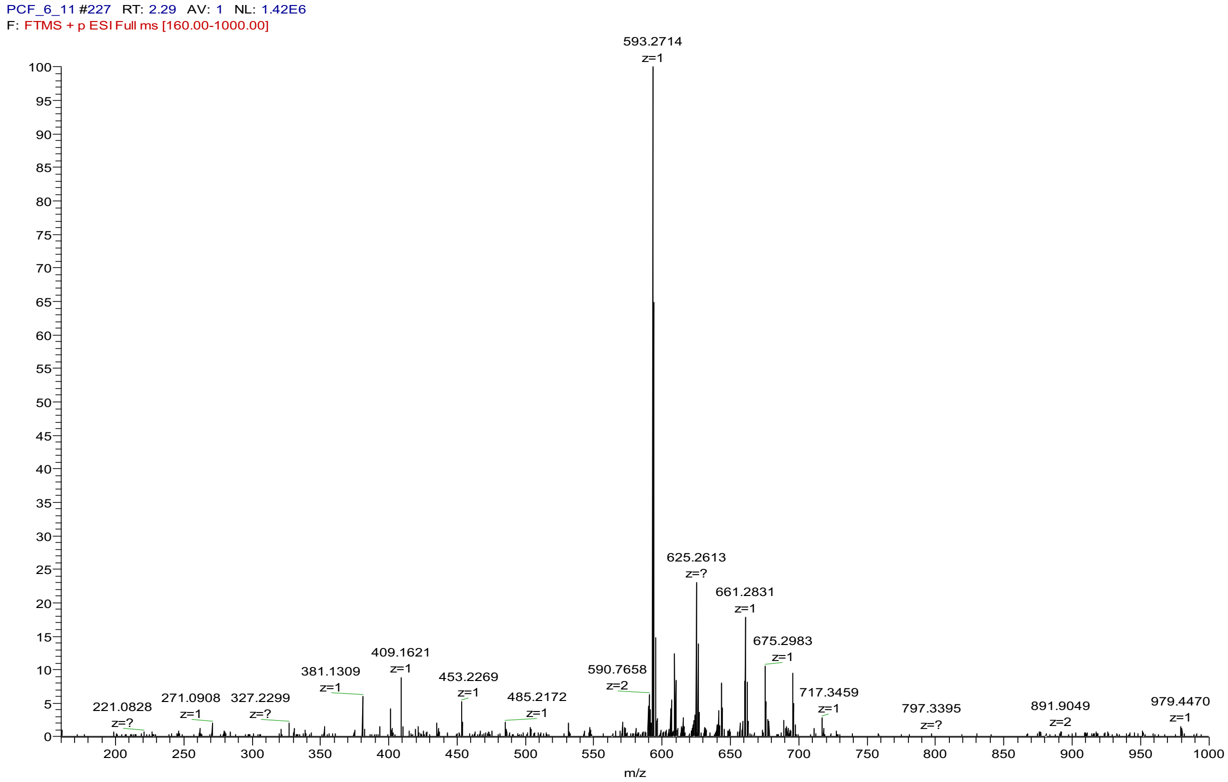

Figure S21. ^{1}H NMR spectrum of compound **5** (CD_3OD, 600 MHz)

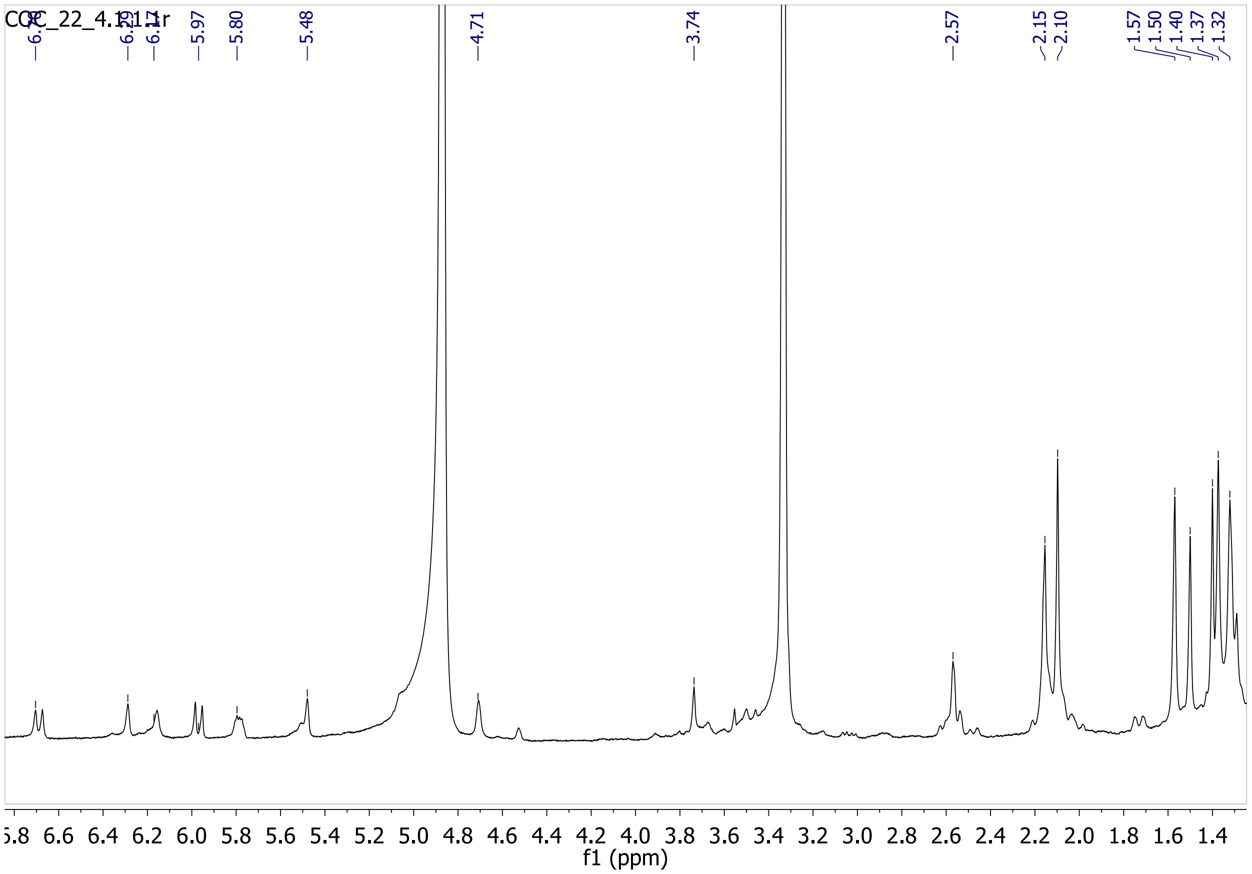

Figure S22. COSY spectrum of compound **5** (CD_3OD, 600 MHz)

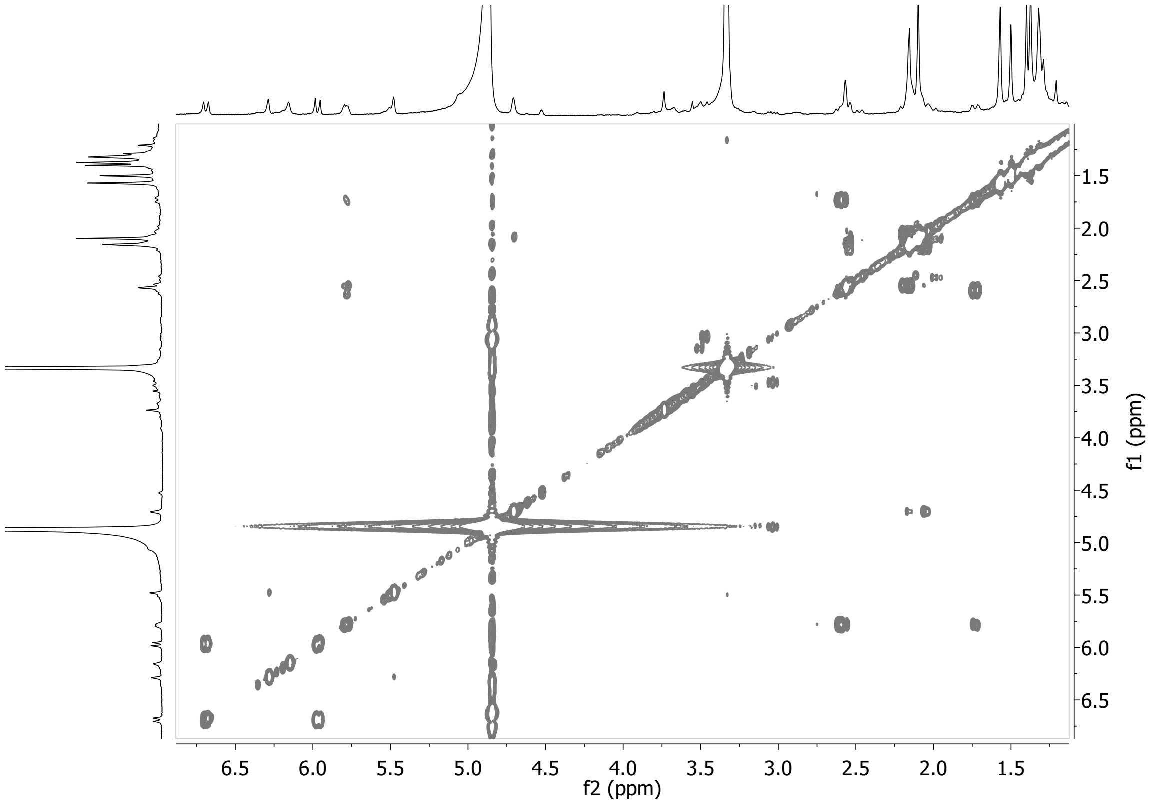

Figure S23. HSQC spectrum of compound **5** (CD_3OD, 600 MHz)

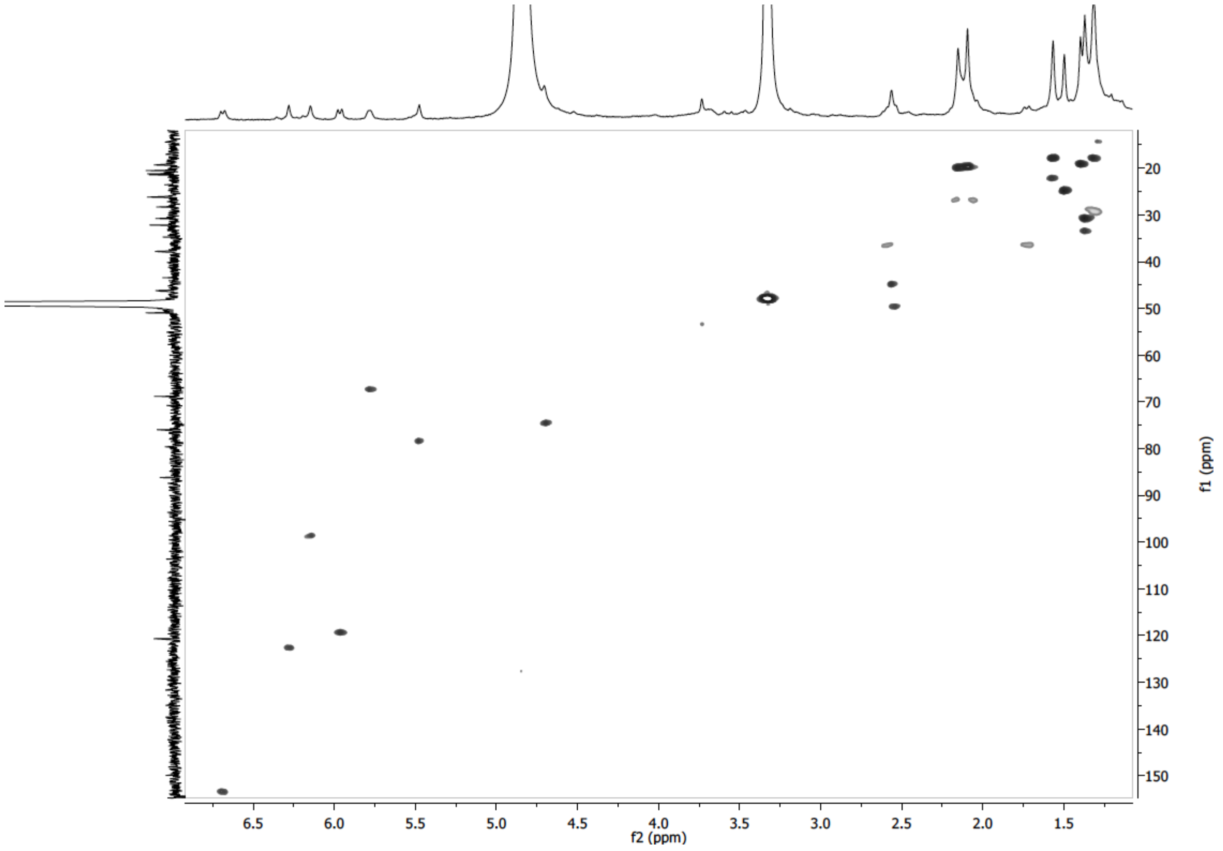

Figure S24. HMBC spectrum of compound **5** (CD_3OD, 600 MHz)

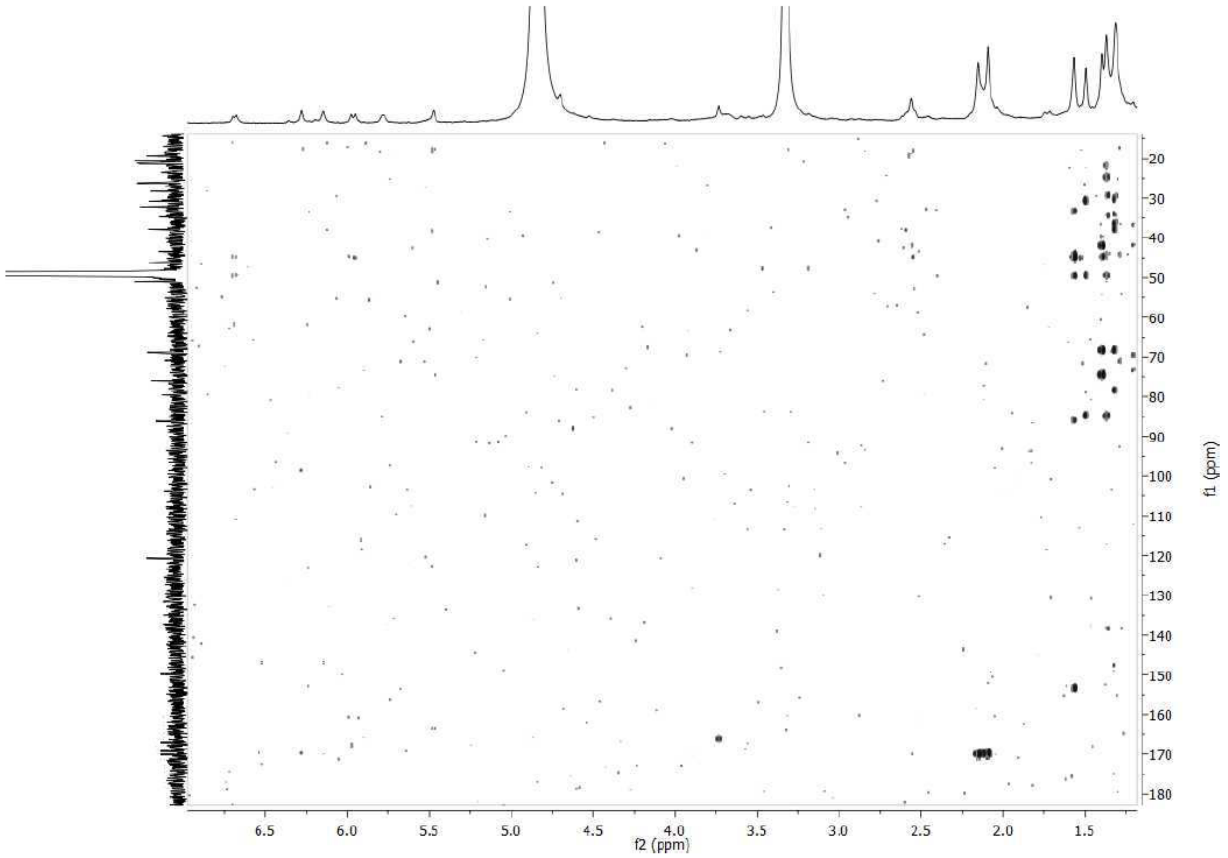

Figure S25. HRESIMS of compound **5**

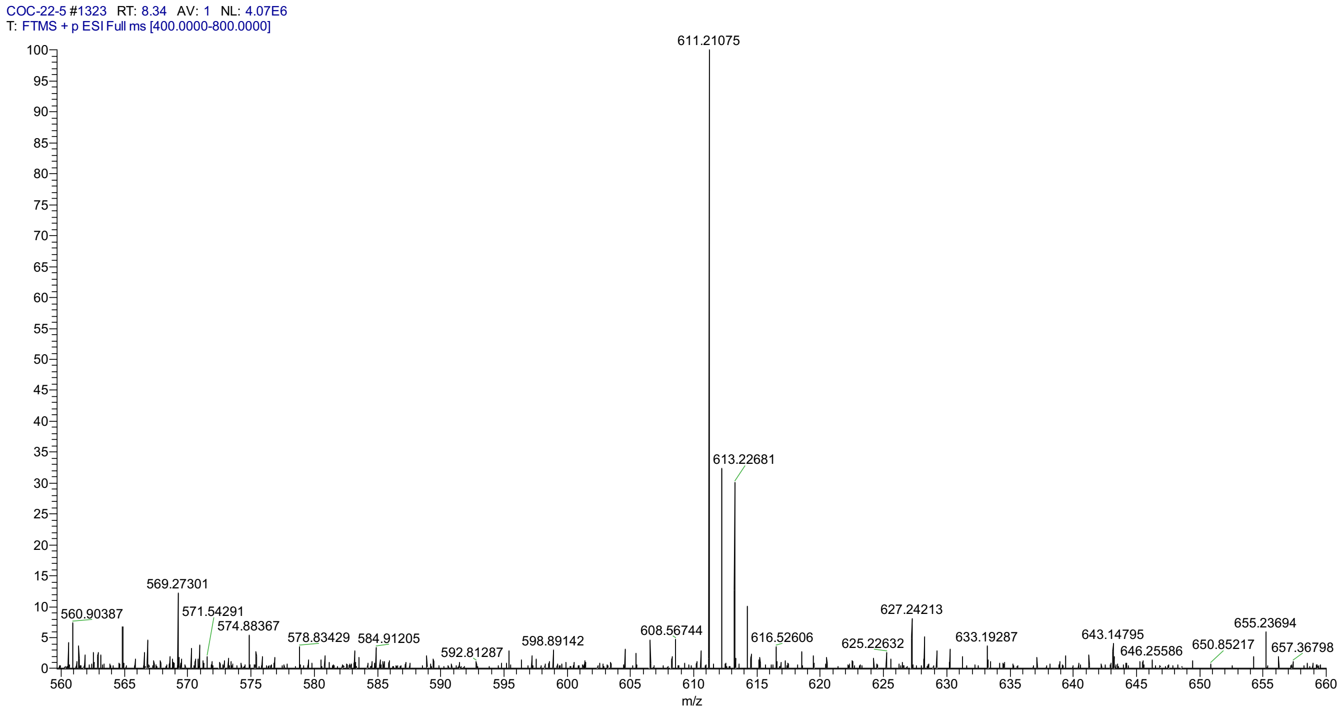

Figure S26. ^{1}H NMR spectrum of compound **6** (CD_3OD, 600 MHz)

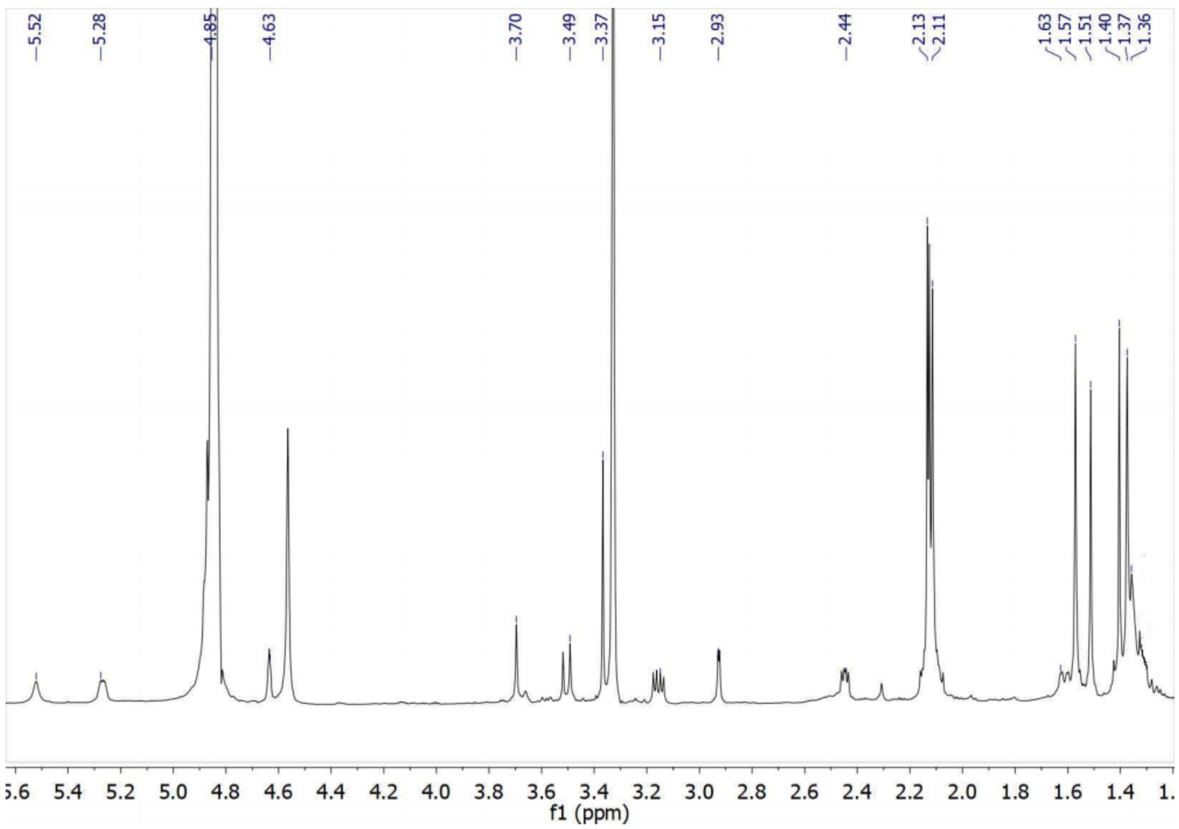

Figure S27. COSY spectrum of compound **6** (CD_3OD, 600 MHz)

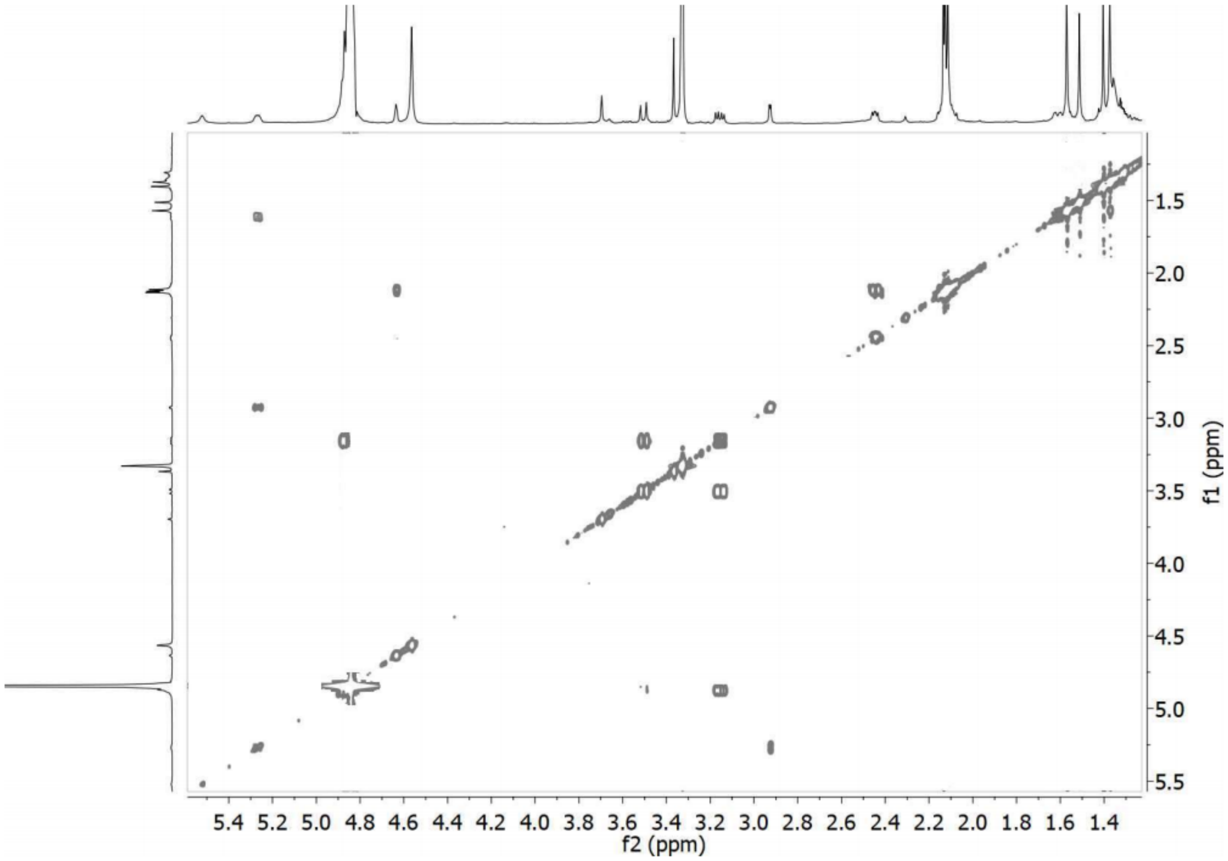

Figure S28. HSQC spectrum of compound **6** (CD_3OD, 600 MHz)

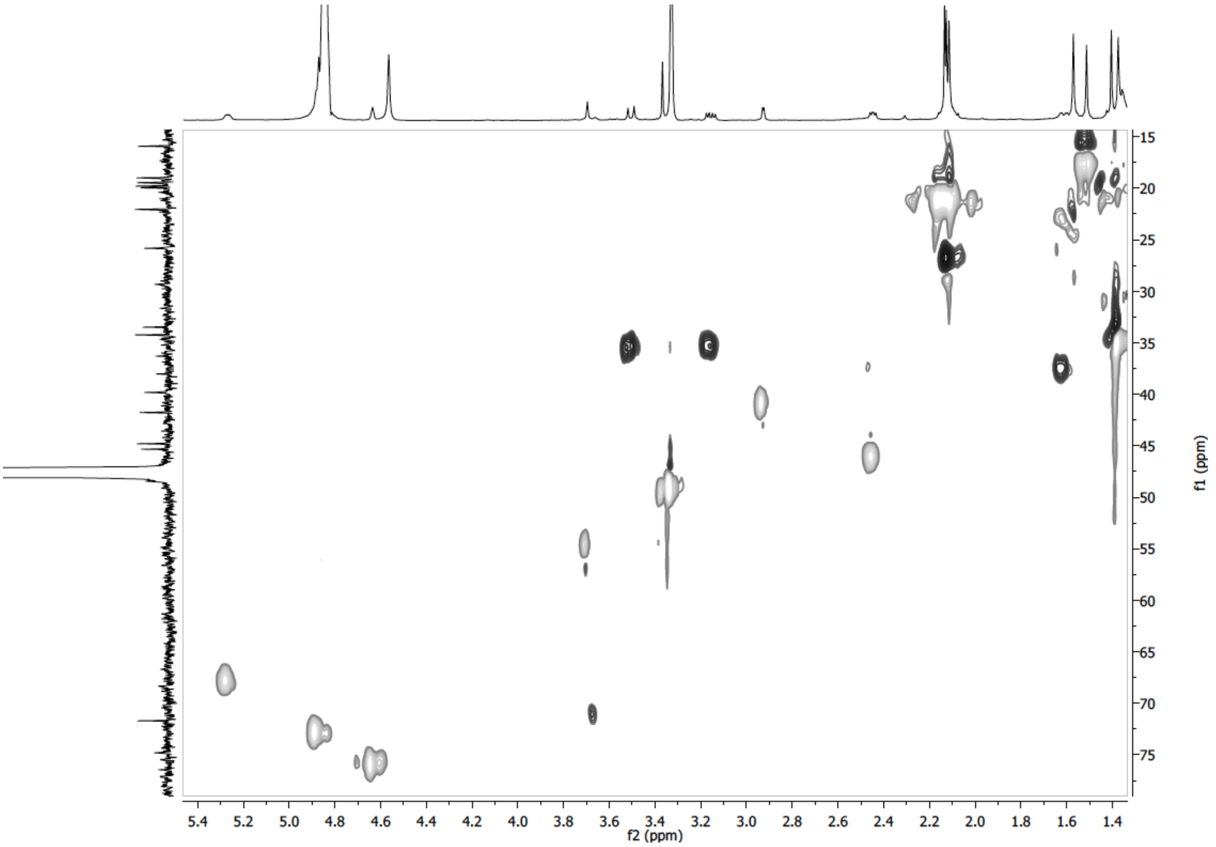

Figure S29. HMBC spectrum of compound **6** (CD_3OD, 600 MHz)

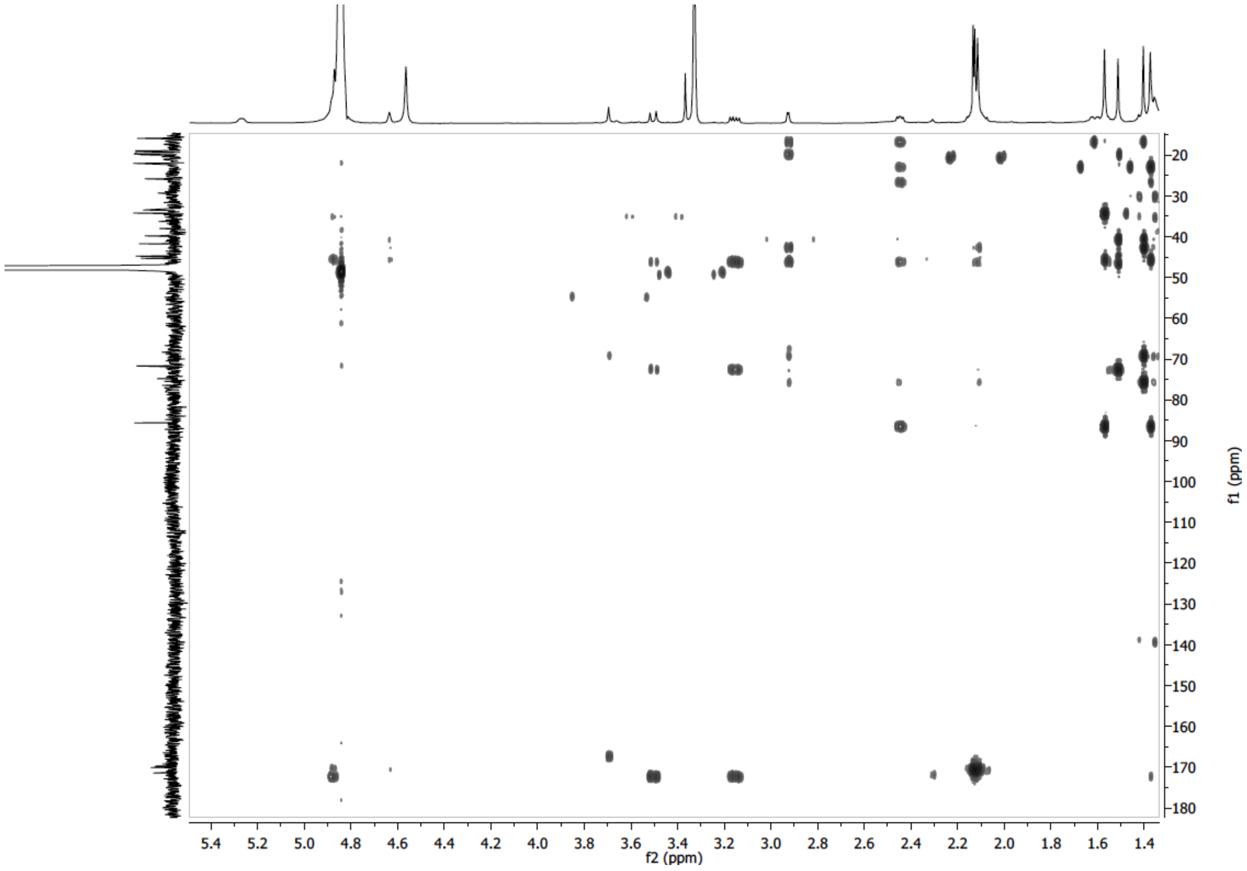

Figure S30. HRESIMS of compound **6**

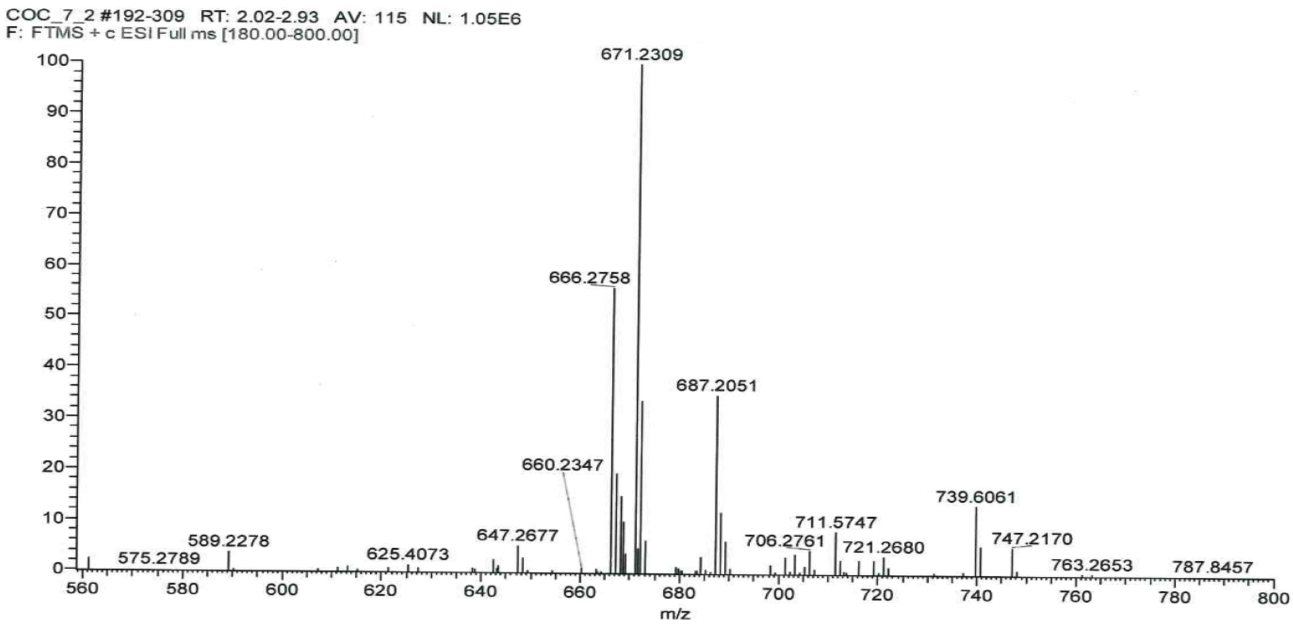

Figure S31. ^{1}H NMR spectrum of compound **7** (CD_3OD, 600 MHz)

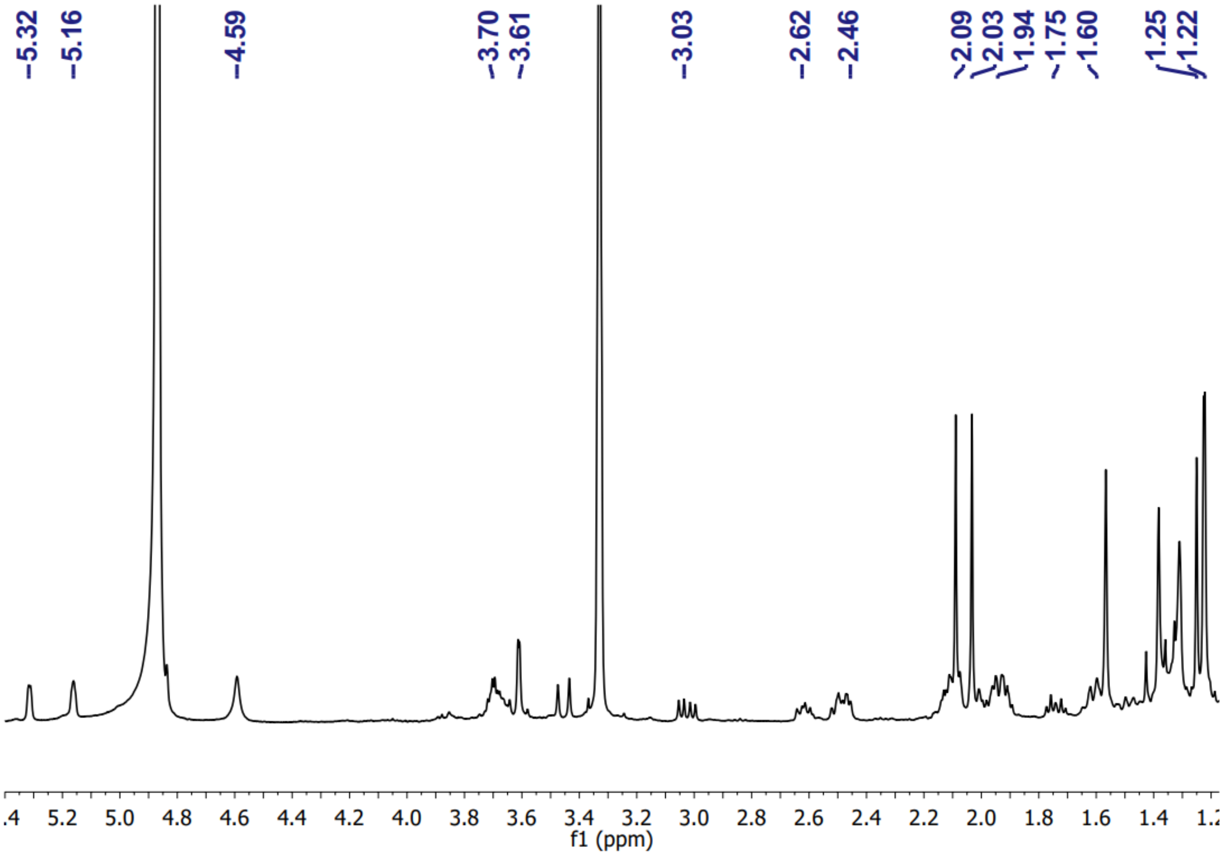

Figure S32. HSQC spectrum of compound **7** (CD_3OD, 600 MHz)

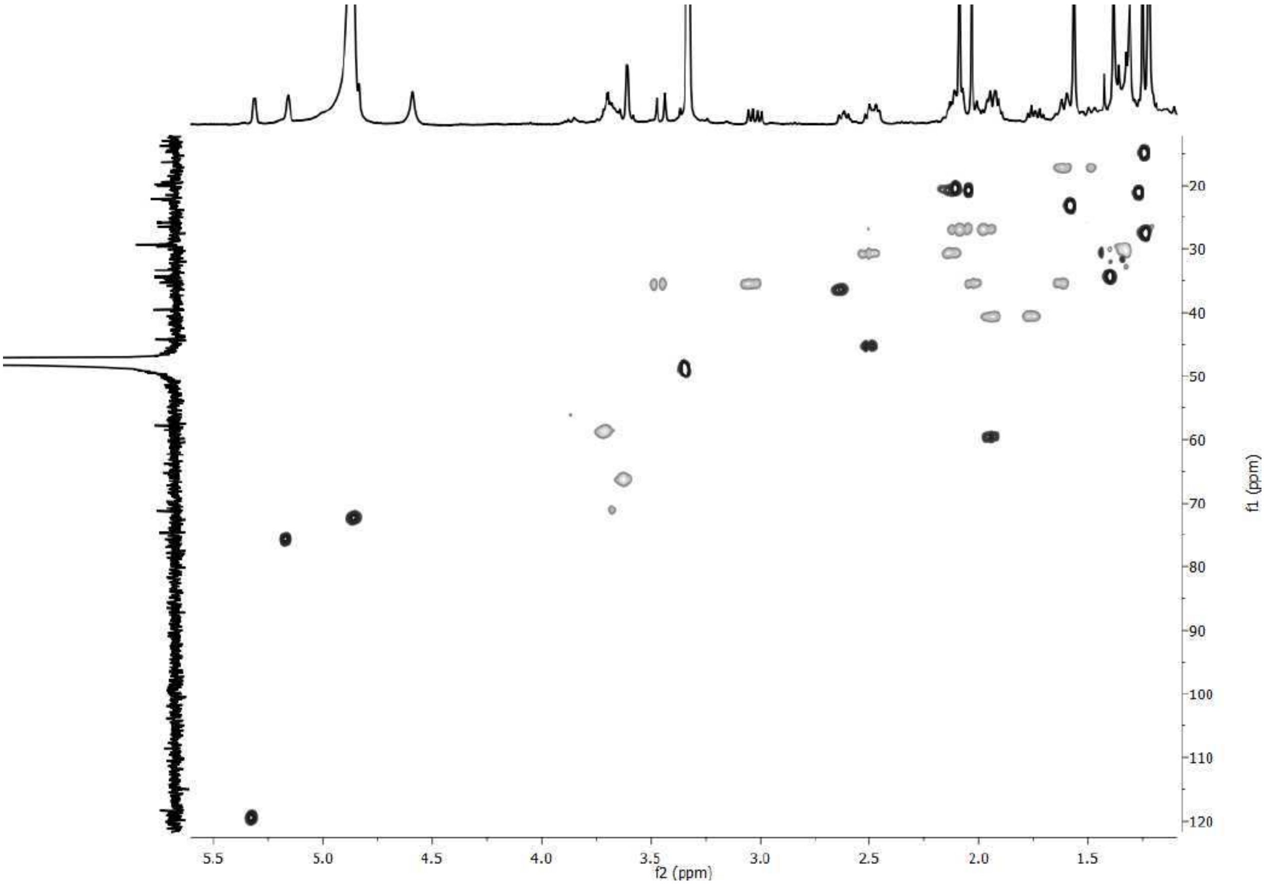

Figure S33. HMBC spectrum of compound **7** (CD_3OD, 600 MHz)

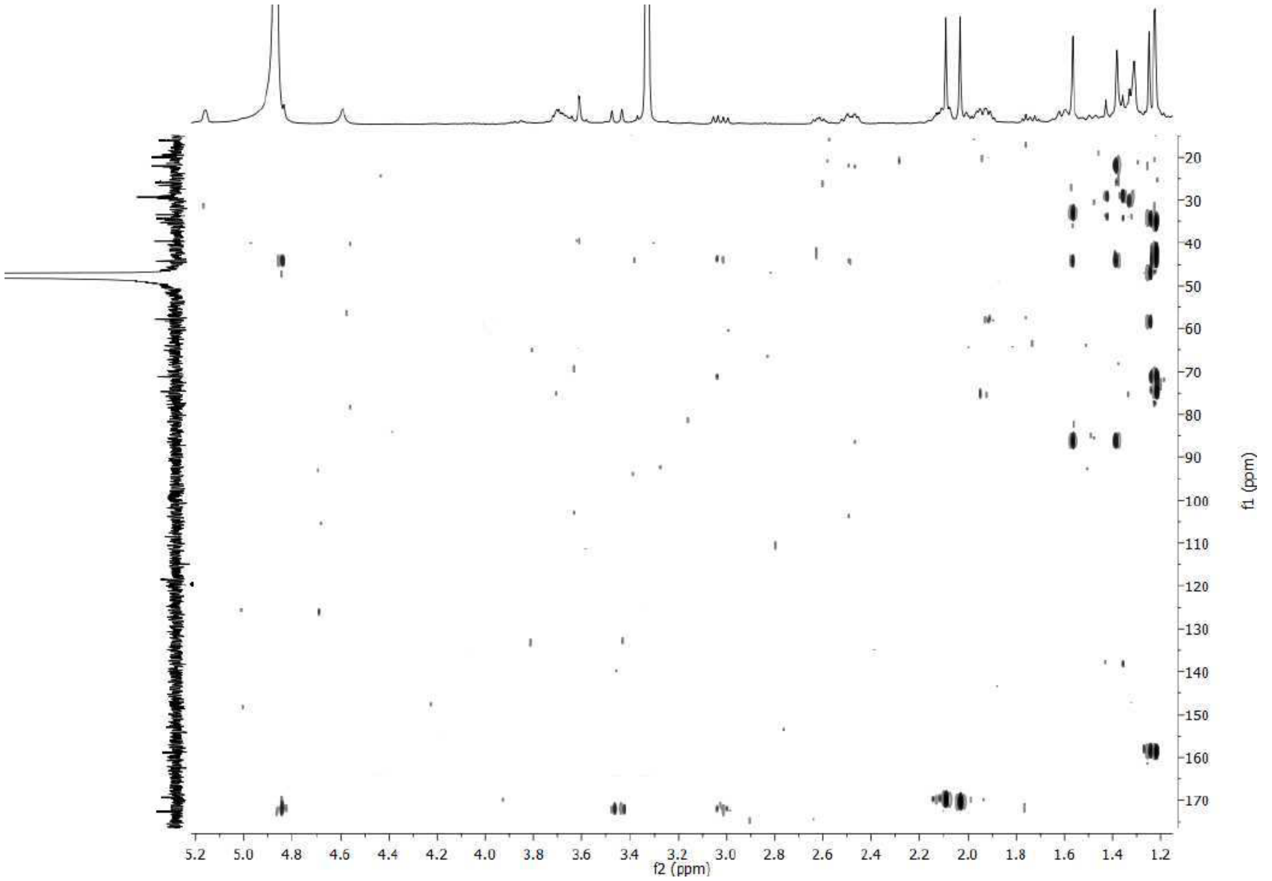

Figure S34. HRESIMS of compound **7**

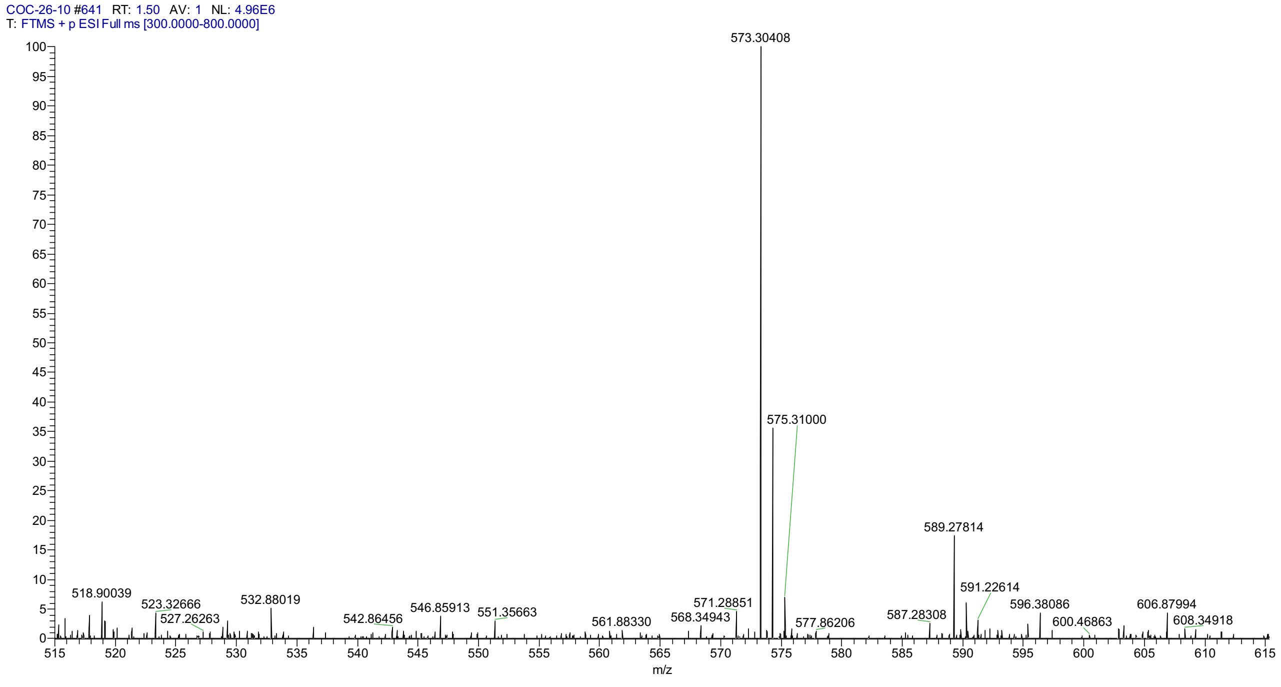

Figure S35. ^{1}H NMR spectrum of compound **8** (CD_3OD, 600 MHz)

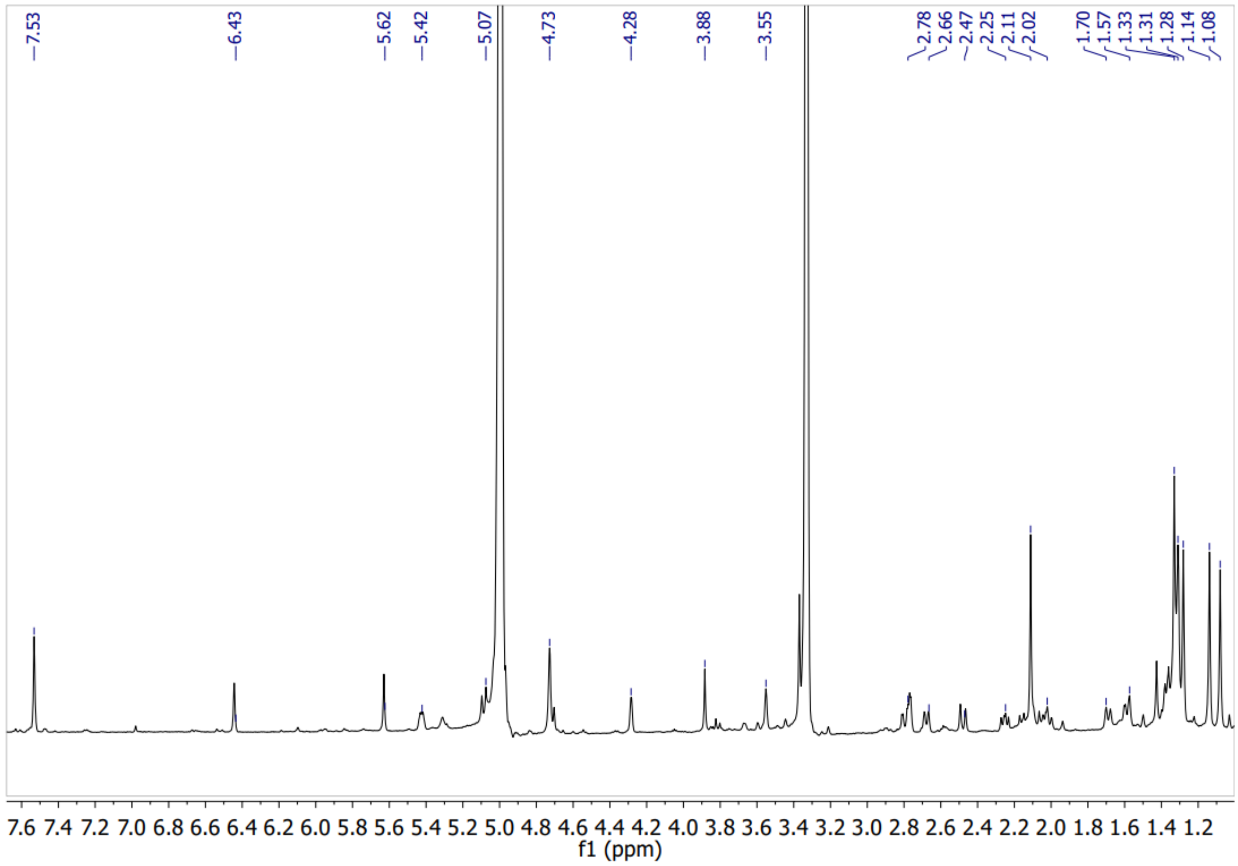

Figure S36. COSY spectrum of compound **8** (CD_3OD, 600 MHz)

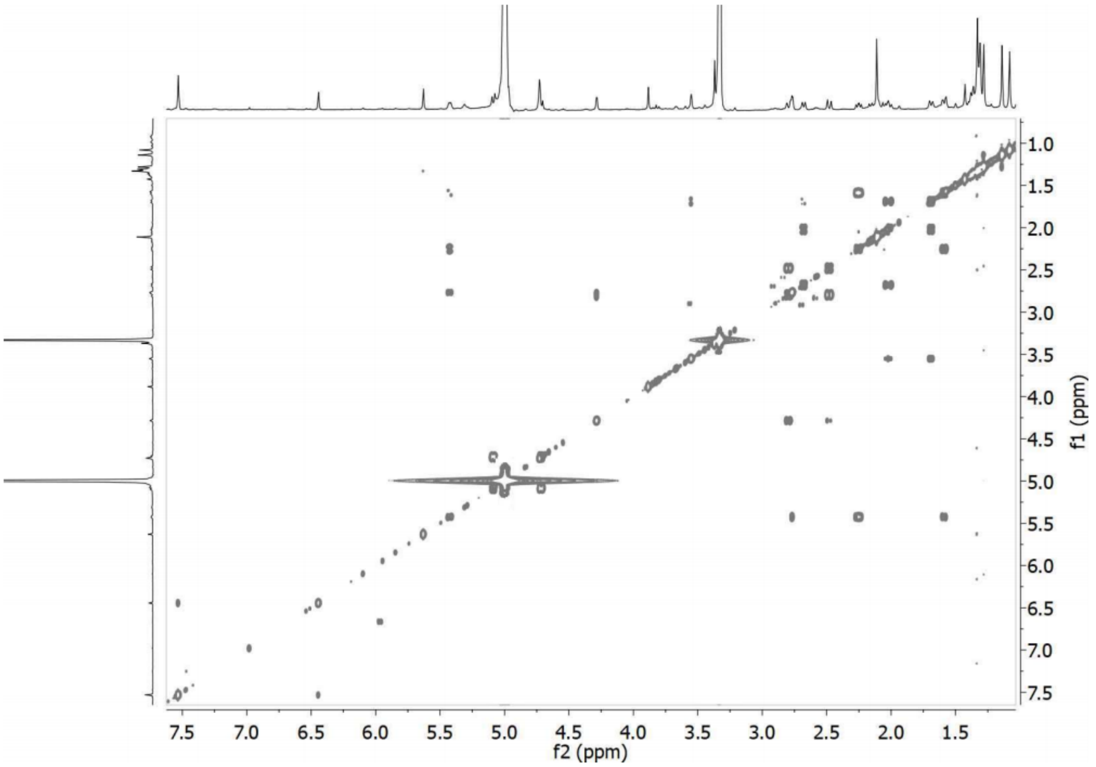

Figure S37. HSQC spectrum of compound **8** (CD_3OD, 600 MHz)

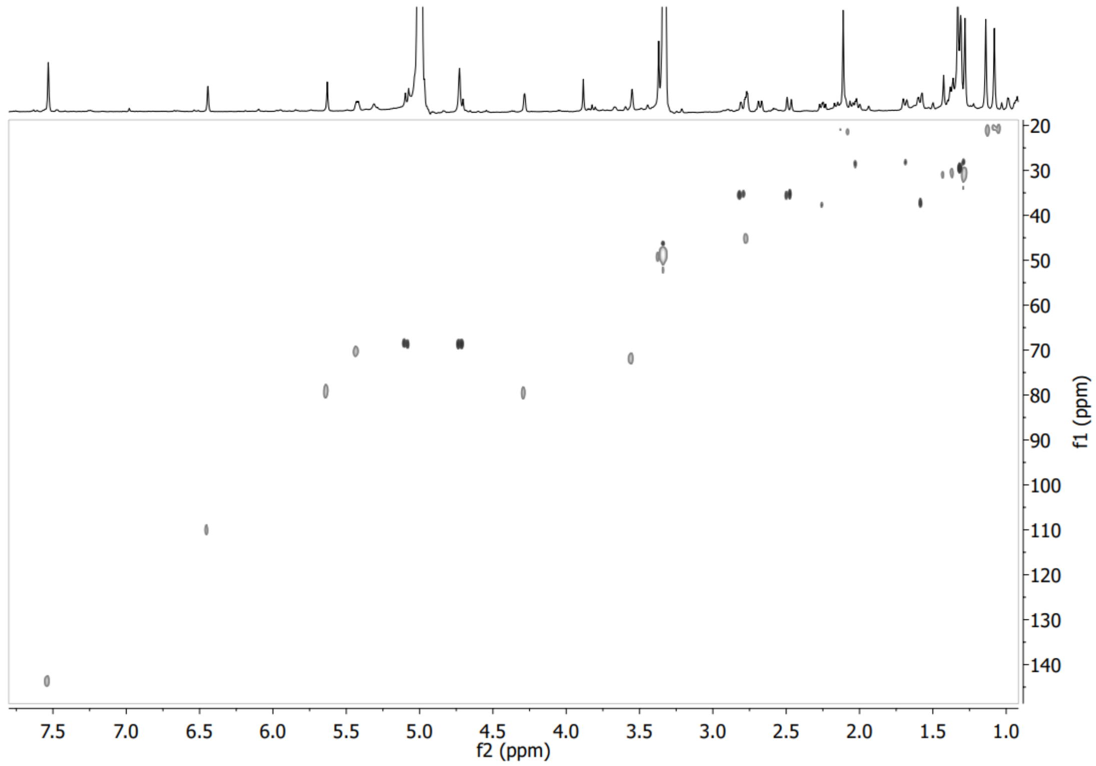

Figure S38. HMBC spectrum of compound **8** (CD_3OD, 600 MHz)

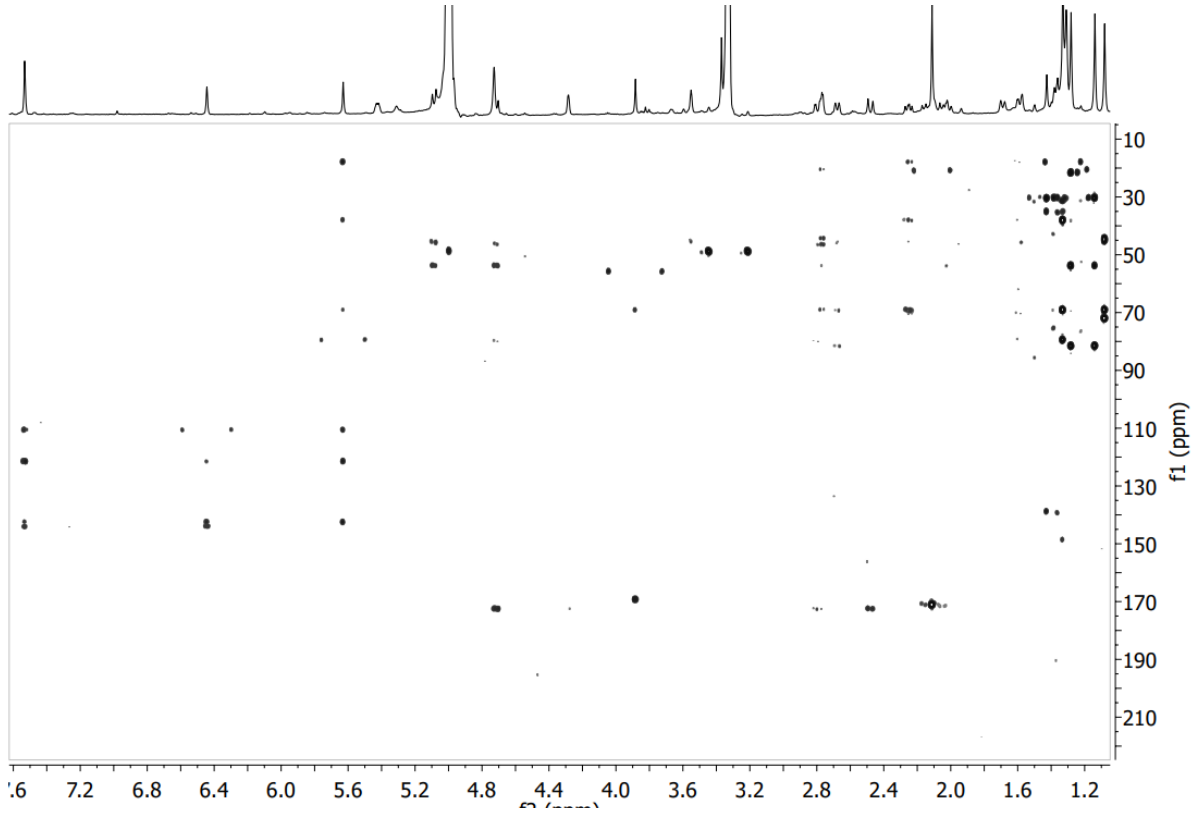

Figure S39. HRESIMS of compound **8**

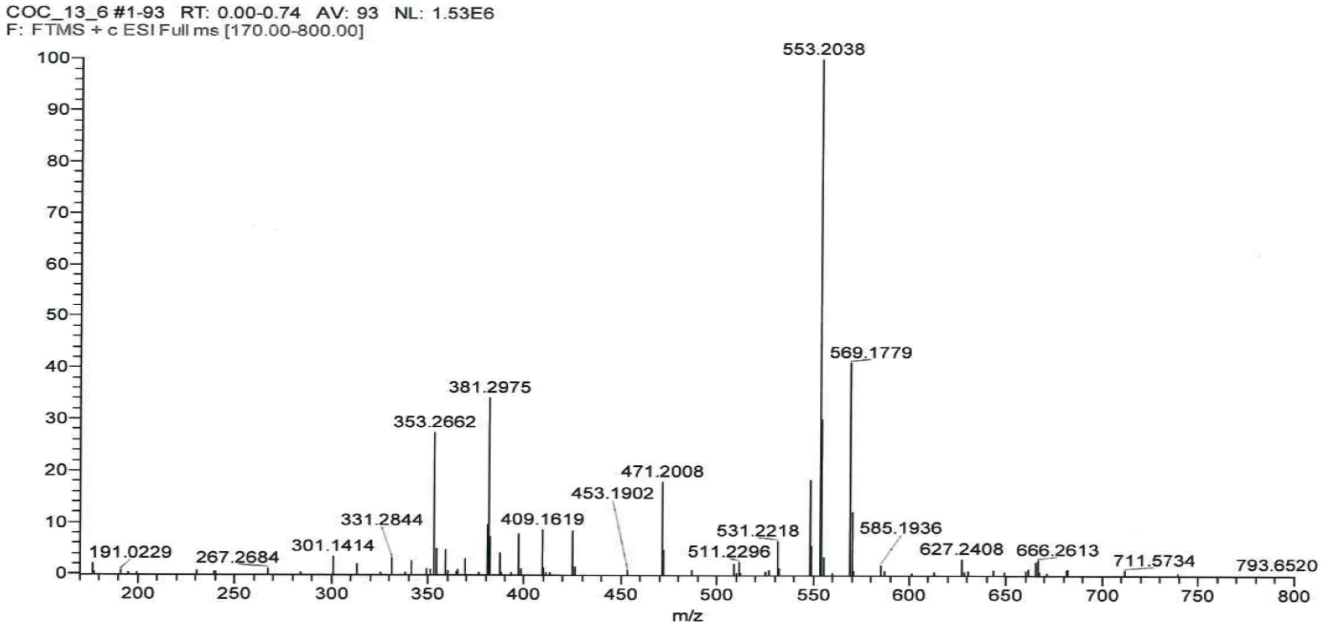

Figure S40. ^{1}H NMR spectrum of compound **9** (CD_3OD, 600 MHz)

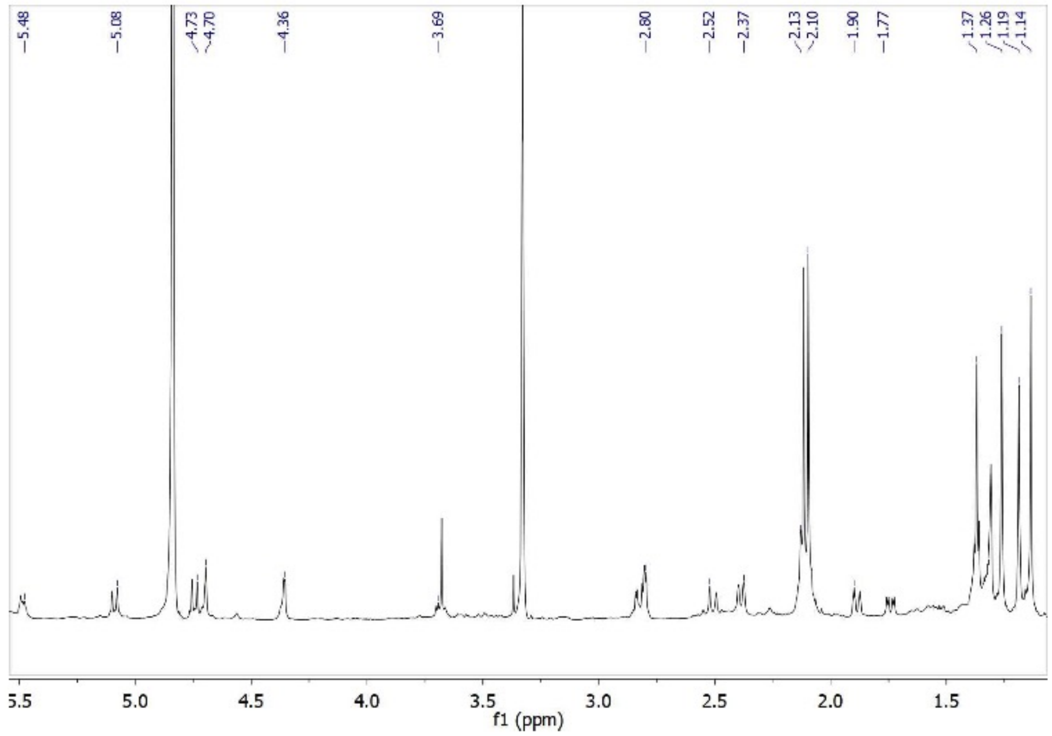

Figure S41. COSY spectrum of compound **9** (CD_3OD, 600 MHz)

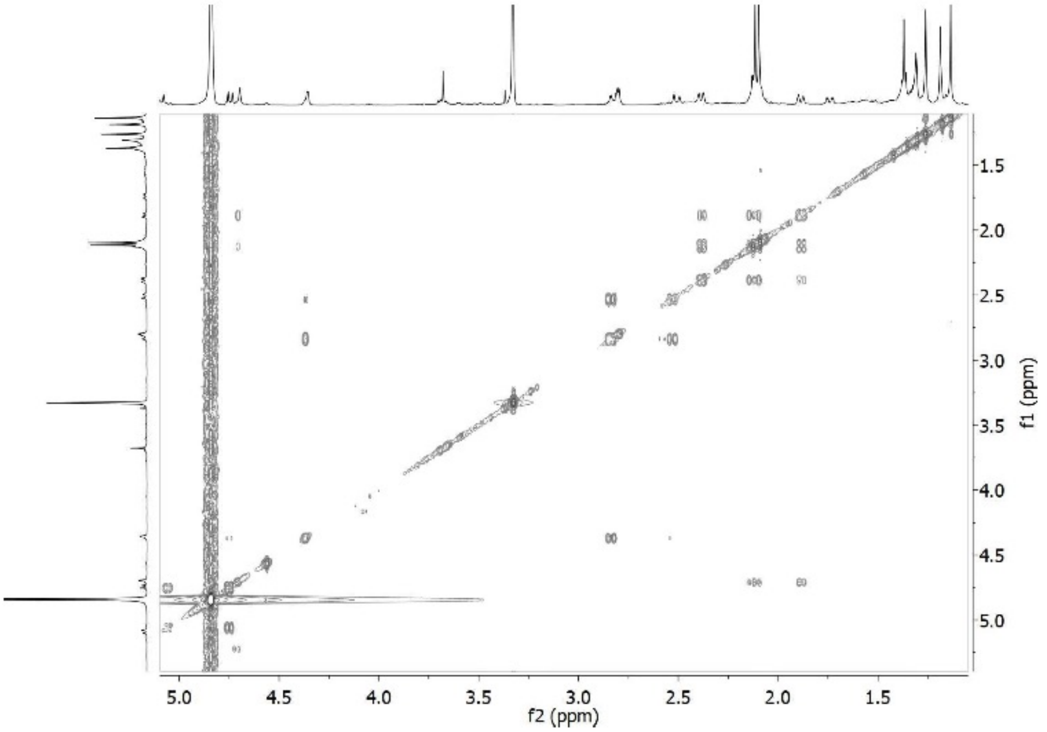

Figure S42. HSQC spectrum of compound **9** (CD_3OD, 600 MHz)

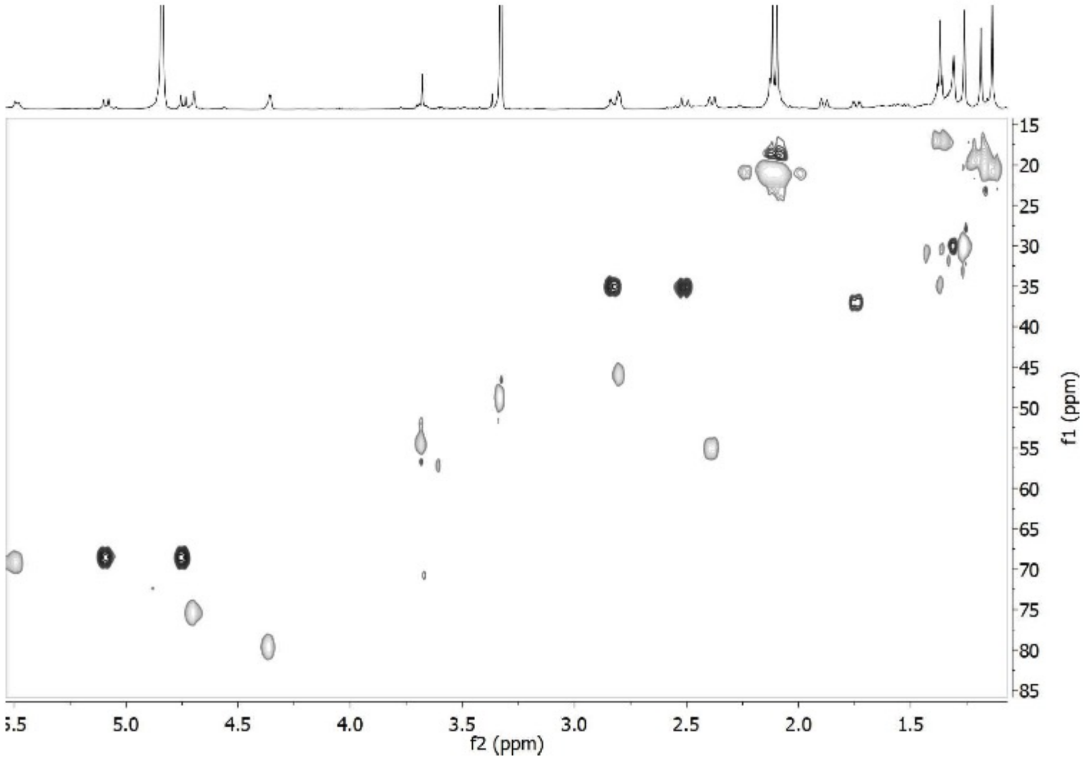

Figure S43. HMBC spectrum of compound **9** (CD_3OD, 600 MHz)

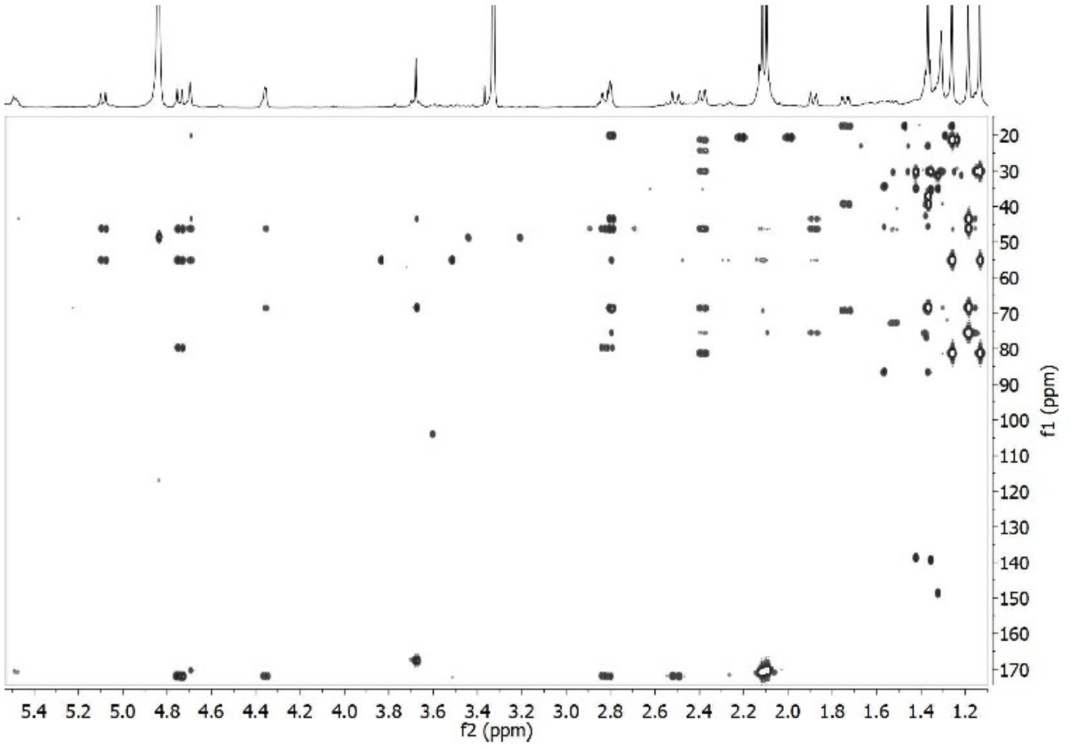

Figure S44. ^1H NMR spectrum of compound **9** ($CDCl_3$, 600 MHz)

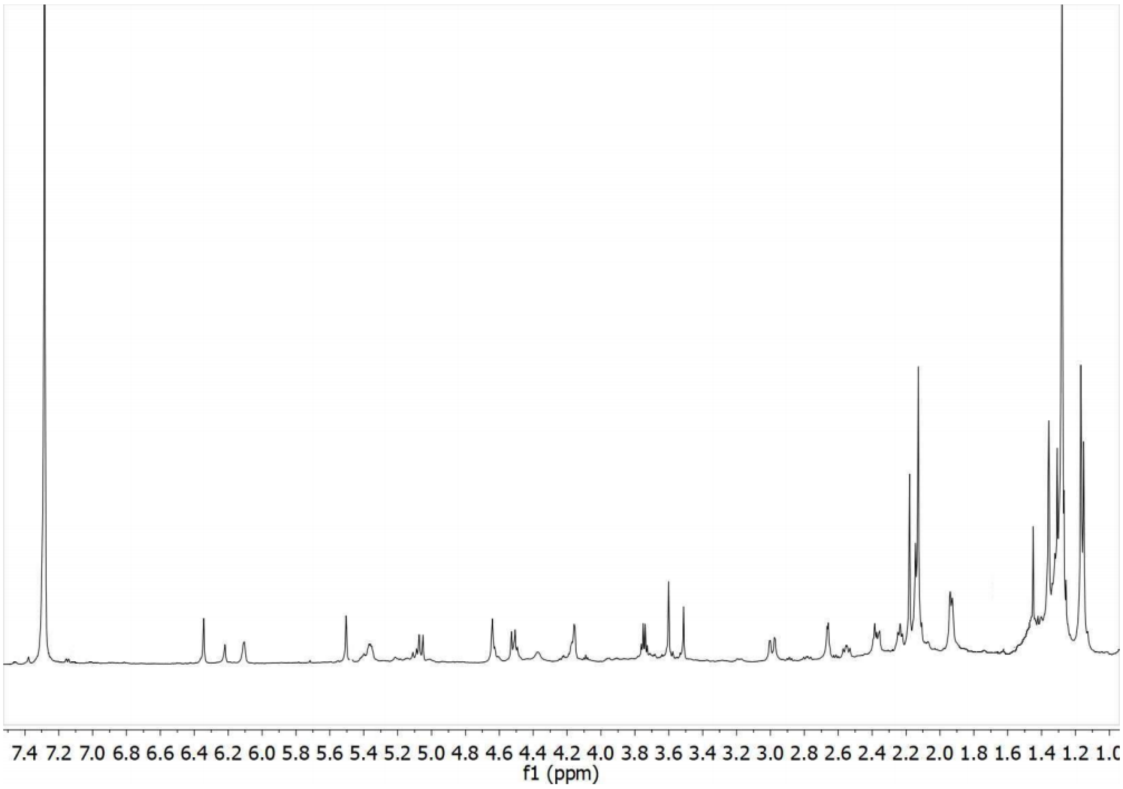

Figure S45. HSQC spectrum of compound **9** ($CDCl_3$, 600 MHz)

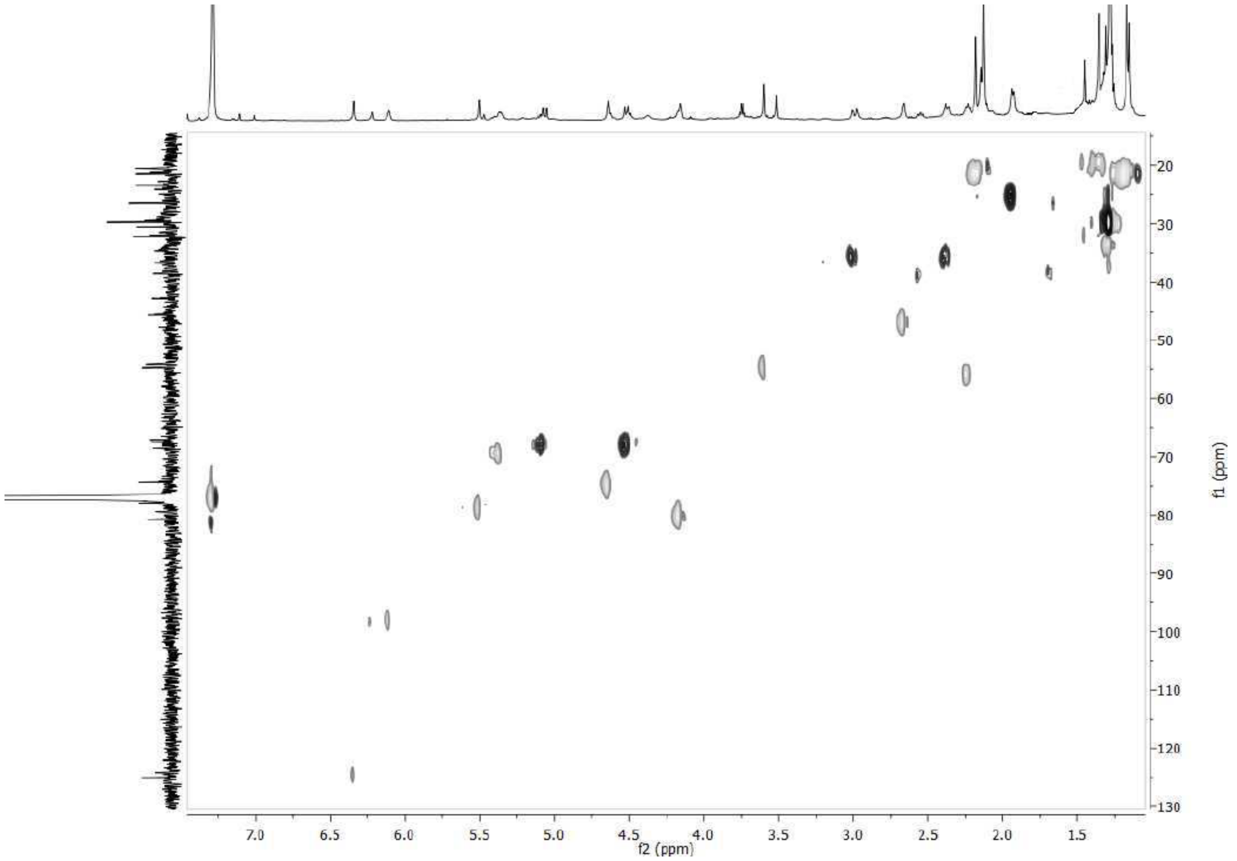

Figure S46. HRESIMS of compound **9**

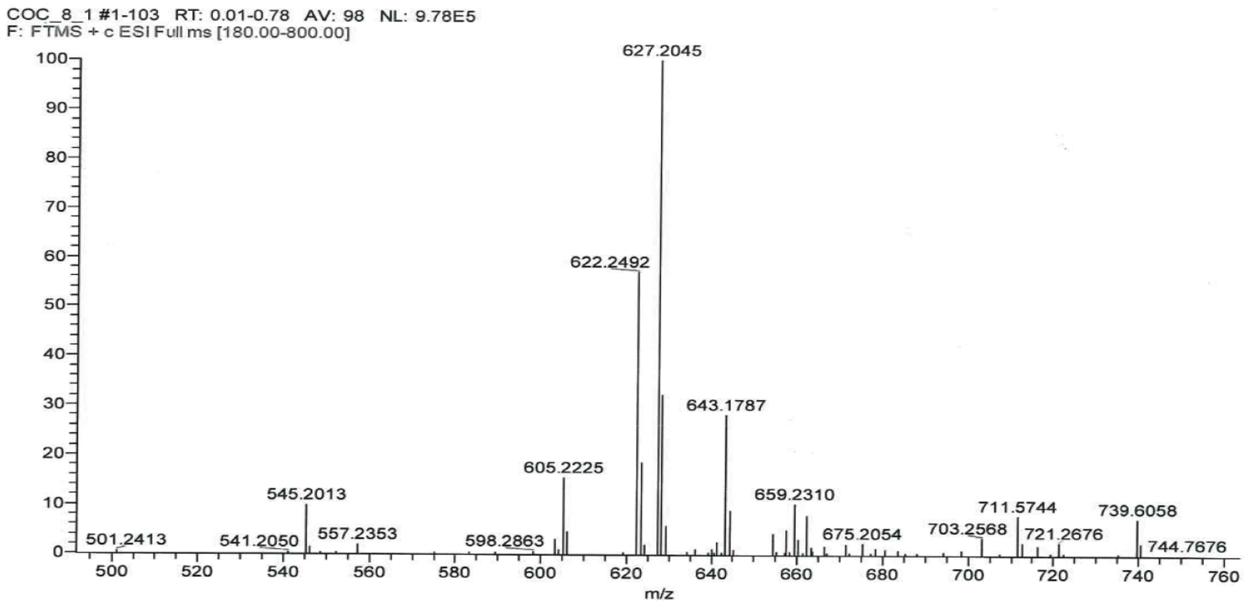

Figure S47. Western blot analysis of p-CDC2 and CDC2 proteins in cell treated with vehicle (DMSO) or radicicol. Normalized results of densitometric analysis are reported. The blots are representative of two different experiments providing similar results.

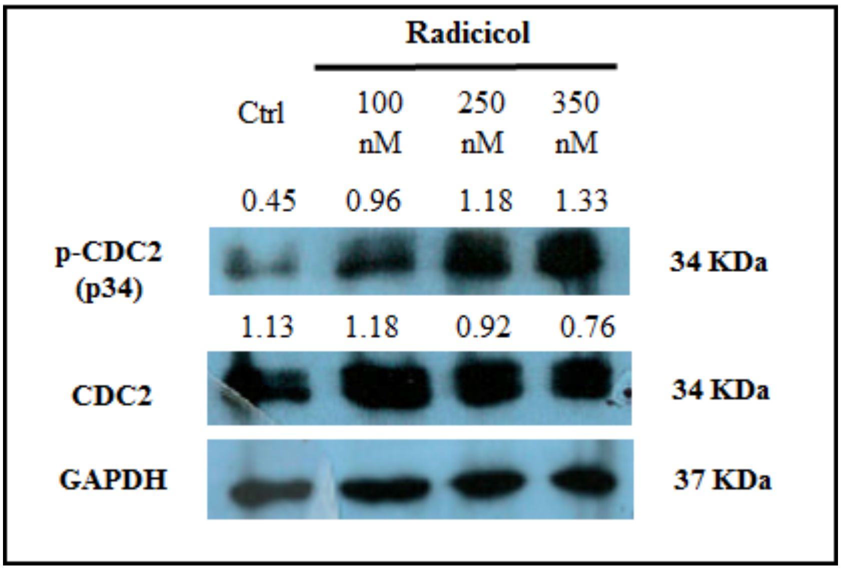

Figure S48. Chemical structures of all the possible diastereoisomers for **1** (**1a-1d**) and **3** (**3a** and **3b**).

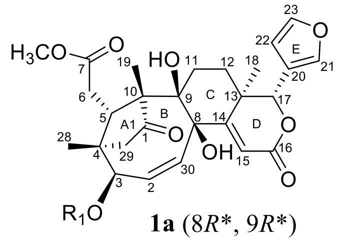

1a (8*R**, 9*R**)

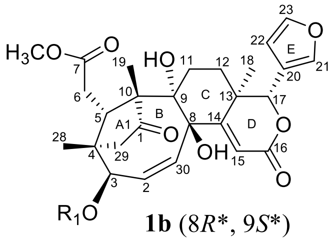

1b (8*R**, 9*S**)

A:

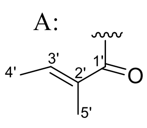

R = A

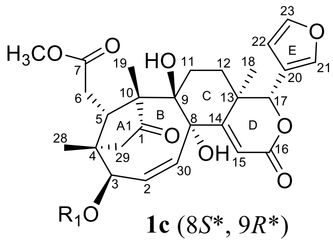

1c (8*S**, 9*R**)

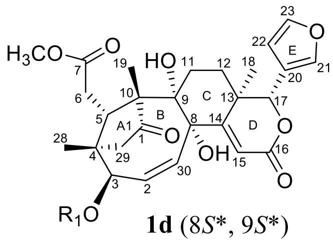

1d (8*S**, 9*S**)

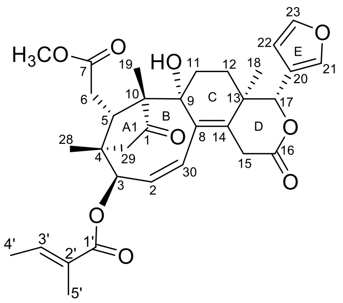

3a (9*R**)

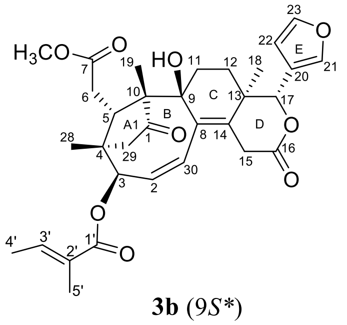

3b (9*S**)

Figure S49. Three dimensional structure of all the possible diastereoisomers for **1** (**1a**-**1d**).

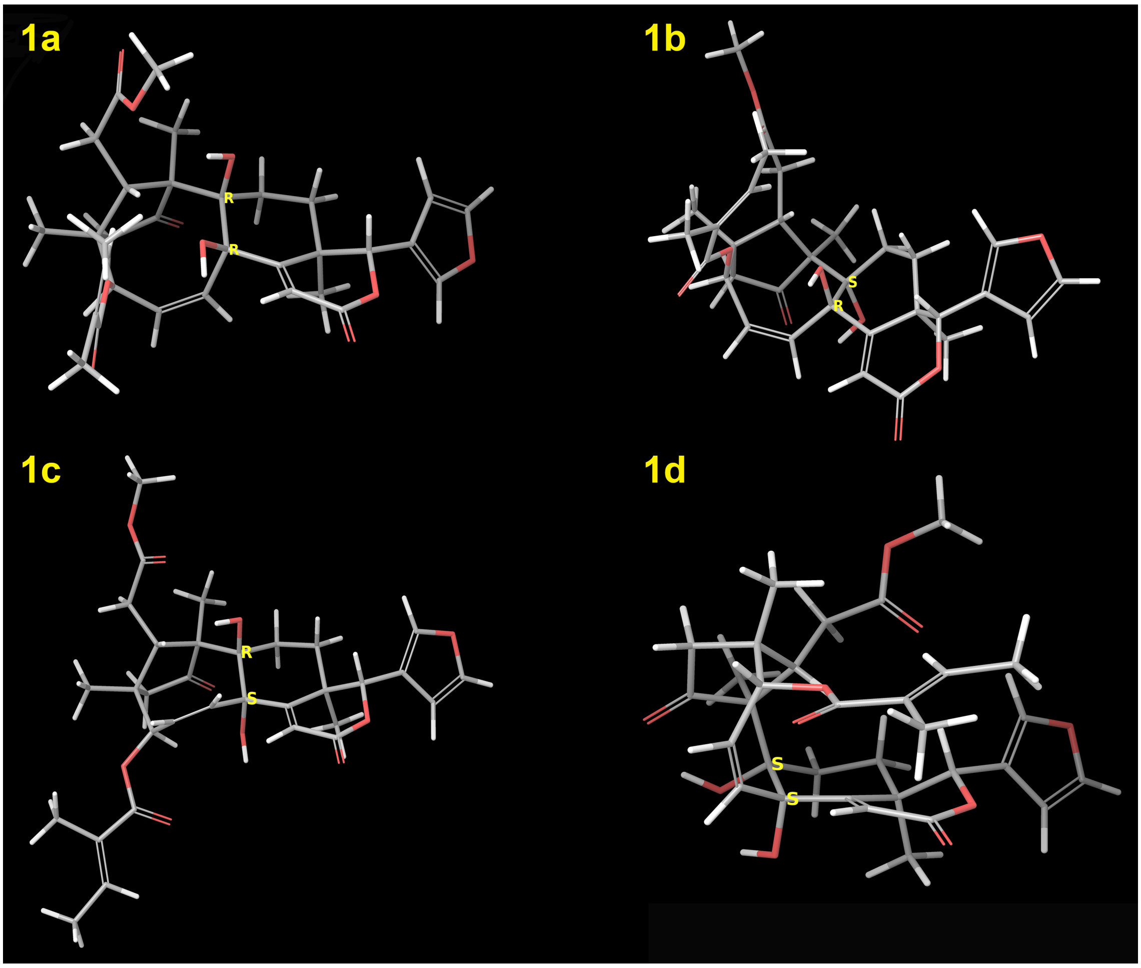

Molecular docking studies of 8, 9, 11 and 13. As reported by us in a previous investigation,[1-3] secondary metabolites containing limonol moiety were disclosed as one most effective classes in compromising the Hsp90α chaperone activity. On these bases, here we reported the molecular docking studies of **8**, **9**, **11** and **13** limonoids (Figure S49), disclosed by Surface Plasmon Resonance (SPR) experiments, which interact with the immobilized chaperone. By our computational analysis, the skeleton of **8**, **9**, **11** and **13** similarly interact with the same region of the biological target, mainly establishing hydrophobic interactions with Ala595, Ala597, Arg591, Gln596, Lys594, and Ser657 of chain A, and with Ala595, Arg591, Glu477, Pro504, and Thr502 of chain B. Moreover **8**, **9**, **11** and **13** are involved in a specific hydrogen bond with the backbone of $Leu658_{ChainA}$, namely the CO group at C-16 of **11** and **13**, and the OH group at C-7 of **8** (Figure S49). Furthermore, the OH groups at C-7 of **8** and at C-3 of **13** make hydrogen bonds with the side-chain of $Ser657_{ChainA}$ and $Glu477_{ChainB}$,

respectively, while the OAc group at C-11 together with the OH group at C-21 of **9** establish hydrogen bonds with the side-chain of $Arg591_{ChainB}$. However, even if the different patterns of hydrogen bonds and the number of hydrophobic contacts of **8**, **9**, **11** and **13** with the C-terminal and the middle domain seem to be the ligand–chaperone complex's driving forces, as confirmed by the thermodynamic constants measured by SPR (see Table 6 in the main text), these intermolecular interactions are not able to interfere with chaperone biological activity.

Figure S50. Three-dimensional models of **8** (a, blue sticks), **9** (b, white sticks), **11** (c, light-pink sticks) and **13** (c, purple sticks) with Hsp90α (chain A is depicted in green and chain B in blue).

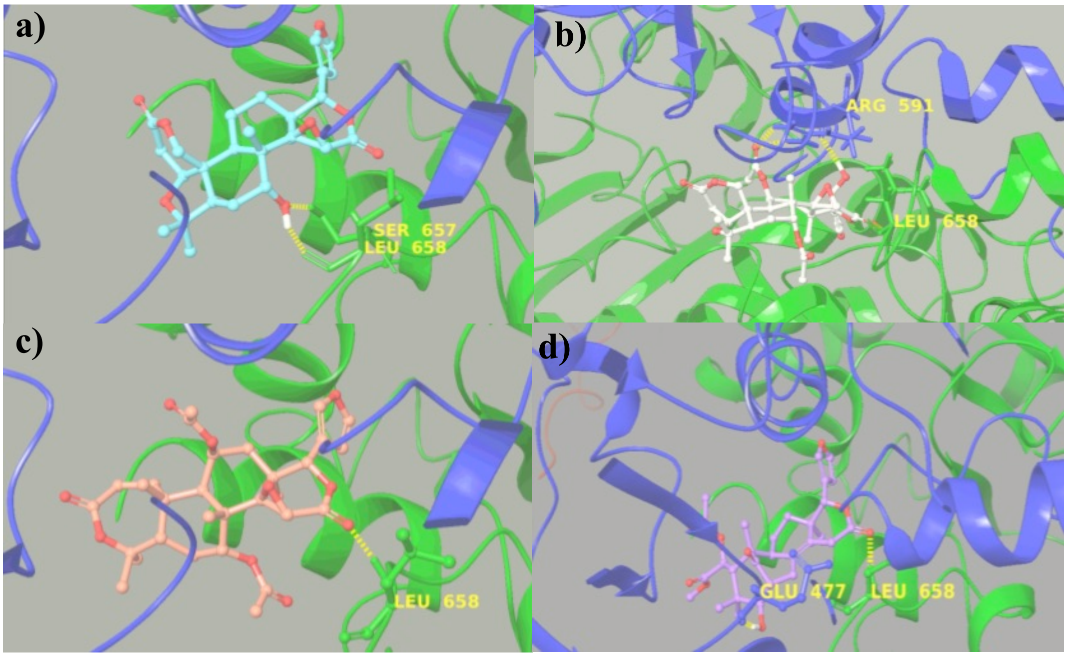

Computational details. Protein 3D model of the ATP-bound active state of Hsp82, a yeast Hsp90α homologue (PDB code: 2CG9) was prepared using the Schrödinger Protein Preparation Wizard workflow.[4] Briefly, water molecules that were found 5 Å or more away from heteroatom groups were removed, and cap termini were included. Additionally, all hydrogen atoms were added, and bond orders were assigned. The resulting PDB files were converted to the MAE format. During the Induced Fit Workflow,[4] the region at the Middle and C-terminal domains interface of Hsp90 (2CG9) were considered as the centroid for grid generation. Ring conformations of the investigated compounds were sampled using an energy window of 2.5 kcal/mol; conformations featuring nonplanar conformations of amide bonds were penalized. The Induced Fit Workflow was performed using the default calculation protocol.

Table S1. Hsp90α peptides detected in the LC/MS analysis of tryptic digested bands at 90 kDa and 70 kDa of SDS-PAGE of the protein extracted from U937 cells treated with compound **1**.

Peptide	Observed ion (*m/z*)	Area Band 90 kDa	Area Band 70 kDa	Area 70 kDa/Area 90 kDa (%)
**42-53**	638.32	798941340	141378066	**18**
**56-64**	520.25	637092076	139520357	**22**
**73-82**	597.83	1177941655	520422529	**44**
**83-95**	683.35	86761859	28503460	**33**
**96-107**	621.84	1543028001	817823858	**43**
**169-177**	476.31	137126423	354037169	**16**
**187-196**	656.29	622043700	172711779	**28**
**205-219**	904.98	39341760	15851010	**40**
**238-249**	744.35	102283698	883505025	**12**
**276-284**	576.28	802967921	200465446	**25**
**285-291**	451.27	483948956	101884436	**21**
**292-306**	924.40	191793430	51149499	**27**
**307-319**	764.38	194179645	154042697	**59**
**320-330**	674.81	103907394	41631989	**17**
**331-337**	415.27	1145084514	292020864	**26**
**338-347**	618.82	430490318	106751086	**25**
**360-378**	1195.57	7910163	2189112	**28**
**379-392**	757.41	1814075983	575150571	**32**
**412-427**	994.63	6807127	11003153	**41**
**429-435**	445.76	1005281092	159378217	**16**
**439-448**	571.81	161102559	159378217	**49**
**450-456**	444.25	148962442	494922565	**35**
**457-475**	731.39	88024084	49340302	**20**
**482-491**	580.80	511225785	128705637	**25**
**492-502**	625.31	545493905	154948104	**28**
**539-550**	708.82	378528743	107293882	**28**
**560-565**	405.70	494005545	141606219	**29**
**584-604**	1224.56	7471397	N.D.	**-**
**625-639**	891.71	14365769	N.D.	**-**
**642-649**	445.36	302052650	N.D.	**-**
**653-679**	996.48	2217088	N.D.	**-**
**686-719**	1178.63	3938623	N.D.	**-**

References

(1) Chini, M. G.; Malafronte, N.; Vaccaro, M. C.; Gualtieri, M. J.; Vassallo, A.; Vasaturo, M.; Castellano, S.; Milite, C.; Leone, A.; Bifulco, G.; De Tommasi, N.; Dal Piaz, F. Identification of limonol derivatives as Heat Shock Protein 90 (Hsp 90) inhibitors through a multisciplinary approach. *Chem. Eur. J.* **2016**, *22*, 13236–13250.

(2) Dal Piaz, D.; Malafronte N.; Romano, A.; Gallotta, D.; Belisario, M.A.; Bifulco, G.; Gualtieri, M.J.; Sanogo, R.; De Tommasi, N.; Pisano, C. Structural characterization of tetranortriterpenes from *Pseudrocedrela kotschyi* and *Trichilia emetica* and study of their activity towards the chaperone Hsp90. *Phytochemistry* **2012**, *75*, 78–89.

(3) Gualtieri, M.J.; Malafronte, N.; Vassallo, A.; Braca, A.; Cotugno, R.; Vasaturo, M.; De Tommasi, N.; Dal Piaz, F. Bioactive limonoids from the leaves of *Azaridachta indica* (Neem). *J. Nat. Prod*. **2014**, *77*, 596–602.

(4) Schrödinger Release 2017-1, Schrödinger Suite 2017-1, LLC: New York, NY, 2017.
